# Noncanonical Amino Acid Tools and Their Application to Membrane Protein Studies

**DOI:** 10.1021/acs.chemrev.4c00181

**Published:** 2024-11-07

**Authors:** Chiara De Faveri, Jordan M. Mattheisen, Thomas P. Sakmar, Irene Coin

**Affiliations:** † Faculty of Life Science, Institute of Biochemistry, Leipzig University, Leipzig 04103, Germany; ‡ Laboratory of Chemical Biology and Signal Transduction, The Rockefeller University, New York, New York 10065, United States; § Tri-Institutional PhD Program in Chemical Biology, New York, New York 10065, United States

## Abstract

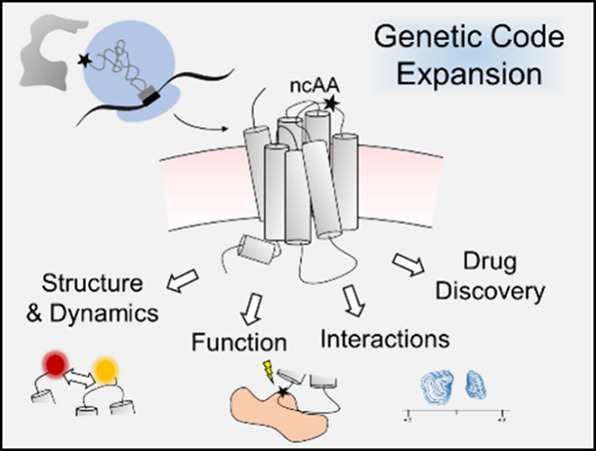

Methods rooted in chemical biology have contributed significantly to studies of integral membrane proteins. One recent key approach has been the application of genetic code expansion (GCE), which enables the site-specific incorporation of noncanonical amino acids (ncAAs) with defined chemical properties into proteins. Efficient GCE is challenging, especially for membrane proteins, which have specialized biogenesis and cell trafficking machinery and tend to be expressed at low levels in cell membranes. Many eukaryotic membrane proteins cannot be expressed functionally in *E. coli* and are most effectively studied in mammalian cell culture systems. Recent advances have facilitated broader applications of GCE for studies of membrane proteins. First, AARS/tRNA pairs have been engineered to function efficiently in mammalian cells. Second, bioorthogonal chemical reactions, including cell-friendly copper-free “click” chemistry, have enabled linkage of small-molecule probes such as fluorophores to membrane proteins in live cells. Finally, in concert with advances in GCE methodology, the variety of available ncAAs has increased dramatically, thus enabling the investigation of protein structure and dynamics by multidisciplinary biochemical and biophysical approaches. These developments are reviewed in the historical framework of the development of GCE technology with a focus on applications to studies of membrane proteins.

## 1 Introduction

**Figure 1:**
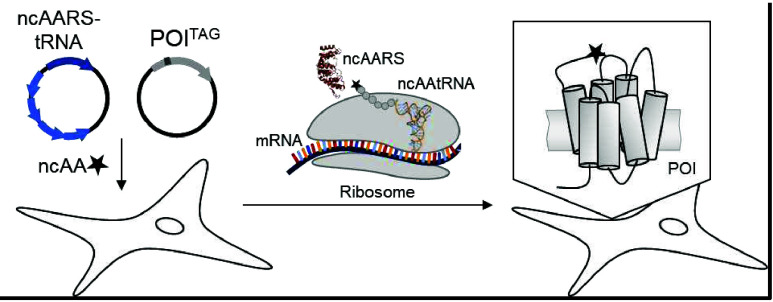
Genetic code expansion strategy for site-specific incorporation of ncAAs into a protein of interest (POI). Cells are transfected with plasmids encoding an aminoacyl-tRNA synthetase (AARS)/tRNA pair and the POI containing an amber stop codon (TAG) at the desired site of modification. In the presence of ncAA, the ncAARS/tRNA pair incorporates the ncAA into the nascent sequence at the site of the TAG, resulting in stop codon readthrough and full-length protein.

The concept of genetic code expansion (GCE, Figure 1) was conceptualized in the early molecular biology era, but the practical widespread use of GCE, especially for studies of membrane proteins has developed only during the past two decades. A seminal early report showed that it was possible to engineer the cellular protein synthesis machinery to create an orthogonal amino acyl-tRNA synthetase (AARS)/tRNA pair that could recognize an exogenously added noncanonical amino acid (ncAA) and suppress an amber stop codon introduced using site-directed mutagenesis.^1^ Ideally, the resulting protein would include the ncAA exclusively at the specified position. Early versions of GCE overcame the difficulty of engineering the AARS/tRNA pair and expressing genes encoding the pair in target cells.^2−4^ Using this initial GCE strategy, more than 200 different ncAAs have been incorporated into proteins in a variety of systems (e.g., *E. coli*, mammalian cells in culture, yeast and multicellular organisms). The functionalities of these ncAAs vary from direct biochemical and biophysical probes to bioorthogonal reactive functional groups useful to selectively “click” desired probes to the protein of interest post-translationally.^5−7^

For example, the side chains of ncAAs can be designed to be chromophores, fluorescent donors or acceptors, infrared (IR) probes, nuclear magnetic resonance (NMR) and electron paramagnetic resonance (EPR) probes, among others. When practical, the direct introduction of small-molecule probes is preferable to using large fusion protein constructs such as, for instance, green-fluorescent protein (GFP) or its derivatives for fluorescence labeling, as these often perturb protein function. In addition, ncAAs carrying a bioorthogonal handle for a cell-compatible conjugation reaction, such as azides, alkenes or alkynes enable the introduction of a huge variety of tailor-made probes for the desired experiment in an engineered protein. Indeed, the invention of bioorthogonal chemistries to facilitate the secondary labeling of engineered proteins under physiological conditions has been a key enabling development of GCE.^8,9^

Another important technique with a rich history in protein chemistry that is dramatically enabled with GCE is photochemical cross-linking. Cross-linker ncAAs include moieties such as an azide, benzophenone or nitrile, among others, that are inert under physiological conditions but can be photolyzed so that they become highly reactive and form covalent bonds with interaction partners in the vicinity. Since the location of the ncAA within the protein of interest is known *a priori*, the method is also known as “targeted photo-crosslinking”. Especially when combined with knowledge about the specific photochemistry of the photoreactive moiety, targeted photo-cross-linking can often provide useful information about protein–ligand interactions or intra- and intermolecular protein–protein interactions.

One of the most attractive features of GCE is that the artificial probe can, in principle, be placed anywhere in the amino acid (AA) sequence of the protein under study, which is difficult to achieve using semisynthetic methods. Furthermore, since the location of the ncAA is under molecular genetic control, the effect of the ncAA substitution can be screened in cell-based *in vitro* systems. In many cases, the ideal location for a chemical probe is one in which the modified protein is fully functional, yet the probe is sensitive to environmental or dynamic conformational changes under study. Thus, GCE can serve as a useful alternative to traditional loss-of-function mutagenesis studies, where the role of a specific AA residue is inferred from a loss-of-function observed when it is substituted.

In this review, we report on state-of-the-art GCE techniques for studying membrane proteins. We will also attempt to provide guidance on which GCE-enabled tools are relevant for the study of specific chemical and biological questions. In the first main section, we will discuss the chemical biology tools that are available to enable GCE and provide an excursus on classical techniques to introduce tags and probes into proteins. In the second main section, we will discuss how biological questions have been addressed using GCE strategies. One particular focus is to describe how GCE can be used to expand the scope of drug discovery at membrane protein targets. It will become clear that advances in GCE technology have benefited from interdisciplinary teams of biologists and chemists, because, in principle, the variety of ncAAs that can be used to engineer proteins is almost limitless.

## 2 The ncAAs and the ncAA-Incorporation Systems

### 2.1 Orthogonal AARS/tRNA Systems for ncAA Incorporation

The concepts of chemical and biological orthogonality and their practical application are fundamental to GCE. For a particular ncAA to be incorporated into a protein at a defined codon position, an AARS/tRNA pair orthogonal to the endogenous protein translational machinery of the cell system of choice is needed. First, the orthogonal AARS should selectively bind to the desired ncAA and not to canonical AAs. Second, the AARS should be able to differentiate between its cognate amber suppressor tRNA and the endogenous ones, while only amino-acylating the former. Similarly, the tRNA also needs to be orthogonal to the host system and must not be aminoacylated by any endogenous AARS.^10,11^ The introduction of engineered AARS/tRNA pairs originating from an organism belonging to a different kingdom than that of the cell system of choice often guarantees the orthogonality between the translational pair for the ncAA and the endogenous translational pairs.

Ideally, the anticodon of the orthogonal tRNA used for GCE should be an “empty” triplet that introduces the ncAA only at the desired position in mRNA-guided protein synthesis. The 64 possible triplet codons encode only elongation signals for 20 (or in some cases up to 22) AAs and a translation termination signal, or nonsense codon. The initiation codon also encodes Met. However, the genetic code is degenerate, all 64 codons are assigned, and “empty” triplets are not available. Nonsense suppression (i.e., inhibition of translation termination at stop codons) due to mutations in tRNA genes that alter codon-anticodon interaction was described at the dawn of the molecular biology era.^12^ Nonsense suppressors are usually caused by changes in the tRNA anticodon stem of certain tRNAs that allow them to read through the amber stop codons (UAG). For example, in *E. coli*, either Ser, Gln, Tyr or Leu tRNAs can be altered to suppress nonsense amber codons. In addition, Tyr-tRNA from *E. coli* was shown to result in amber nonsense suppression when introduced into mammalian COS-1 cells, an important observation that facilitated development methods to introduce ncAAs into membrane proteins in mammalian cells.^13,14^ Fortuitously, and perhaps also because it is the most frequently suppressed, the amber codon is used most infrequently among the three nonsense codons. For all the above reasons, the codon repurposed for GCE applications is generally the amber stop codon, although a few systems have been described that suppress the ochre (UAA) or opal (UGA) codons.

An inherent limitation of attempting to repurpose existing codons for GCE is that the endogenous release factors will necessarily compete with the suppressor tRNA.^15^ Different strategies have been pursued to overcome this issue. On one hand, efforts have been made to outcompete the releasing factor, either by pushing the overexpression of the suppressor tRNA to the highest level through usage of multiple copies of tRNA expression cassettes on separated plasmids^14,16^ or through the development of tRNAs featuring higher stability and efficiency, such as the tRNA^PylM15^^17^ and, most recently, other tRNAs.^18,19^ On the other hand, bacterial strains have been generated that lack the release factor 1 (RF1), which terminates translation at the amber codon.^20^ Eukaryotes have only one release factor (eEF), which is obviously indispensable. Therefore, a strategy was developed to modify eEF so that it would not recognize amber stop codon or suppress its termination.^16,21^ Despite these advances, stop codon suppression is not quantitative.

Conceptually, reassigning a stop codon, even a relatively infrequently used one like the amber codon, has other inherent limitations. First, there is still a subpopulation of endogenous proteins in the system of choice (be it bacteria or yeast or mammalian), whose termination depends on the chosen stop codon, and it is still difficult to predict the side effects that stop codon suppression can have in the cell. It is a matter of fact that there still do not exist robust stably transfected mammalian cell lines for general use for stop codon suppression, although some mammalian cell clones for GCE have been recently published.^22^ Another limitation resides in the fact that there are only three stop codons available for potential use in GCE. The freedom to incorporate multiple ncAAs using nonsense suppression is therefore limited to only two even in the best-case scenario since one stop codon still needs to be used for translation termination.

Significant efforts have been made to engineer tRNAs in which the anticodon loop is expanded to a quadruplet.^23−25^ In fact, there are examples in nature where a quadruplet codon encodes for an endogenous AA.^26,27^ The Lys-tRNA synthetase from *Pyrococcus horikoshii* was introduced in bacteria to incorporate l-homoglutamine (hGln **162**) in response to the AGGA codon.^28,29^ To increase incorporation yields when using the quadruplet codon strategy, efforts were made to generate orthogonal ribosomes in *E. coli* able to accommodate quadruplet anticodon tRNAs.^30^ Recently, a strategy to reassign codons was published, involving hyperaccurate ribosome mutants, which allow for the incorporation of different AAs under reassigned codons.^31^ Nevertheless, the amber codon remains the overwhelming choice for most GCE applications.

Among the hundreds of ncAAs that have been introduced into proteins of interest using GCE, the vast majority are analogues of Tyr or Lys because the AARSs that recognize them are derived from the adaptation and development of Tyr and Pyl orthogonal AARS/tRNA pairs, respectively. Overall, for GCE in bacteria and mammalian cells, two main systems are being used. The Tyr system, either from *Methanococcus jannaschii* (for use in bacteria) or from *E. coli* (for use in yeast and mammalian cells) and the *Methanosarcina* PylRS (from *Methanomethylophilus alvus Ma*, *Methanosarcina barkeri Mb* or *Methanosarcina mazei Mm*), which is orthogonal in *E. coli*, mammalian cells and in yeast. To further increase the number of orthogonal systems for amber suppression in *E. coli*, the *Saccharomyces cerevisiae* TrpRS/tRNA^Trp^ pair was also modified to be able to incorporate a variety of Trp derivatives in bacteria.^32^ Other systems have also been developed to incorporate ncAA analogues of Leu, Trp and Ser among others. For example, orthogonal AARS/tRNA pairs have been developed for the ncAA O-phosphoserine (Sep **163**), which has particular biological interest.^33^

A relatively small number of AARS/tRNA systems are available because of the significant effort required to develop and benchmark them experimentally. To generate an AARS capable of incorporating a given ncAA, the binding pocket of the enzyme must be engineered to accept the ncAA selectively and to ensure that the AARS/tRNA does not incorporate endogenous AAs. This is achieved through a series of positive and negative selections on AARS libraries designed by randomizing via saturation mutagenesis of a cluster of five to seven residues in its binding pocket.^34^ Just a few AA substitutions in the substrate binding pocket of the AARS can result in very different affinities for ncAAs, and the effects of the mutations are relatively easy to assess.^35^ To our knowledge, computational design of specific AARSs remains extremely challenging, although some examples have been reported.^36^ The development of novel GCE systems is typically followed by a lag phase enabling reagents such as probes with compatible reactive groups to be prepared, tested, and in some cases made commercially available. In most cases, this process occurs while iterative improvements are made either to increase the orthogonality and selectivity of the AARS for a particular ncAA of interest or to increase heterologous tRNA expression.^37,38^ AARS/tRNA pairs and their corresponding expression vectors also need to be engineered for optimal function in the genetic system of interest (e.g., *E. coli*, yeast, mammalian cells). As described below, the PylRS system is the most versatile and flexible, especially because of the large number of structurally diverse ncAAs that it can recognize productively.

#### 2.1.1 TyrRS and PylRS

In the early stages of the development of practical GCE tools, libraries of the archaebacterium *Methanococcus jannaschii* TyrRS were generated for the incorporation of several Tyr analogues in *E. coli*. Given the size and polarity of the endogenous Tyr substrate, a variety of different ncAAs could be selectively incorporated by engineered TyrRS variants.^39,40^ The *Mj*TyrRS/tRNA was the first system imported in *E. coli*, and the accurate optimization of the tRNA made it even more orthogonal in bacteria.^3,4^ In later years, mutations of the *Mj*TyrRS/tRNA system were done to improve its stability and yields of ncAA incorporation, resulting in the widely used inducible pEVOL system,^41^ which allows for the incorporation of dozens of ncAAs. Furthermore, variants of the *Mj*TyrRS were identified, which are orthogonal to endogenous AAs in *E. coli*, but could not distinguish well between similar ncAAs. One example of an AARS with polysubstrate specificity is the *Mj p*-cyanophenyl-Ala RS (*p*CNF-RS). Its polysubstrate specificity is due to the increased size of the binding pocket caused by the mutation of 6 positions (Y32, L65, F108, N109, D158, I159) and the removal of residues capable of hydrogen bonding (necessary for Tyr binding).^42^ Since the desired ncAA is added to cell culture media, polysubstrate specificity can be an advantage because it allows several different Tyr analogues to be incorporated using the same AARS/tRNA pair.

The *Mj*TyrRS/tRNA cannot be used for incorporation of Tyr-like AAs in yeast and mammalian cells because it is not orthogonal in these hosts.^43,44^ On the other hand, the prokaryotic TyrRS and LeuRS from *E. coli* are orthogonal in eukaryotes.^3,45−47^ Thus, *Ec*TyrRS and *Ec*LeuRS clones were engineered and selected in yeast to genetically encode ncAAs, including bulky and fluorescent AAs.^10,48,49^ The TyrRS recognizes the UAC anticodon in the tRNA^Tyr^ and has lower affinity for the modified tRNA^Tyr^ that carries the UAC anticodon. This drawback can be compensated by introducing suitable mutations in the AARS that improve the recognition of the mutated anticodon.^50^ Classically, AARS selected in yeast are subsequently shuttled to mammalian cells. Here, the *Ec*TyrRS is ideally paired to a suppressor tRNA derived from the tRNA^Tyr^*Bacillus stearothermophilus* (usually named as *Bst*Yam):^10^ This prokaryotic tRNA contains the so-called A- and B-box (internal Pol III promoters) typical of canonical eukaryotic tRNAs and can thus be expressed in the same way as endogenous tRNAs.^14,46^ Even when using external Pol III promoters that do not rely on A- and B-box (such as U6 or H1 promoters), *Bst*Yam gives in mammalian cell hosts higher expression yields of ncAA-protein compared to the *Ec*tRNA^Tyr^.^51^ Most recently, a virus-assisted system that enables selection in mammalian cells has been reported and applied to the evolution of enhanced tRNAs.^18,19^ However, selection in mammalian cells is still not generally applied.

Upon the discovery of the AA Pyrrolysine (Pyl) **152** and the genes coding for its dedicated PylRS/tRNA^Pyl^ pair, which naturally enables its incorporation into proteins in response to an amber codon in *Methanosarcina* species of archaebacteria, it was only logical to transplant this pair to other organisms for ncAA incorporation via amber suppression.^52,53^ An exquisite characteristic of the Pyl-system is that it is orthogonal in both bacteria and mammalian cells, so that AARS selected in bacteria can directly be shuttled to mammalian cells, without performing any steps in yeast. Soon after the first experiments in *E. coli*,^54−56^ a set of Lys derivatives were incorporated into proteins in both bacteria and mammalian cells by the *Methanosarcina mazei* and *barkeri* PylRS/tRNA^Pyl^ pairs, which feature a conserved binding pocket.^57−61^ Later, modification of the *Mb*-tRNA^Pyl^ paired to the *Mb*PylRS was performed to make the system orthogonal in yeast.^62^ A baculovirus-based method for GCE using the Pyl-system has also been developed for high-yield expression of eukaryotic proteins in insect cells, and it was applied for multiprotein engineering.^63,64^ However, GCE in insect cells has not found widespread application yet.

A further very convenient characteristic of the PylRS is that it features a large AA binding pocket capable of accommodating large hydrophobic moieties, whereby the key for the recognition of the substrate is a crucial interaction between the side-chain carbonyl of the AA and an Asn residue suitably placed in the enzyme. As a consequence, the PylRS is quite tolerant toward the nature of the acyl group attached to the ε-amine, provided that the length of the aliphatic chain and the amide bond is conserved.^58^ While the mutation of a single residue (Y384F in *Mm*PylRS) produced enzymes with enhanced general activity toward Pyl-like ncAAs, a single additional mutation enlarging the binding pocket behind the amide lock (Y306A in *Mm*PylRS) allowed incorporation of ncAAs carrying bulkier side chains. This flexibility of the PylRS has allowed the incorporation of many different ncAAs into proteins using the natural enzyme or its variants carrying minimal modifications in the binding pocket, while many other variants have been selected from libraries designed for desired substrates. Later, new variants of the PylRS have been evolved to also incorporate structurally divergent AAs that do not feature an amide group at the ε position, such as Phe and His analogues,^65,66^ as well as a variety of AAs with short aliphatic side chains.^35^ Overall, so far more than 100 ncAAs have been genetically encoded by the Pyl system.

Another useful feature of the PylRS system is that the synthetase does not recognize the anticodon of its tRNA. This means that modification of the triplet into another nonsense codon is readily feasible, yielding ochre or opal suppressor tRNAs, or tRNAs complementary to quadruplet codons. Double stop-codon suppression in the same protein was demonstrated by pairing the *Mj*TyrRS/tRNA_CUA_ and *Mb*PylRS/tRNA_UUA_ pairs for amber and ochre suppression in *E. coli*(67) and by pairing the *Ec*TyrRS/tRNA_CUA_ and *Mb*PylRS/tRNA_UCA_ for amber and opal suppression in a membrane protein in mammalian cells.^17^

Double ncAA incorporation has also been demonstrated using two mutually orthogonal engineered PylRS systems, in particular by applying the PylRS from *Methanomethylophilus alvus*,^68−71^ which has been discovered more recently. This variant is smaller than *Mm*PylRS, as it naturally lacks the N-terminal domain, but it shares a homologous active site.^68^ The *Ma*PylRS was shown to be orthogonal in bacteria and mammalian cells, as well as to the *Mm*PylRS, providing an alternative for dual incorporation of ncAAs. The combination of *Ma*PylRS and *Mm*PylRS, together with the development of mutually orthogonal variants of tRNA^Pyl^, afforded the incorporation of two ncAAs (TCO*K **143** and AlkK **115**) through amber and ochre suppression in the same protein in mammalian cells.^72^ Similarly, double ncAA incorporation has also been achieved with *Ma*PylRS and *Mm*PylRS by combining the amber codon with a quadruplet codon in bacteria.^71^ Triple incorporation of ncAAs has been reported in bacteria by combining amber stop codon suppression and two quadruplet codons while using three different variants of PylRS (*Mm*PylRS and class A and B ΔNPylRS).^73^ Additionally, the strategy was optimized to incorporate four ncAAs (pIodoF **36**, AlocK **122**, MeH **164** and Z-K **184**) into a single protein under four quadruplet codons.^74^ In mammalian cells, a critical point for multiple ncAA incorporation is the efficient delivery of all necessary genes. A viral system derived from pseudotyped baculovirus has been adapted for GCE in mammalian cells to overcome these problems,^75,76^ but it has not found broad application.

Recently, an innovative approach for the simultaneous incorporation of multiple different ncAAs in eukaryotes, albeit in distinct proteins, has been presented. The method makes use of film-like artificial organelles targeted to different cellular membranes, but its application has not yet gone beyond the expression of soluble cytosolic proteins.^77^

Besides the bulk of experiments involving incorporation of ncAAs in cultured cells, GCE has been achieved also in multicellular organisms and in animals. *C. elegans* is the first multicellular animal that was modified in order to expand its genetic code to 21 AAs, by introducing the *Mm*PylRS/tRNA_CUA_ pair for incorporation of Boc-Lys (Boc-K **183**) and AlkK **115**.^78,79^ The choice of *C. elegans* was due to the extensive knowledge on its development, the fact that its genome is sequenced, and the fact that amber codon suppression was not seriously detrimental. After GCE expansion in *Drosophila melanogaster* and *Arabidopsis thaliana*,^80,81^ genetic code expansion of living vertebrates was achieved in the brain of live mice^82^ with the aid of an adenoviral construct introducing the PylRS/tRNA pair in the system, as well as in mice and zebrafish by integration of the gene coding for the AziRS/tRNA pair, which incorporates pAziF (**32**) at the amber stop codon.^83^ Additionally, the availability of CRISPR/CAS9 technology made possible the generation of transgenic mice expressing the gene for PylRS/tRNA, which allowed the assessment of the long-term safety of GCE in mammals.^84^

### 2.2 The Choice of ncAA (What ncAAs Can We Incorporate?)

**Figure 2:**
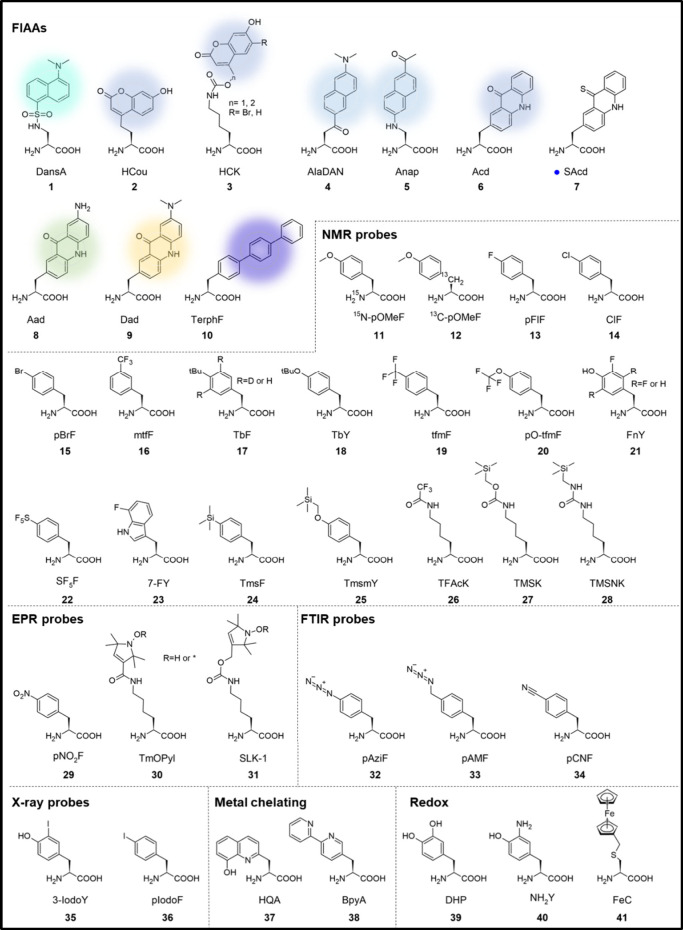

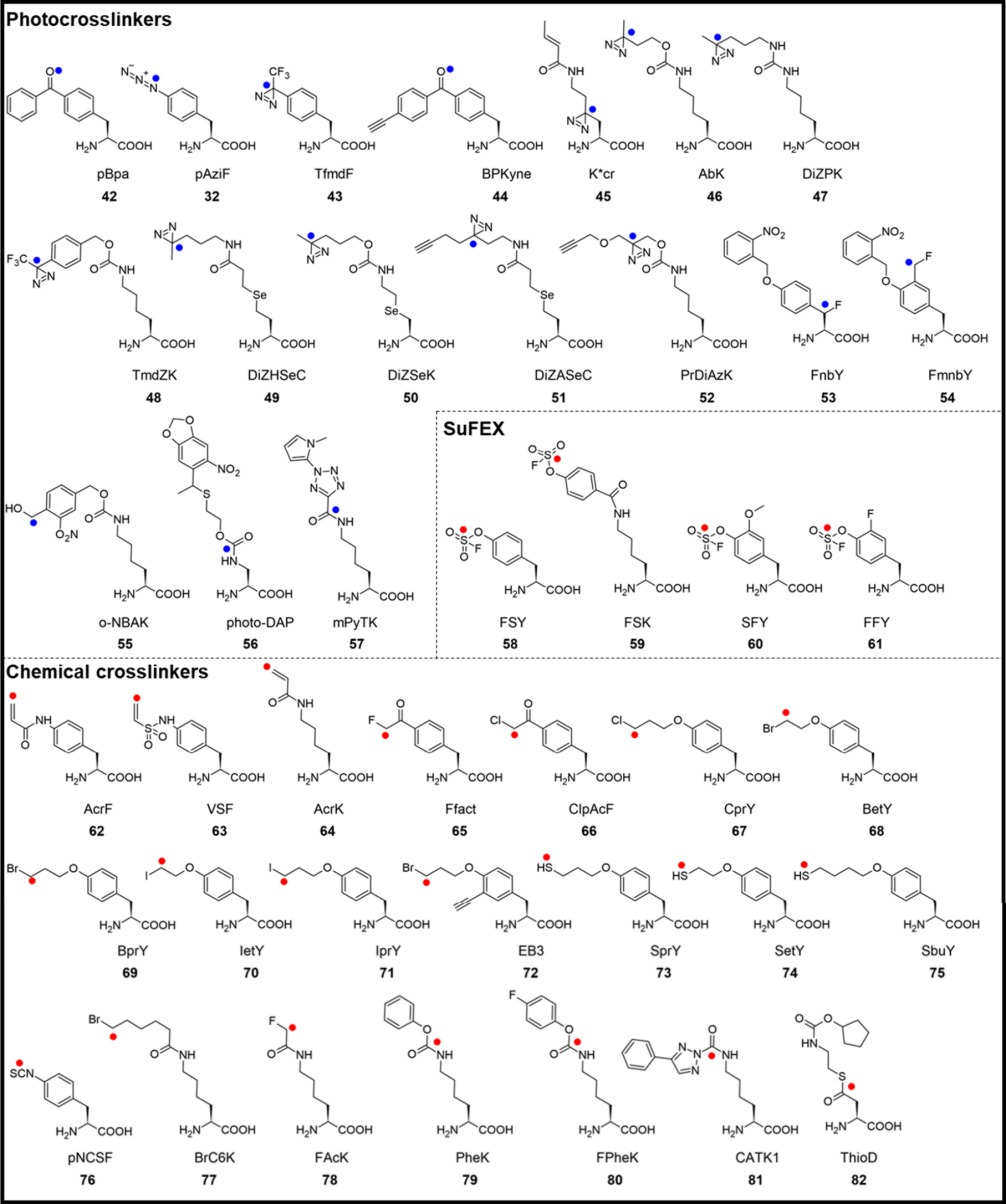

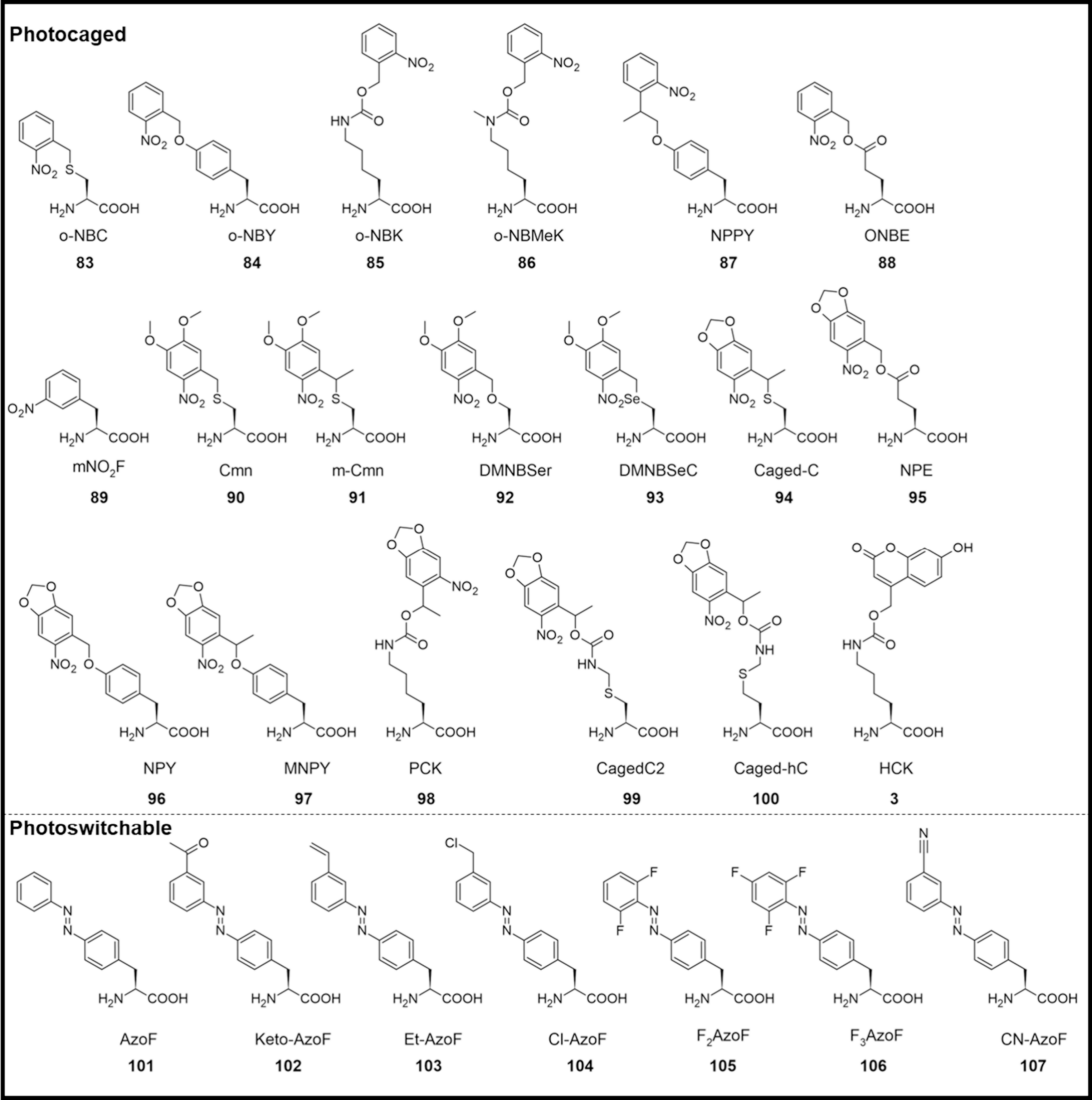

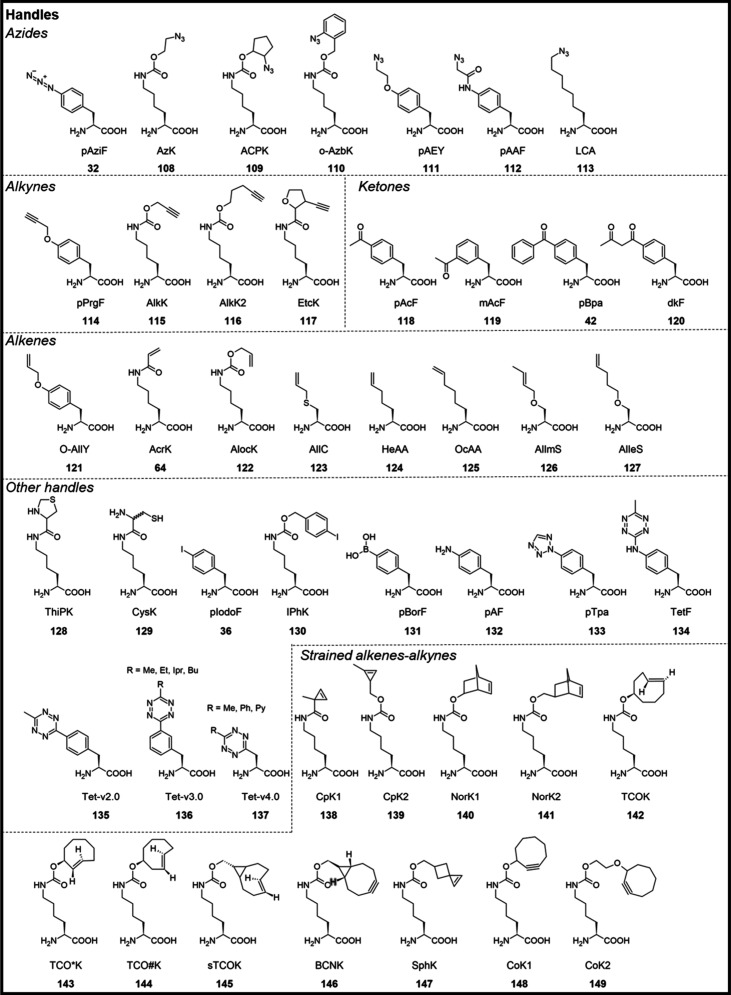

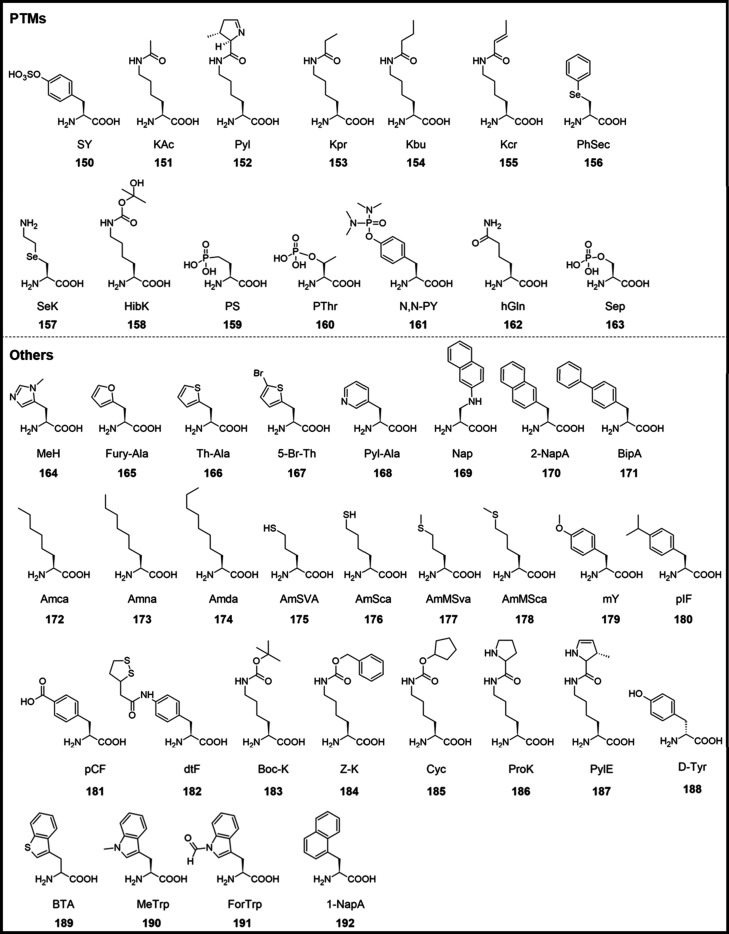
NcAAs incorporated into proteins using GCE technology. **1**–**10**: Fluorescent amino acids; the color is indicative of the respective fluorescence emission wavelength. **7** is fluorogenic and is converted to **6** after photoactivation. **11**–**28**: Amino acids for NMR spectroscopy. **29**–**31**: Amino acids carrying an unpaired electron for EPR spectroscopy. **32**–**34**: Amino acids with triple bonds suitable for FT-IR spectroscopy. **35**–**36**: Amino acids with an iodine atom useful for X-ray scattering techniques. **37**–**38**: Metal-chelating amino acids. **39**–**41**: Amino acids with redox properties. NcAAs incorporated into proteins using GCE technology. **42**–**57** (and **32**): Amino acids available for photo-cross-linking. The cross-linking position is highlighted by a blue dot. **58**–**61**: Amino acids for SuFEX chemistry. Electrophilic positions are highlighted with a red dot. **62**–**82**: Amino acids available for chemical cross-linking. Electrophilic positions are highlighted with a red dot. NcAAs incorporated into proteins using GCE technology. **83**–**100** (and **3**): Photocaged amino acids. Wavelengths for decaging vary between 365 and 405 nm. **101**–**107**: Photoswitchable amino acids. Wavelengths for photoswitching are ∼335–370 nm (*trans* to *cis*) and 420–450 nm (*cis* to *trans*). NcAAs incorporated into proteins using GCE technology. **108**–**113** (and **33**): Azides. **114**–**117**: Alkynes. **118**–**120** (and **42**): Ketones. **121**–**127** (and **64**): Alkenes. **128**–**137** (and **36**): Other handles. **138**–**149**: Strained alkenes and alkynes. NcAAs incorporated into proteins using GCE technology. **150**–**163**: Amino acids that mimic post-translational modifications (PTMs). **164**–**192**: Other amino acids that have been incorporated into proteins.

The palette of ncAAs that can be incorporated into expressed proteins using GCE comprises over 200 distinct entities and spans a wide range of functional moieties.^7,37,85−87^ In addition to the basic requirement for orthogonality, these ncAAs need to be stable in the cellular environment, a condition that is not always easy to achieve when introducing AAs with reactive functional groups. Although some attempts have been made to generate organisms that not only incorporate but also synthesize intracellularly the desired ncAA themselves,^88−90^ most GCE strategies use ncAAs that are supplied to the host organisms externally. In this context, membrane permeability sometimes becomes an issue for ncAAs that differ significantly from endogenous ones. Not all ncAAs are recognized by endogenous AA transporters, or they are simply not very lipophilic. In these cases, esterification of the carboxyl group has been shown to improve the uptake of bulky derivatives, since it increases the fraction of the neutral form of the modified ncAA.^91,92^ Shown in Figure 2 are some of the ncAAs that can be incorporated into proteins by GCE, roughly grouped by their function. Table 1 provides further information about these ncAAs.

**Table 1:** List of ncAAs

**#**	**Common Name**	**IUPAC**	**Abbrev**	**Year**	**Organism**^a^	**AARS**^b^	**Notes**	**Ref**^c^
**1**	l-Dansylalanine	(2S)-2-amino-3-[[5-(dimethylamino)naphthalen-1-yl]sulfonylamino]propanoic acid	DansA	2006	Yeast and Mammalian	*Ec*LeuRS		(49)
**2**	l-7-hydroxycoumarin-4-yl-ethylglycine	(2S)-2-amino-4-(7-hydroxy-2-oxochromen-4-yl)butanoic acid	HCou	2006	*E. coli*	*Mj*TyrRS		(98)
**3**	6-R-7-hydroxycoumarinmethyl-l-lysine	(2S)-amino-6-[(7-hydroxy-2-oxochromen-4-yl)methoxycarbonylamino]hexanoic acid	HCK	2014	*E. coli*, Yeast and Mammalian	*Mb*PylRS		(99)
**4**	6-dimethylamino-2-acylnaphthalene-l-alanine	(2S)-2-amino-4-[6-(dimethylamino)naphthalen-2-yl]-4-oxobutanoic acid	AlaDAN	2002	-	-	*Only introduced in peptides*	(100)
**5**	3-(6-acetylnaphthalen-2-ylamino)-2-aminopropanoic acid	3-[(6-acetylnaphthalen-2-yl)amino]-2-aminopropanoic acid	Anap	2009	Yeast and Mammalian	*Ec*LeuRS		(101)
**6**	Acridon-2-yl-l-alanine	(2S)-2-amino-3-(9-oxo-9,10-dihydroacridin-2-yl)propanoic acid	Acd	2013	*E. coli* and Mammalian	*Mj*TyrRS/*Mb*PylRS		(102,103)
**7**	Thioacridon-2-yl-l-alanine	(2S)-2-amino-3-(9-sulfanylidene-9,10-dihydroacridin-2-yl)propanoic acid	Sacd	2021	*E. coli*	*Mj*TyrRS		(104)
**8**	7-aminoacridon-2-yl-l-alanine	(2S)-2-amino-3-(7-amino-9-oxo-9,10-dihydroacridin-2-yl)propanoic acid	Aad	2021	-	-	*Not incorporated yet*	(105)
**9**	7-(dimethylamino)acridon-2-yl-l-alanine	(2S)-2-amino-3-[7-(dimethylamino)-9-oxo-9,10-dihydroacridin-2-yl]propanoic acid	Dad	2021	-	-	*Not incorporated yet*	(105)
**10**	4-biphenyl-l-phenylalanine	(2S)-2-amino-3-[4-(4-phenylphenyl)phenyl]propanoic acid	TerphF	2015	*E. coli*	*Mj*TyrRS		(106)
**11**	^15^N-4*-*methoxy-l-phenylalanine	(2S)-2-^15^N-amino-3-(4-methoxyphenyl)propanoic acid	^15^N-*p*OMeF	2005	*E. coli*	*Mj*TyrRS		(107)
**12**	^13^C-4-methoxy-l-phenylalanine	(2S)-2-amino-3-^13^C- (4-methoxyphenyl)propanoic acid	^13^C-*p*OMeF	2008	*E. coli*	*Mj*TyrRS		(108)
**13**	*p*-fluoro-l-phenylalanine	(2S)-2-amino-3-(4-fluorophenyl)propanoic acid	*p*FlF	1998	*E. coli*	*Sc*PheRS	*E. coli Phe-auxotrophic strain*	(109)
**14**	*p*-chloro-l-phenylalanine	(2S)-2-amino-3-(4-chlorophenyl)propanoic acid	ClF	2020	*E. coli*, Yeast and Mammalian	*Mj*TyrRS/*Mm*PylRS		(110,111)
**15**	*p*-bromo-l-phenylalanine	(2S)-2-amino-3-(4-bromophenyl)propanoic acid	pBrF	2003	*E. coli*, Yeast and Mammalian	*Mj*TyrRS/*Ec*TyrRS		(75,112)
**16**	*m*-trifluoromethyl-l-phenylalanine	(2S)-2-amino-3-[3-(trifluoromethyl)phenyl]propanoic acid	mtfF	2021	*E. coli*, Yeast and Mammalian	*Mb*PylRS		(113)
**17**	*p*-*tert*-butyl-l-phenylalanine	(2S)-2-amino-3-(4-*tert*-butylphenyl)propanoic acid	TbF	2018	*E. coli*	*Mj*TyrRS		(114)
**18**	*p*-*tert*-butyl-l-tyrosine	(2S)-2-amino-3-[4-[(2-methylpropan-2-yl)oxy]phenyl]propanoic acid	TbY	2015	*E. coli*, Yeast and Mammalian	*Mj*TyrRS/ *Mb*PylRS		(115,116)
**19**	*p-*trifluoromethyl-l-phenylalanine	(2S)-2-amino-3-[4-(trifluoromethyl)phenyl]propanoic acid	tfmF	2006	*E. coli*	*Mj*TyrRS		(117)
**20**	*p-*trifluoromethoxy-l-phenylalanine	(2S)-2-amino-3-[4-(trifluoromethoxy)phenyl]propanoic acid	pO-tfmF	2008	*E. coli*	*Mj*TyrRS		(118)
**21**	n-fluoro-l-tyrosine	(2S)-2-amino-3-(n,3,n-fluoro-4-hydroxyphenyl)propanoic acid	FnY	2011	*E. coli*	*Mj*TyrRS		(119)
**22**	*p*-pentafluorosulfanyl-l-phenylalanine	(2S)-amino-3-[4-(pentafluoro-λ6-sulfanyl)phenyl]propanoic acid	SF_5_F	2020	*E. coli*	*Mj*TyrRS		(110)
**23**	7-fluoro-l-tryptophan	(2S)-amino-3-(7-fluoro-1H-indol-3-yl)propanoic acid	7-FY	2022	*E. coli*, Yeast and Mammalian	*Mb*PylRS		(120)
**24**	*p*-trimethylsilyl-l-phenylalanine	(2S)-amino-3-(4-trimethylsilylphenyl)propanoic acid	TmsF	2008	*E. coli*	*Mj*TyrRS		(114)
**25**	*p*-(trimethylsilyl)methoxy-l-phenylalanine	(2S)-amino-3-(4-trimethylsilylmethoxy phenyl)propanoic acid	TmsmY	2008	*E. coli*	*Mj*TyrRS		(114)
**26**	N^ε^-trifluoroacetyl-l-lysine	(2S)-2-amino-6-[(2,2,2-trifluoroacetyl)amino]hexanoic acid	TFAcK	2018	*E. coli*, Yeast and Mammalian	*Mb*PylRS		(121)
**27**	N^ε^-((Trimethylsilyl)methoxy)carbonyl-l-lysine	(2S)-2-amino-6-(trimethylsilylmethoxycarbonylamino)hexanoic acid	TMSK	2021	*E. coli*, Yeast and Mammalian	*Mb*PylRS		(122)
**28**	N^ε^-((trimethylsilyl)methyl)carbamoyl-l-lysine	(2S)-2-amino-6-(trimethylsilylmethylcarbamoylamino)hexanoic acid	TMSNK	2023	*E. coli*, Yeast and Mammalian	*Mb*PylRS		(123)
**29**	*p*-nitro-l-phenylalanine	(2S)-2-amino-3-(4-nitrophenyl)propanoic acid	pNO_2_F	2006	*E. coli*	*Mj*TyrRS		(124)
**30**	2,2,5,5-tetramethyl-pyrrolin-1-oxyl-l-pyrrolysine	(2S)-2-amino-6-[(1-hydroxy-2,2,5,5-tetramethyl-2,5-dihydro-1H-pyrrole-3-carbonyl)amino]hexanoic acid	TmOPyl	2014	-	*-*	*Not incorporated yet*	(94)
**31**	(S)-2-((tert-butoxycarbonyl)amino)-6-((((1-oxy2,2,5,5-tetramethylpyrroline-3-yl)methoxy)carbonyl)amino) hexanoic acid	(2S)-2-amino-6-[(1-hydroxy-2,2,5,5-tetramethylpyrrol-3-yl)methoxycarbonylamino]hexanoic acid	SLK-1	2014	*E. coli*, Yeast and Mammalian	*Mm*PylRS		(94)
**32**	*p*-azido-l-phenylalanine	(2S)-2-amino-3-(4-azidophenyl)propanoic acid	pAziF	2009	*E. coli*, Yeast, Mammalian	*Mj*TyrRS/*Ec*TyrRS		(40,125)
**33**	*p*-azidomethyl-l-phenylalanine	(2S)-2-amino-3-[4-(azidomethyl)phenyl]propanoic acid	*p*AMF	2013	*E. coli*	*Mj*TyrRS		(126)
**34**	*p*-cyano-l-phenylalanine	(2S)-2-amino-3-(4-cyanophenyl)propanoic acid	*p*CNF	2006	*E. coli*, Yeast, Mammalian	*Mj*TyrRS/*Ec*TyrRS		(75,127)
**35**	3-iodo-l-tyrosine	(2S)-2-amino-3-(4-hydroxy-3-iodophenyl)propanoic acid	3-IodoY	2002	*E. coli*	*Mj*TyrRS		(46)
**36**	*p*-iodo-l-phenylalanine	(2S)-2-amino-3-(4-iodophenyl)propanoic acid	*p*IodoF	2004	*E. coli*, Yeast and Mammalian	*Mj*TyrRS/*Ec*TyrRS		(128,129)
**37**	(8-hydroxyquinolin-3-yl)-l-alanine	(2S)-2-amino-3-(8-hydroxyquinolin-2-yl)propanoic acid	HQA	2008	*E. coli*	*Mj*TyrRS		(130)
**38**	bipyridyl-l-alanine	(2S)-2-amino-3-(6-pyridin-2-ylpyridin-3-yl)propanoic acid	BpyA	2007	*E. coli*	*Mj*TyrRS		(131)
**39**	3,4-dihydroxy-l-phenylalanine	(2S)-2-amino-3-(3,4-dihydroxyphenyl)propanoic acid	DHP	2003	*E. coli*	*Mj*TyrRS		(132)
**40**	3-amino-l-tyrosine	(2S)-2-amino-3-(4-hydroxy-3-aminophenyl)propanoic acid	NH_2_Y	2007	*E. coli*, Yeast and Mammalian	*Mj*TyrRS/*Ec*TyrRS		(133,134)
**41**	ferrocene-l-cysteine	(2R)-2-amino-3-(methyl bicyclopentadienyl iron(II)sulfanyl)propanoic acid	FeC	2007	Yeast	*Ec*LeuRS		(135)
**42**	*p*-benzoyl-l-phenylalanine	(2S)-2-amino-3-(4-benzoylphenyl)propanoic acid	pBpa	2002	*E. coli*, Yeast and Mammalian	*Mj*TyrRS/*Ec*TyrRS		(39,45)
**43**	4-(3-(trifluoromethyl)-3H-diazin-3-yl)-l-phenylalanine	(2S)-2-amino-3-[4-[3-(trifluoromethyl)diazirin-3-yl]phenyl]propanoic acid	TfmdF	2007	*E. coli*, Yeast and Mammalian	*Mj*TyrRS/*Ec*TyrRS		(136,137)
**44**	*p*-(4-ethynyl)benzoyl- l-phenylalanine	(2S)-2-amino-3-(4-(4-ethynylbenzoyl)phenyl)propanoic acid	BPKyne	2017	Yeast and Mammalian	*Ec*TyrRS		(138)
**45**	photocrotonyl- l-lysine	(2S)-2-amino-3-(3-{2-[(2E)-but-2-enamido]ethyl}-3H-diazirin-3-yl)propanoic acid	K^a^cr	2017	*E. coli*, Yeast and Mammalian	*Mm*PylRs		(139)
**46**	3-azibutyl-N-carbamoyl-l-lysine	(2S)-2-amino-6-({[2-(3-methyl-3H-diazirin-3-yl)ethoxy]carbonyl}amino)hexanoic acid	AbK	2010	*E. coli*, Yeast and Mammalian	*Mb*PylRS		(62)
**47**	N^ε^-(3-(3-methyl-3H-diazirine-3-yl)-propaminocarbonyl)-l-lysine	(2S)-2-amino-6-{[3-(3-methyl-3H-diazirin-3-yl)propyl]carbamamido}hexanoic acid	DiZPK	2011	*E. coli*, Yeast and Mammalian	*Mb*PylRS		(140)
**48**	N^ε^-[((4-(3-(trifluoromethyl)-3H-diazirin-3-yl)benzyl)oxy)carbonyl]-l-lysine	(2S)-2-amino-6-[[4-[3-(trifluoromethyl)diazirin-3-yl]phenyl]methoxycarbonylamino]hexanoic acid	TmdZK	2012	*E. coli*, Yeast and Mammalian	*Mm*PylRS		(141)
**49**	Se-(N -(3-(3-methyl-3H-diazirin-3-yl)propyl)propanamide)-3-yl-l-homoselenocysteine	(2S)-2-amino-4-({3-[(4-(3-methyl-3H-diazirin-3-yl)butyl)amino]-3-oxopropyl}selanyl)butanoic acid	DiZHSeC	2016	*E. coli*, Yeast and Mammalian	*Mb*PylRS		(142)
**50**	N^ε^-3-((3-methyl-3H-diazirine-3-yl))-propamino-carbonyl-γ-seleno-l-lysine	(2S)-2-amino-3-((2-(((3-(3-methyl-3H-diazirin-3-yl)propoxy)carbonyl)amino)ethyl)selanyl)propanoic acid	DiZSeK	2014	*E. coli*, Yeast and Mammalian	*Mb*PylRs		(143)
**51**	Se-(N-(2-(3-[but3-yn-1-yl]-3H-diazirin-3-yl) ethyl)propionamide)-3-yl-l-homoselenocysteine	(2S)-2-amino-4-({3-[(3-(3-butyn-4-yl-3H-diazirin-3-yl)propyl)amino]-3-oxopropyl}selanyl)butanoic acid	DiZASeC	2017	*E. coli*, Yeast and Mammalian	*Mb*PylRs		(144)
**52**	Propargyl-diazirine-l-lysine	(2S)-2-amino-6-[[3-(prop-2-ynoxymethyl)diazirin-3-yl]methoxycarbonylamino]hexanoic acid	PrDiAzK	2018	*E. coli*, Yeast and Mammalian	*Mm*PylRs		(145)
**53**	(2R)-2-amino-3-fluoro3-(4-((2-nitrobenzyl)oxy) phenyl)-propanoic acid	(2R)-2-amino-3-fluoro-3-{4-[(2-nitrophenyl)methoxy]phenyl}propanoic acid	FnbY	2019	*E. coli*, Yeast and Mammalian	*Mb*PylRS		(146)
**54**	o-2-nitrobenzyl-3-fluoromethyl- l-tyrosine	(2S)-2-amino-3-{3-(fluoromethyl)-4-[(2-nitrophenyl)methoxy]phenyl}propanoic acid	FmnbY	2020	*E. coli*, Yeast and Mammalian	*Ma*PylRS		(147)
**55**	o-nitrobenzyl-alcohol-l-lysine	(2S)-2-amino-6-[({[4-(hydroxymethyl)-3-nitrophenyl]methoxy}carbonyl)amino]hexanoic acid	o-NBAK	2019	*E. coli*, Yeast and Mammalian	*Mm*PylRs		(148)
**56**	photocaged 2,3-diaminopropionic acid	(2S)-2-amino-3-[2-[1-(6-nitro-1,3-benzodioxol-5-yl)ethylsulfanyl]ethoxycarbonylamino]propanoic acid	photo-DAP	2019	*E. coli*, Yeast and Mammalian	*Mb*PylRS		(149)
**57**	*N*-methylpyrroletetrazole-l-lysine	(2S)-2-amino-6-{[2-(1-methyl-1H-pyrrol-2-yl)-2H-tetrazole-5-carbonyl]amino}hexanoic acid	mPyTK	2017	*E. coli*, Yeast and Mammalian	*Mm*PylRs		(150)
**58**	fluorosulfate-l-tyrosine	(2S)-2-amino-3-{4-[(fluorosulfonyl)oxy]phenyl}propanoic acid	FSY	2018	*E. coli*, Yeast and Mammalian	*Mm*PylRs		(151)
**59**	fluorosulfonyloxybenzoyl-l-lysine	(2S)-2-amino-6-{4-[(fluorosulfonyl)oxy]benzamido}hexanoic acid	FSK	2021	*E. coli*, Yeast and Mammalian	*Ma*PylRS		(152)
**60**	*m-*fluorosulfate-O-methyl-l-tyrosine	(2S)-2-amino-3-{4-[(fluorosulfonyl)oxy]-3-methoxyphenyl}propanoic acid	SFY	2023	*E. coli*, Yeast and Mammalian	*Mm*PylRS		(153)
**61**	fluorosulfate-3-fluoro-l-tyrosine	(2S)-2-amino-3-{3-fluoro-4-[(fluorosulfonyl)oxy]phenyl}propanoic acid	FFY	2022	*E. coli*, Yeast and Mammalian	*Mm*PylRs		(154)
**62**	4-acrylamido-l-phenylalanine	(2S)-2-amino-3-[4-(prop-2-enamido)phenyl]propanoic acid	AcrF	2014	*E. coli*	*Mj*TyrRS		(155)
**63**	*p-*vinylsulfonamido-l-phenylalanine	(2S)-2-amino-3-[4-(ethenesulfonamido)phenyl]propanoic acid	VSF	2014	*E. coli*	*Mj*TyrRS		(155)
**64**	N^ε^-acryloyl-l-lysine	(2S)-2-amino-6-(prop-2-enamido)hexanoic acid	AcrK	2013	*E. coli*, Yeast and Mammalian	*Mb*PylRS		(80)
**65**	4–2′-fluoroacetyl-l-phenylalanine	(2S)-2-amino-3-[4-(fluoroacetyl)phenyl]propanoic acid	Ffact	2013	*E. coli and Mammalian*	*Mj*TyrRS, *Ec*TyrRS		(156)
**66**	4–2′-chloroacetyl-l-phenylalanine	(2S)-2-amino-3-[4-(chloroacetyl)phenyl]propanoic acid	ClpAcF	2018	*E. coli and Mammalian*	*Mj*TyrRS, *Ec*TyrRS		(157)
**67**	O-(3-chloropropyl)-l-tyrosine	(2S)-2-amino-3-[4-(3-chloropropoxy)phenyl]propanoic acid	CprY	2014	*E. coli*, Yeast and Mammalian	*Mm*PylRS		(158)
**68**	O-(3-bromoethyl)-l-tyrosine	(2S)-2-amino-3-[4-(2-bromoethoxy)phenyl]propanoic acid	BetY	2014	*E. coli*, Yeast and Mammalian	*Mm*PylRS		(158)
**69**	O-(3-bromopropyl)-l-tyrosine	(2S)-2-amino-3-[4-(3-bromopropoxy)phenyl]propanoic acid	BprY	2014	*E. coli*, Yeast and Mammalian	*Mm*PylRS		(158)
**70**	O-(3-iodoethyl)-l-tyrosine	(2S)-2-amino-3-[4-(2-iodoethoxy)phenyl]propanoic acid	IetY	2014	*E. coli*, Yeast and Mammalian	*Mm*PylRS		(158)
**71**	O-(3-iodopropyl)-l-tyrosine	(2S)-2-amino-3-[4-(3-iodopropoxy)phenyl]propanoic acid	IprY	2014	*E. coli*, Yeast and Mammalian	*Mm*PylRS		(158)
**72**	O-(3-bromoethyl-4-acetylene)-l-tyrosine	(2S)-2-amino-3-[4-(3-bromopropoxy)-3-ethynylphenyl]propanoic acid	EB3	2017	*E. coli*, Yeast and Mammalian	*Mm*PylRS		(159)
**73**	O-(3-mercaptopropyl)-l-tyrosine	(2S)-2-amino-3-[4-(3-sulfanylpropoxy)phenyl]propanoic acid	SprY	2016	*E. coli*, Yeast and Mammalian	*Mm*PylRS		(160)
**74**	O-(2-mercaptoethyl)-l-tyrosine	(2S)-2-amino-3-[4-(2-sulfanylethoxy)phenyl]propanoic acid	SetY	2016	*E. coli*, Yeast and Mammalian	*Mm*PylRS		(160)
**75**	O-(4-mercaptobutyl)-l-tyrosine	(2S)-2-amino-3-[4-(4-sulfanylbutoxy)phenyl]propanoic acid	SbuY	2016	*E. coli*, Yeast and Mammalian	*Mm*PylRS		(160)
**76**	*p*-isothiocyanate-l-phenylalanine	(2S)-2-amino-3-[4-(thiocyanato)phenyl]propanoic acid	*p*NCSF	2016	*E. coli*	*Mj*TyrRS		(161)
**77**	(S)-2-amino-6-(6-bromohexanamido)-hexanoic acid	(2S)-2-amino-6-(6-bromohexanamido)hexanoic acid	BrC6K	2014	*E. coli*, Yeast and Mammalian	*Mm*PylRS		(162)
**78**	*N*^ε^-fluoroacetyl-l-lysine	(2S)-2-amino-6-(2-fluoroacetamido)hexanoic acid	FAcK	2016	*E. coli*, Yeast and Mammalian	*Mm*PylRS		(163)
**79**	phenylcarbamate-l-lysine	(2S)-2-amino-6-[(phenoxycarbonyl)amino]hexanoic acid	PheK	2017	*E. coli*, Yeast and Mammalian	*Mb*PylRS		(164)
**80**	fluorophenylcarbamate-l-lysine	(2S)-2-amino-6-{[(4-fluorophenoxy)carbonyl]amino}hexanoic acid	FPheK	2017	*E. coli*, Yeast and Mammalian	*Mb*PylRS		(164)
**81**	N^2^-carboxy-4-phenyl-1,2,3-triazole-l-lysine	(2S)-2-amino-6-[(4-phenyl-2H-1,2,3-triazole-2-carbonyl)amino]hexanoic acid	CATK1	2022	*E. coli*, Yeast and Mammalian	*Mm*PylRS		(165)
**82**	thio-aspartate-l-lysine	(2S)-2-amino-4-[(2-{[(cyclopentyloxy)carbonyl]amino}ethyl)sulfanyl]-4-oxobutanoic acid	ThioD	2018	*E. coli*, Yeast and Mammalian	*Mb*PylRS		(166)
**83**	o-nitrobenzyl-l-cysteine	(2R)-2-amino-3-{[(2-nitrophenyl)methyl]sulfanyl}propanoic acid	oNBC	2004	Yeast and Mammalian	*Ec*LeuRS	*Also with*^*13*^*C for NMR*	(48)
**84**	o-nitrobenzyl-l-tyrosine	(2S)-2-amino-3-{4-[(2-nitrophenyl)methoxy]phenyl}propanoic acid	oNBY	2006	*E. coli*	*Mj*TyrRS	Also with ^15^N for NMR	(167)
**85**	o-nitrobenzyloxycarbonyl-N^ε^-l-lysine	(2S)-2-amino-6-({[(2-nitrophenyl)methoxy]carbonyl}amino)hexanoic acid	oNBK	2009	*E. coli*, Yeast and Mammalian	*Mm*PylRS		(168)
**86**	N^ε^ -o-nitrobenzyloxycarbonyl-l-methyllysine	(2S)-2-amino-6-(methyl{[(2-nitrophenyl)methoxy]carbonyl}amino)hexanoic acid	oNBMeK	2010	*E. coli*, Yeast and Mammalian	*Mb*PylRS		(169)
**87**	nitrophenylpropyl-l-tyrosine	(2S)-2-amino-3-{4-[2-(2-nitrophenyl)propoxy]phenyl}propanoic acid	NPPY	2017	*E. coli*, Yeast and Mammalian	*Mb*PylRS		(170)
**88**	O-nitrobenzyl-oxycarbonyl-N^ε^-l-glutamate	(2S)-2-amino-5-[(2-nitrophenyl)methoxy]-5-oxopentanoic acid	ONBE	2023	*E. coli*, Yeast and Mammalian	*Mb*PylRS		(171)
**89**	3-nitro-l-phenylalanine	(2S)-2-amino-3-(3-nitrophenyl)propanoic acid	*m*NO_2_F	2009	*E. coli*, Yeast and Mammalian	*Mj*TyrRS/*Mb*PylRS		(172,173)
**90**	4,5-dimethoxy-2-nitrobenzyl-l-cysteine	(2R)-2-amino-3-{[(4,5-dimethoxy-2-nitrophenyl)methyl]sulfanyl}propanoic acid	Cmn	2013	Mammalian, Yeast	*Ec*LeuRS		(174)
**91**	Metyl-4,5-dimethoxy-2-nitrobenzyl-l-cysteine	(2R)-2-amino-3-{[1-(4,5-dimethoxy-2-nitrophenyl)ethyl]sulfanyl}propanoic acid	m*-*Cmn	2015	Mammalian, Yeast	*Ec*LeuRS		(175)
**92**	4,5-dimethoxy-2-nitrobenzyl-l-serine	(2S)-2-amino-3-[(4,5-dimethoxy-2-nitrophenyl)methoxy]propanoic acid	DMNB-Ser	2007	Yeast, Mammalian	*Ec*LeuRS		(176)
**93**	4,5-dimethoxy-2-nitrobenzyl-l-selenocysteine	(2S)-2-amino-3-[(4,5-dimethoxy-2-nitrophenyl)methylselanyl]propanoic acid	DMNBSeC	2015	Yeast, Mammalian	*Ec*LeuRS		(177)
**94**	S-[(R,S)-1-(4′,5′-(methylenedioxy)-2′-nitrophenyl)ethyl]-l-cysteine	(2R)-2-amino-3-{[1-(6-nitro-2H-1,3-benzodioxol-5-yl)ethyl]sulfanyl}propanoic acid	Caged-C	2014	*E. coli*, Yeast and Mammalian	*Mb*PylRS		(178)
**95**	Nitropiperonyl-l-glutamate	(2S)-2-amino-5-[(6-nitro-2H-1,3-benzodioxol-5-yl)methoxy]-5-oxopentanoic acid	NPE	2023	*E. coli*, Yeast and Mammalian	*Mb*PylRS		(171)
**96**	Nitropiperonyl-l-tyrosine	(2S)-2-amino-3-{4-[(6-nitro-2H-1,3-benzodioxol-5-yl)methoxy]phenyl}propanoic acid	NPY	2017	*E. coli*, Yeast and Mammalian	*Mb*PylRS		(170)
**97**	(Methyl)nitropiperonyl-l-tyrosine	(2S)-2-amino-3-{4-[1-(6-nitro-2H-1,3-benzodioxol-5-yl)ethoxy]phenyl}propanoic acid	MNPY	2017	*E. coli*, Yeast and Mammalian	*Mb*PylRS		(170)
**98**	(Methyl)nitropiperonyl-l-lysine	(2S)-2-amino-6-({[1-(6-nitro-2H-1,3-benzodioxol-5-yl)ethoxy]carbonyl}amino)hexanoic acid	PCK	2010	*E. coli*, Yeast and Mammalian	*Mb*PylRS		(179)
**99**	(2R)-2-amino-3-(((((1-(6-nitrobenzo[d][1,3]dioxol-5-yl)ethoxy)carbonyl)amino)methyl)thio)propanoic acid	(2R)-2-amino-3-{[({[1-(6-nitro-2H-1,3-benzodioxol-5-yl)ethoxy]carbonyl}amino)methyl]sulfanyl}propanoic acid	Caged-C2	2014	*E. coli*, Yeast and Mammalian	*Mb*PylRS		(180)
**100**	2-amino-4-(((((1-(6-nitrobenzo[d][1,3]dioxol-5-yl)ethoxy)carbonyl)amino)methyl)thio) butanoic acid	(2S)-2-amino-4-{[({[1-(6-nitro-2H-1,3-benzodioxol-5-yl)ethoxy]carbonyl}amino)methyl]sulfanyl}butanoic acid	Caged-hC	2014	*E. coli*, Yeast and Mammalian	*Mb*PylRS		(180)
**101**	4-phenylazo-l-phenylalanine	(2S)-2-amino-3-{4-[(E)-phenyldiazenyl]phenyl}propanoic acid	AzoF	2006	*E. coli*	*Mj*TyrRS		(181)
**102**	4-(3-keto)phenylazo-l-phenylalanine	(2S)-3-{4-[(E)-(3-acetylphenyl)diazenyl]phenyl}-2-aminopropanoic acid	Keto-AzoF	2014	*E. coli*, Yeast and Mammalian	*Mm*PylRS		(182)
**103**	4-(3-etylen)phenylazo- l-phenylalanine	(2S)-2-amino-3-{4-[(E)-(3-ethenylphenyl)diazenyl]phenyl}propanoic acid	Et-AzoF	2014	*E. coli*, Yeast and Mammalian	*Mm*PylRS		(182)
**104**	4-(3-chloro)phenylazo-l-phenylalanine	(2S)-2-amino-3-(4-{(E)-[3-(chloromethyl)phenyl]diazenyl}phenyl)propanoic acid	Cl-AzoF	2014	*E. coli*, Yeast and Mammalian	*Mm*PylRS		(182)
**105**	4-(2,6-difluoro)phenylazo-l-phenylalanine	(2S)-2-amino-3-{4-[(E)-(2,6-difluorophenyl)diazenyl]phenyl}propanoic acid	F_2_AzoF	2015	*E. coli*, Yeast and Mammalian	*Mm*PylRS		(183)
**106**	4-(2,4,6-trifluoro)phenylazo-l-phenylalanine	(2S)-2-amino-3-{4-[(E)-(2,4,6-trifluorophenyl)diazenyl]phenyl}propanoic acid	F_3_AzoF	2015	*E. coli*, Yeast and Mammalian	*Mm*PylRS		(183)
**107**	4-(3-cyano)phenylazo-l-phenylalanine	(2S)-2-amino-3-{4-[(E)-(3-cyanophenyl)diazenyl]phenyl}propanoic acid	CN-AzoF	2015	*E. coli*, Yeast and Mammalian	*Mm*PylRS		(183)
**108**	(S)-2-amino-6-((2-azidoethoxy)carbonylamino)hexanoic acid	(2S)-2-amino-6-{[(2-azidoethoxy)carbonyl]amino}hexanoic acid	AzK	2009	*E. coli*, Yeast and Mammalian	*Mb*PylRS		(61)
**109**	azido-cyclopentene-l-lysine	(2S)-2-amino-6-({[(2-azidocyclopentyl)oxy]carbonyl}amino)hexanoic acid	ACPK	2011	*E. coli*, Yeast and Mammalian	*Mb*PylRS		(184)
**110**	N^ε^-(oazidobenzyloxycarbonyl)-l-lysine	(2S)-2-amino-6-({[(2-azidophenyl)methoxy]carbonyl}amino)hexanoic acid	o-AzbK	2008	*E. coli*, Yeast and Mammalian	*Mm*PylRS		(58)
**111**	4-azidoethyl-l-tyrosine	(2S)-2-amino-3-[4-(2-azidoethoxy)phenyl]propanoic acid	*p*AEY	2022	Yeast and Mammalian	*Ec*TyrRS		(185)
**112**	4-azidoacetamido-l-phenylalanine	(2S)-2-amino-3-[4-(2-azidoacetamido)phenyl]propanoic acid	*p*AAF	2022	Yeast and Mammalian	*Ec*TyrRS		(185)
**113**	(2S)-2-amino-9-azidononanoic acid	(2S)-2-amino-9-azidononanoic acid	LCA	2023	Yeast and Mammalian	*EcLeuR*S		(186)
**114**	*p*-propargyloxy-l-phenylalanine	(2S)-2-amino-3-{4-[(prop-2-yn-1-yl)oxy]phenyl}propanoic acid	*p*PrgF	2003	*E. coli*, Yeast and Mammalian	MjTyrRS/*EcT*yrRS		(125,187)
**115**	N^ε^-[(2-propynyloxy)carbonyl]-l-lysine	(2S)-2-amino-6-({[(prop-2-yn-1-yl)oxy]carbonyl}amino)hexanoic acid	AlkK	2009	*E. coli*, Yeast and Mammalian	*Mb*PylRS		(188)
**116**	N^ε^-[(4-pentinyloxy)carbonyl]-l-lysine	(2S)-2-amino-6-({[(pent-4-yn-1-yl)oxy]carbonyl}amino)hexanoic acid	AlkK2	2013	*E. coli*, Yeast and Mammalian	*Mb*PylRS		(189)
**117**	N^ε^-(3-ethynyltetrahydrofuran-2-(carbonyl)-l-lysine	(2S)-2-amino-6-[(3-ethynyloxolane-2-carbonyl)amino]hexanoic acid	EtcK	2009	*E. coli*, Yeast and Mammalian	*Mm*PylRS		(60)
**118**	*p*-acetyl-l-phenylalanine	(2S)-3-(4-acetylphenyl)-2-aminopropanoic acid	pAcF	2003	*E. coli*	*Mj*TyrRS/EcTyrlRS		(10,75)
**119**	*m*-acetyl-l-phenylalanine	(2S)-3-(3-acetylphenyl)-2-aminopropanoic acid	mAcF	2003	*E. coli*	*Mj*TyrRS		(190)
**120**	Diketo-l-phenylalanine	(2S)-2-amino-3-[4-(3-oxobutanoyl)phenyl]propanoic acid	dkF	2006	*E. coli*	*Mj*TyrRS		(191)
**121**	O-allyl-l-tyrosine	(2S)-2-amino-3-{4-[(prop-2-en-1-yl)oxy]phenyl}propanoic acid	o-AllY	2002	*E. coli*	*Mj*TyrRS		(192)
**122**	N^ε^-(o-azidobenzyloxycarbonyl)-l-lysine	(2S)-2-amino-6-({[(prop-2-en-1-yl)oxy]carbonyl}amino)hexanoic acid	AlocK	2008	*E. coli*, Yeast and Mammalian	*Mm*PylRS		(58)
**123**	Allyl-sulphoxy-l-cysteine	(2R)-2-amino-3-(prop-2-ene-1-sulfinyl)propanoic acid	AllC	2010	Yeast	*Ec*LeuRS/*Mb*PylRS		(116,193)
**124**	α-amino-hepten-carboxylic acid	(2S)-2-aminohept-6-enoic acid	HeAA	2010	Yeast	*Ec*LeuRS		(193)
**125**	α-amino-octene-carboxylic acid	(2S)-2-aminooct-7-enoic acid	OcAA	2010	Yeast	*Ec*LeuRS		(193)
**126**	Allylmethyl-l-serine	(2S)-2-amino-3-{[(2E)-but-2-en-1-yl]oxy}propanoic acid	AllmS	2010	Yeast	*Ec*LeuRS		(193)
**127**	Allylethyl-l-serine	(2S)-2-amino-3-[(pent-4-en-1-yl)oxy]propanoic acid	AlleS	2010	Yeast	*Ec*LeuRS		(193)
**128**	N^ε^-L-thiaprolyl- l-lysine	2S)-2-amino-6-[(1,3-thiazolidine-4-carbonyl)amino]hexanoic acid	ThiPK	2011	*E. coli*, Yeast and Mammalian	*Mb*PylRS		(194)
**129**	N^ε^-l-cysteinyl-l-lysine	(2S)-2-amino-6-(2-amino-3-sulfanylpropanamido)hexanoic acid	CysK	2009	*E. coli*, Yeast and Mammalian	*Mm*PylRS		(195)
**130**	Iodophenyl-l-lysine	(2S)-2-amino-6-({[(4-iodophenyl)methoxy]carbonyl}amino)hexanoic acid	IPhK	2013	*E. coli*, Yeast and Mammalian	*Mb*PylRS		(189)
**131**	Boronate-l-phenylalanine	(2S)-2-amino-3-(4-boronophenyl)propanoic acid	*p*BorF	2008	*E. coli,* Yeast and Mammalian	*Mj*TyrRS/*Ec*Tyr		(196,197)
**132**	*p*-amino-l-phenlyalanine	(2S)-2-amino-3-(4-aminophenyl)propanoic acid	*p*AF	2002	*E. coli*	*Mj*TyrRS		(192)
**133**	4-(2-tetrazole)-l-phenylalanine	(2S)-2-amino-3-[4-(2H-tetrazol-2-yl)phenyl]propanoic acid	*p*Tpa	2010	*E. coli*	*Mj*TyrRS		(198)
**134**	4-(6-methyl-s-tetrazin-3-yl)amino-l-phenylalanine	(2S)-2-amino-3-{4-[(6-methyl-1,2,4,5-tetrazin-3-yl)amino]phenyl}propanoic acid	TetF	2012	*E. coli*	*Mj*TyrRS		(199)
**135**	4-(6-methyl-s-tetrazin-3yl)-l-phenylalanine	(2S)-2-amino-3-[4-(6-methyl-1,2,4,5-tetrazin-3-yl)phenyl]propanoic acid	Tet-v2.0	2015	*E. coli*	*Mj*TyrRS		(200)
**136**	3-(6-alkyl-s-tetrazin-3-yl)-l-phenylalanine	(2S)-2-amino-3-[3-(6-R-yl-1,2,4,5-tetrazin-3-yl)phenyl]propanoic acid	Tet-v3.0	2020	*E. coli*, Yeast and Mammalian	*Mb*PylRS		(201)
**137**	(2S)-2-amino-3-(6-alkyl-1,2,4,5-tetrazin-3-yl)propanoic acid	(2S)-2-amino-3-(6-R-yl-1,2,4,5-tetrazin-3-yl)propanoic acid	Tet-v4.0	2023	*E. coli*, Yeast and Mammalian	*Mb*PylRS		(202)
**138**	N^ε^-(1-methylcycloprop-2-enecarboxamido)-l-lysine	(2S)-2-amino-6-[(1-methylcycloprop-2-ene-1-carbonyl)amino]hexanoic acid	CpK1	2012	*E. coli*, Yeast and Mammalian	*Mb*PylRS		(203)
**139**	N^ε^-[((2-methylcycloprop-2-en-1-yl)methoxy)carbonyl]-l-lysine	(2S)-2-amino-6-({[(2-methylcycloprop-2-en-1-yl)methoxy]carbonyl}amino)hexanoic acid	CpK2	2014	*E. coli*, Yeast and Mammalian	*Mb*PylRS		(16)
**140**	Norborene-l-lysine1	(2S)-2-amino-6-({[(bicyclo[2.2.1]hept-5-en-2-yl)oxy]carbonyl}amino)hexanoic acid	NorK1	2012	*E. coli*, Yeast and Mammalian	*Mb*PylRS		(204)
**141**	Norborene-l-lysine2	(2S)-2-amino-6-({[(bicyclo[2.2.1]hept-5-en-2-yl)methoxy]carbonyl}amino)hexanoic acid	NorK2	2012	*E. coli*, Yeast and Mammalian	*Mm*PylRS		(205)
**142**	*Trans*-cyclooct-4-ene-l-lysine	(2S)-2-amino-6-[({[(1R,4E)-cyclooct-4-en-1-yl]oxy}carbonyl)amino]hexanoic acid	TCOK	2012	*E. coli*, Yeast and Mammalian	*Mm*PylRs		(206)
**143**	*Trans*-cyclooct-2-ene-l-lysine	(2S)-2-amino-6-[({[(1S,2E)-cyclooct-2-en-1-yl]oxy}carbonyl)amino]hexanoic acid	TCO^a^K	2014	*E. coli*, Yeast and Mammalian	*Mm*PylRs		(207)
**144**	*Trans*-cyclooct-3-ene-l-lysine	(2S)-2-amino-6-[({[(1S,3E)-cyclooct-3-en-1-yl]oxy}carbonyl)amino]hexanoic acid	TCO#K	2014	*E. coli*, Yeast and Mammalian	*Mm*PylRs		(207)
**145**	Bicyclo[6.1.0]non-4-ene-9-ylmethanol-l-lysine	(2S)-2-amino-6-[[9-bicyclo[6.1.0]non-4-enyl]methoxycarbonylamino]hexanoic acid	sTCOK	2012	*E. coli*, Yeast and Mammalian	*Mb*PylRS		(208)
**146**	Bicyclo[6.1.0]non-4-yne-9ylmethanol-l-lysine	(2S)-2-amino-6-[[(1*S*,8*R*)-9-bicyclo[6.1.0]non-4-ynyl]methoxycarbonylamino]hexanoic acid	BCNK	2012	*E. coli*, Yeast and Mammalian	*Mb*PylRS		(208)
**147**	Spiro[2.3]hex-1-ene-l-lysine	(2S)-2-amino-6-({[(spiro[2.3]hex-1-en-5-yl)methoxy]carbonyl}amino)hexanoic acid	SphK	2017	*E. coli*, Yeast and Mammalian	*Mm*PylRs		(209)
**148**	Cyclooctynyl-l-lysine1	(2S)-2-amino-6-({[(cyclooct-2-yn-1-yl)oxy]carbonyl}amino)hexanoic acid	CoK1	2011	*E. coli*, Yeast and Mammalian	*Mm*PylRS		(210)
**149**	Cyclooctynyl-l-lysine2	(2S)-2-amino-6-[({[(cyclooct-2-yn-1-yl)oxy]ethoxy}carbonyl)amino]hexanoic acid	CoK2	2011	*E. coli*, Yeast and Mammalian	*Mm*PylRS		(210)
**150**	Sulfo-l-tyrosine	(2S)-2-amino-3-[4-(sulfooxy)phenyl]propanoic acid	SY	2006	*E. coli*, yeast and Mammalian	*Mj*TyrRS/*Ec*TyrRS		(211,212)
**151**	Acetyl-l-ysine	(2S)-2-amino-6-ethanamido-hexanoic acid	KAc	2008	*E. coli*, Yeast and Mammalian	*Mb*PylRS		(59)
**152**	Pyrrolysine	-	Pyl	-	-	-		(52)
**153**	Propionyl-l-lysine	(2S)-2-amino-6-propanamido-hexanoic acid	Kpr	2013	*E. coli*, Yeast and Mammalian	*Mm*PylRS		(213)
**154**	Butyryl-l-lysine	(2S)-2-amino-6-butanamido-hexanoic acid	Kbu	2013	*E. coli*, Yeast and Mammalian	*Mm*PylRS		(213)
**155**	Crotonyl-l-lysine	(2S)-2-amino-6-[(2E)-but-2-enamido]hexanoic acid	Kcr	2013	*E. coli*, Yeast and Mammalian	*Mm*PylRS		(213)
**156**	Phenyl-l-selenocysteine	(2S)-2-amino-3-phenylselanylpropanoic acid	PhSec	2007	*E. coli*	*Mj*TyrRS	Undergoes reaction to yield Dehydroalanine	(214)
**157**	l-selenalysine	(2R)-2-amino-3-[(2-aminoethyl)selanyl]propanoic acid	SeK	2006	*E. coli*	Ribos. synth	Undergoes reaction to yield Dehydroalanine	(215)
**158**	N^ε^-2-hydroxyisobutyryl-l-lysine	(2S)-2-amino-6-({[(2-hydroxypropan-2-yl)oxy]carbonyl}amino)hexanoic acid	HibK	2015	*E. coli*, Yeast and Mammalian	*Mm*PylRS		(216)
**159**	Phosphonate-l-serine	(2S)-2-amino-4-phosphonobutanoic acid	PS	2018	*E. coli*	*Mm*pSepRS		(217)
**160**	Phospho-l-threonine	(2S)-2-amino-3-(phosphonooxy)butanoic acid	PThr	2017	*E. coli*	*Mm*pSepRS		(218)
**161**	Dimetylamino-phospho-l-tyrosine	(2S)-2-amino-3-(4-{[bis(dimethylamino)phosphoryl]oxy}phenyl)propanoic acid	N,N-PY	2017	*E. coli*, Yeast and Mammalian	*Mm*PylRS		(219)
**162**	l-homoglutamine	(2S)-2,6-diamino-6-oxohexanoic acid	hGln	2004	*E. coli*	*PhK*RS		(29)
**163**	O-phospho-l--serine	(2S)-2-amino-3-(phosphonooxy)propanoic acid	Sep	2011	*E. coli*	*Mm*pRSsep		(33)
**164**	3-methyl-l-histidine	(2S)-2-amino-3-(1-methyl-1H-imidazol-5-yl)propanoic acid	MeH	2014	*E. coli*, Yeast and Mammalian	*Mb*PylRS		(65)
**165**	2-furyl-l-alanine	(2S)-2-amino-3-(furan-2-yl)propanoic acid	Fury-A	2014	*E. coli*, Yeast and Mammalian	*Mb*PylRS		(65)
**166**	2-thienyl-l-alanine	(2S)-2-amino-3-(thiophen-2-yl)propanoic acid	Th-A	2014	*E. coli*, Yeast and Mammalian	*Mb*PylRS		(65)
**167**	2-(5-bromo-thienyl)-l-alanine	(2S)-2-amino-3-(5-bromothiophen-2-yl)propanoic acid	5–Br-Th	2014	*E. coli*, Yeast and Mammalian	*Mb*PylRS		(65)
**168**	3-pyridyl-l-alanine	(2S)-2-amino-3-(pyridin-3-yl)propanoic acid	PylA	2014	*E. coli*, Yeast and Mammalian	*Mb*PylRS		(65)
**169**	3-(naphthalen-2-ylamino)-2-aminopropanoic acid	(2S)-2-amino-3-[(naphthalen-2-yl)amino]propanoic acid	Nap	2009	Yeast and Mammalian	*Ec*LeuRS		(101)
**170**	3-(2-naphthyl)-l-alanine	(2S)-2-amino-3-(naphthalen-2-yl)propanoic acid	2-NapA	2002	*E. coli*	*Mj*TyrRS		(220)
**171**	Biphenyl-l-alanine	(2S)-2-amino-3-([1,1′-biphenyl]-4-yl)propanoic acid	BipA	2007	*E. coli*	*Mj*TyrRS		(131)
**172**	α-aminocapryilicacid	(2S)-2-aminooctanoic acid	Amca	2004	Yeast and Mammalian	*Ec*LeuRS		(48)
**173**	2-aminononanoic acid	(2S)-2-aminononanoic acid	Amna	2008	Yeast and Mammalian	*Ec*LeuRS		(221)
**174**	2-aminodecanoic acid	(2S)-2-aminodecanoic acid	Amda	2008	Yeast and Mammalian	*Ec*LeuRS		(221)
**175**	2-amino-5-thio-valeric acid	(2S)-2-amino-5-sulfanylpentanoic acid	AmSVa	2008	Yeast and Mammalian	*Ec*LeuRS		(221)
**176**	2-amino-6-thio-caproic acid	(2S)-2-amino-6-sulfanylhexanoic acid	AmSca	2008	Yeast and Mammalian	*Ec*LeuRS		(221)
**177**	2-amino-methyl-thio-valeric acid	(2S)-2-amino-5-(methylsulfanyl)pentanoic acid	AmmSva	2008	Yeast and Mammalian	*Ec*LeuRS		(221)
**178**	2-amino-methyl-thio-caproic acid	(2S)-2-amino-6-(methylsulfanyl)hexanoic acid	AmmSca	2008	Yeast and Mammalian	*Ec*LeuRS		(221)
**179**	*o*-methyl-l-tyrosine	(2S)-2-amino-3-(4-methoxyphenyl)propanoic acid	mY	2001	*E. coli*, Yeast and Mammalian	*Mj*TyrRS/*Mb*PylRS		(3,116)
**180**	*p*-isopropyl-l-phenylalanine	(2S)-2-amino-3-[4-(propan-2-yl)phenyl]propanoic acid	*p*IF	2002	*E. coli*	*Mj*TyrRS		(192)
**181**	*p*-carboxyl-l-phenylalanine	4-[(2S)-2-amino-2-carboxyethyl]benzoic acid	*p*CF	2002	*E. coli*	*Mj*TyrRS		(192)
**182**	dithiolane-l-phenylalanine	(2S)-2-amino-3-{4-[2-(1,2-dithiolan-3-yl)acetamido]phenyl}propanoic acid	dtF	2019	*E. coli*	*Mj*TyrRS		(222)
**183**	*tert*-butyloxy-l-lysine	(2S)-2-amino-6-[(tert-butoxycarbonyl)amino]hexanoic acid	Boc-K	2008	*E. coli*, Yeast and Mammalian	*Mm*PylRS		(57)
**184**	N^ε^-benzyloxycarbonyl-l-lysine	(2S)-2-amino-6-{[(benzyloxy)carbonyl]amino}hexanoic acid	Z-K	2008	*E. coli*, Yeast and Mammalian	*Mm*PylRS		(57)
**185**	N^ε^-cyclopentyloxycarbonyl-l-lysine	(2S)-2-amino-6-{[(cyclopentyloxy)carbonyl]amino}hexanoic acid	Cyc	2006	*E. coli*, Yeast and Mammalian	*Mb*PylRS		(55)
**186**	N^ε^-d-prolyl-l-lysine	(2S)-2-amino-6-[(pyrrolidine-2-carbonyl)amino]hexanoic acid	ProK	2006	*E. coli*, Yeast and Mammalian	*Mb*PylRS		(55)
**187**	N^ε^-(3-methyl-2,3-dihydro-pyrrol-2-yl)carbonyl-l-lysine	(2S)-2-amino-6-{[(3S)-3-methyl-2,3-dihydro-1H-pyrrole-2-carbonyl]amino}hexanoic acid	PylE	2006	*E. coli*, Yeast and Mammalian	*Mb*PylRS		(55)
**188**	d-tyrosine	(2R)-2-amino-3-(4-hydroxyphenyl)propanoic acid	d-Tyr	2016	*E. coli* and Yeast	*Ec*TyrRS	Not incorporated yet	(223)
**189**	3-benzothienyl-alanine	2-amino-3-(1-benzothiophen-3-yl)propanoic acid	BTA	2020	*E. coli*, Yeast and Mammalian	*Mm*PylRS		(224)
**190**	1-methyl-l-tryptophan	(2S)-2-amino-3-(1-methyl-1H-indol-3-yl)propanoic acid	MeTrp	2020	*E. coli*, Yeast and Mammalian	*Mm*PylRS		(224)
**191**	1-formyl-l-tryptophan	(2S)-2-amino-3-(1-formyl-1H-indol-3-yl)propanoic acid	ForTrp	2020	*E. coli*, Yeast and Mammalian	*Mm*PylRS		(224)
**192**	3-(1-naphthyl)-l-alanine	(2S)-2-amino-3-(naphthalen-1-yl)propanoic acid	1-NapA	2020	*E. coli*, Yeast and Mammalian	*Mm*PylRS		(224)

a Organisms in which the AARS used is orthogonal.

b The AARS used for the ncAA incorporation.

c Reference for the ncAA incorporation by different systems.

Overall, ncAAs can be grouped into two major categories: the direct probes and the “click” ncAAs. We define as direct probes AAs that serve themselves as biochemical or biophysical probes for a variety of studies without any further modification. Examples of these include ncAAs bearing heteroatoms for X-ray scattering, isotopes for nuclear magnetic resonance (NMR), fluorescent moieties for imaging or biosensors, azido and cyano groups for infrared (IR) spectroscopy, cross-linking ncAAs, and many others. The advantage of such ncAAs, which do not require extra processing for functionality after incorporation in the protein of interest, is sometimes counterbalanced by challenges related to cellular uptake and stability, as well as size limitation. For example, the EPR probe nitroxide lysine (SLK-1 **31**), while commonly used for structural dynamics studies of proteins, is reportedly unstable in biological conditions.^93,94^ In addition, there are known limitations of the size of AAs that the ribosome can accept.^95^

“Click” ncAAs do not have any special biophysical feature but carry on the side chain a chemically reactive group for bioorthogonal chemistry. They can undergo rapid conjugation reactions either under physiological conditions in live cells or after purification *in vitro* and allow for the incorporation in the protein of almost any desired probe. For example, “click” ncAAs can be reacted with a fluorophore conjugated to a bioorthogonal reactive group for introducing site-specific fluorescent tags on expressed proteins. NcAAs bearing double or triple bonds, strained rings, azides and certain other reactive groups have been used for different click reactions. One advantage of “click” ncAAs is that if the handle and tag are compatible and selective toward each other, the same AA can be clicked to different probes that can be used in diverse downstream applications. For example, pAziF **32** can react with alkyne-dyes for imaging applications, as well as with alkyne-nitroxide molecules for EPR studies.^96,97^ Important considerations when choosing “click” AAs for use is their selectivity and reactivity with desired reaction partners at physiological pH, under ambient conditions, and without causing toxicity in the host organism.^37^

#### 2.2.1 Direct Probes

##### 2.2.1.1 Fluorescent AAs (FlAAs)

Fluorescent AAs (FlAA) serve as valuable probes in light spectroscopy measurements. Their size is often comparable to canonical AAs, and the absence of a linker between the backbone and fluorophore makes FlAAs reporters for local protein regions. FlAAs generally emit blue to cyan light (350–500 nm), are not very bright and are susceptible to quenching by endogenous AAs such as Phe, Tyr and Trp. While this quenching phenomenon has been used to monitor residues rearrangement following protein activation, it presents challenges when incorporating FlAAs into proteins for imaging and other applications that require clear, bright and localized signals. A notable feature shared by many of these ncAAs is their sensitivity to environmental changes. This sensitivity translates into the capacity for them to alter their emission wavelength and intensity in response to changes in the polarity of their environment. This quality is by far one of the most important characteristics of small fluorophores and has been used in studies to probe protein conformational changes, including those that occur upon ligand binding.

Although the number of FlAAs amenable for use in GCE is somewhat limited, various moieties have been incorporated in proteins both *in vitro* and in live cells and in both bacterial and mammalian systems. Dansyl AAs were among the first fluorescent AAs synthesized and introduced into peptides.^225^ Dansylalanine **1**, with an emission between 500 and 580 nm depending on the environment, was later incorporated into proteins using an orthogonal AARS/tRNA system, enabling the study of protein environmental changes.^226^ The spectral properties of hydroxycoumarin derivatives were first investigated in the 1970s, when these molecules gained notoriety for being sensitive to environmental changes.^227^ However, it took another 30 years before an ncAA coupled to coumarin was synthesized and incorporated into proteins in bacteria and mammalian cells. Coumarin AAs (e.g., HCou **2**, HCK **3**) absorb 380 nm light, emit at around 450 nm and exhibit sensitivity to pH and solvent polarity.

The Prodan fluorophore has a long history as a membrane dye in the left side of the visible spectrum. Although Prodan derivatives lack pH sensitivity, they show pronounced solvatochromic properties in solvents of different polarities. With a fixed absorption maximum around 350 nm, their emission ranges from 380 to 520 nm, because of a push–pull mechanism between the keto and amine groups at opposite ends of the naphthalene ring. Quantum yields diminish with local polarity, and their fluorescence can be quenched by Trp. Consequently, careful consideration of the surrounding protein environment is essential before choosing a site for incorporation. Compared with other small fluorescent moieties like dansyl and coumarin, Prodan derivatives are brighter and more environmentally sensitive.^228^ One such derivative carrying the Prodan fluorophore, called AlaDAN **4**, was first incorporated into both soluble (B1 domain of protein G (GB1)) and membrane proteins (Kir2.1 and Shaker potassium channels) using an amber stop codon and injection of AlaDAN-charged suppressor tRNA in *Xenopus* oocytes.^100^ However, it was later disregarded in favor of its structural analogue 3-(6-acetylnaphthalen-2-ylamino)-2-aminopropanoic acid (Anap **5**). The structure of Anap is similar to that of AlaDAN, but with the AA backbone leading from the amine group instead. Anap was incorporated in *E. coli* glutamine binding protein using amber codon suppression with an optimized AARS/tRNA pair orthogonal in yeast and mammalian cells.^101^ Since then, it has been used as a probe for live cell monitoring site-specific conformational rearrangements of proteins upon ligand binding, two-photon excitation fluorescence confocal microscopy and patch-clamp fluorimetry. Anap has also been employed as a Förster resonance energy transfer (FRET) partner for dynamics studies of both cytosolic and membrane proteins in cell membranes. Anap can act as FRET donor in transition metal ion FRET (tmFRET), coupled to Co^2+^ or Cu^2+^ (ACCuRET) and can monitor distance changes between receptor domains and the cell membrane upon receptor activation.^229^ Despite Anap’s widespread use in recent years, significant drawbacks that limit its broader utility are the challenging synthesis process that limits the yield, and the resulting high cost of the commercial product. Together, these factors pose a challenge, particularly for large-scale protein expression in mammalian cells.

An alternative to Prodan showing better photochemical properties emerged in recent years in the form of Acridonylalanine (Acd **6**). Acd is an Acridine derivative that displays high fluorescent quantum yield in the blue emission spectrum. Its excitation wavelength is similar to that of Prodan derivatives, albeit slightly red-shifted, with an excitation maximum around 380–400 nm, while its emission maximum is at 445 nm.^230^ A remarkable advantage of Acd is its long photodurability that allows its use in fluorescence lifetime measurements. Originally synthesized in 2003, Acd was incorporated in proteins and antibodies (including streptavidin and cSC) *in vitro* by chemical aminoacylation and a four-base codon/anticodon strategy and used in diverse fluorescence measurements.^231^ In the following years, the ability of Acd to act as an acceptor or donor fluorophore in FRET expanded its use in peptide synthesis to follow thermal denaturation. It was not until 2013, with the identification of a *Mj*AARS/tRNA capable of incorporating Acd at amber stop codons in bacteria, that the ncAA was truly exploited, with Acd being incorporated for imaging and FRET studies.^102^ The 2021 development of an AARS/tRNA pair for Acd that was orthogonal in mammalian systems further increased its application in live-cell microscopy.^103^ A photoactivable Acd derivative (sAcd **7**) was also introduced into proteins by the *Mj* AARS/tRNA pair for *in vitro* studies.^104^ Additional derivatives of Acd such as Aad **8** and Dad **9** were synthesized, yielding red-shifted fluorophores with increased quantum yields and longer fluorescence lifetimes.^105,232^ Both derivatives are readily incorporated in peptides and used in FRET assays, but their genetic incorporation has not yet been reported.

##### 2.2.1.2 Biophysical Probes

The spectroscopic analysis of proteins using NMR, EPR and IR methods becomes increasingly challenging as the size of the system of interest increases. Complex spectra with overlapping signals from the protein backbone can be difficult to interpret. Selective labeling of protein positions with unusual chemical groups can aid in the analysis of results by reducing the total number of peaks or by shifting peaks to empty spectral areas. Traditional techniques for selective labeling of single protein residues for NMR studies involve mutating all other residues of the same type, i.e., depleting the endogenous AA of choice and replacing it with an isotopically labeled AA.^233^ In EPR studies, a similar approach relies on the introduction of a Cys in the position of interest, while deleting all other solvent exposed Cys residues in the protein, and then labeling the target Cys residue with a probe carrying a free electron.^234^ These approaches may have drawbacks, such as mutations affecting the protein’s structure and function and the need for cell-free expression. GCE offers the advantage of site-specific incorporation of ncAAs bearing heteroatoms or moieties directly into the protein of interest in live cells. Robust incorporation of ncAAs in bacteria facilitates high yields of protein expression and solves the problem of having different populations of protein, one with the probe and one without. In this context, various chemical groups have been introduced in the side chains of ncAAs for use in spectroscopy.

Iodine (**35**–**36**) has been introduced to aid in data analysis of X-ray scattering of large proteins, while cyano and azido groups (**32**–**34**) have been successfully incorporated to monitor vibrational stretches in proteins with Fourier-transform IR (FT-IR) difference spectroscopy.^235,236^ NcAAs carrying ^15^N and ^13^C isotopes or fluorides (**11**–**28**) been also been employed for 1D and 2D NMR, while nitroxides (**29**–**31**) have been incorporated for EPR studies.

##### 2.2.1.3 Cross-linkers

Cross-linking moieties create covalent bonds between molecules located in close spatial proximity to each other. Since the 1960s, researchers have incorporated cross-linking moieties into peptides and small molecules to investigate ligand-protein interactions.^237−239^ With the advent of GCE, ncAAs for cross-linking could be directly and site-specifically incorporated into proteins to study a variety of phenomena both *in vitro* and in live cells. The application of cross-linkers has yielded significant insights into the study of membrane receptors, which are particularly challenging to isolate and whose functionality may depend on interactions within the cellular membrane. Cross-linking techniques have become particularly useful when little information is known about a protein’s structure or function.^142^ Overall, genetically incorporated cross-linkers can help researchers uncover important information about protein structure and especially native interactions, which is difficult to achieve with other techniques.

Cross-linking AAs can be divided into two broad classes: photo-cross-linkers (**32**, **42**–**57**) and chemical cross-linkers (**62**–**82**). Additionally, bifunctional cross-linking ncAAs that are both photoactivatable and chemically reactive have been presented (**44**–**45**, **51**–**52**). Cross-linking ncAAs and their applications have been thoroughly reviewed in other works, to which we refer the reader for further details.^85,240−243^

Photo-cross-linkers are molecules that are chemically stable in biological conditions but become highly reactive upon photolysis with UV light. Activation of these moieties leads to the formation of a reactive radical, which forms a covalent bond with nearby organic molecules. Installing a photo-cross-linker into a protein gives the possibility to capture stable or transient interactions either inter- or intramolecularly. The most used genetically encoded AAs for photo-cross-linking are pBpa **42** and pAziF **32** followed by other ncAAs featuring a diazirine cross-linker, such as for instance DizPK **47**. Photo-cross-linkers have also been used as proximity sensors to probe the interactions of proteins of interest in the cellular environment.

In contrast, chemical cross-linkers do not generally need to be activated since they are intrinsically reactive. Their chemistry tends to be more specific than that of photo-cross-linkers, in that they react only with selected functional groups. They often feature a mildly electrophilic carbon (made so by the vicinity of an electron-withdrawing group), which undergoes a nucleophilic attack by electron-rich AA side chains in the local protein environment, usually the thiol group of a Cys residue. This reaction, which is slow when both groups are free to move in solution, has exponentially faster reaction rates and higher yields when the two moieties come close to each other (proximity-enhanced reactivity).^156^ Chemical cross-linkers may exhibit higher yields than photo-cross-linkers, and their specificity toward a subset of residues in the pray molecule gives a more uniform population of cross-linked products, thereby making mass spectrometry (MS) analysis of cross-linked products easier.^85,244^

Combinations of photo- and chemical properties have recently been exploited through the genetic encoding of photoactivatable ncAAs that chemically cross-link to subsets of nucleophilic amino acids, but only after photoactivation. These inert “caged” chemical cross-linkers are “de-caged” with UV light to yield a chemically reactive moiety, which is much more reactive than ordinary chemical cross-linkers yet more selective than classical azide, benzophenone and diazirine photo-cross-linkers. These ncAAs have enabled studies requiring temporal resolution.^143,147^

From a structural point of view, cross-linking AAs can be classified into two families: Tyr-based and Pyl-based. The size and physicochemical properties of the ncAAs must be considered when designing experiments, since the length of the AA side chain will define a different three-dimensional radius of action for capturing potential interaction partners. Tyr analogues are best used for probing short-range interactions of 6–8 Å, while Pyl analogues cover a larger effective range of 14–15 Å.

##### 2.2.1.4 Photoswitchable and Photocaged AAs

The concept of using light to modulate protein activity originated in neuroscience and gave rise to the field of optogenetics.^245−247^ Soon after, small molecules, either light-responsive molecular cages (photocages **3, 83–100**) or switches (photoswitches **101**–**107**), were used in protein studies to achieve spatiotemporal control of protein function. Photocaged ncAAs are endogenous AAs that are “protected” to mask the group on the side chain with steric bulk, making it nonfunctional. These AAs are typically incorporated in positions that are involved in the function of the original protein. Upon UV light irradiation, the masking group is removed, thus decaging the AA and reinstating the residue’s activity. On the other hand, photoswitches are molecules that undergo a reversible isomerization (normally *cis–trans* geometric stereoisomerization) upon UV light irradiation. When incorporating a photoswitchable-AA into a protein of interest, this conformational change can turn on protein activity, as the *cis* conformation is usually more compact and less obstructive to the active pocket than the *trans* conformation.

#### 2.2.2 CLICK-ncAAs

**Figure 3:**
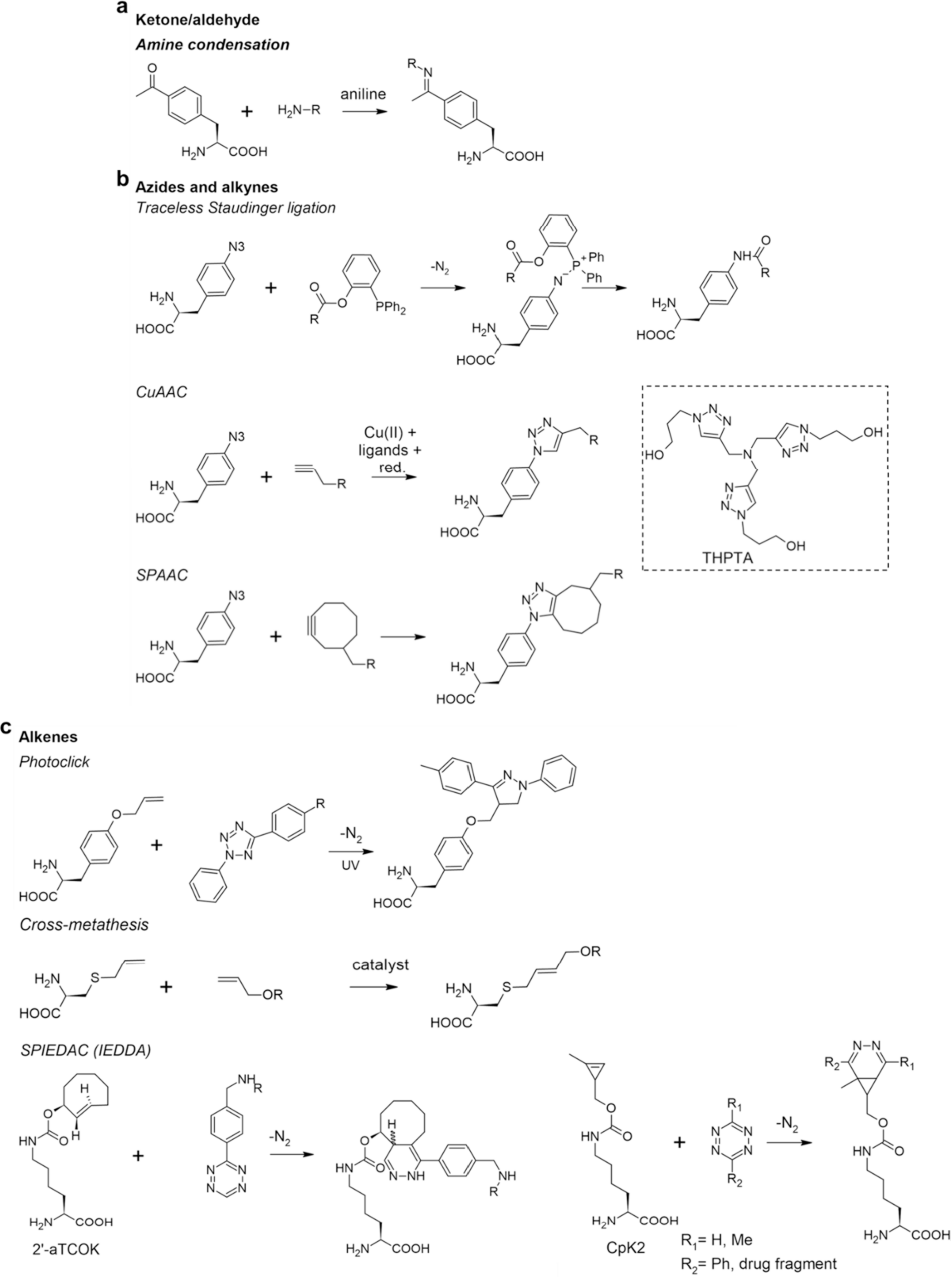

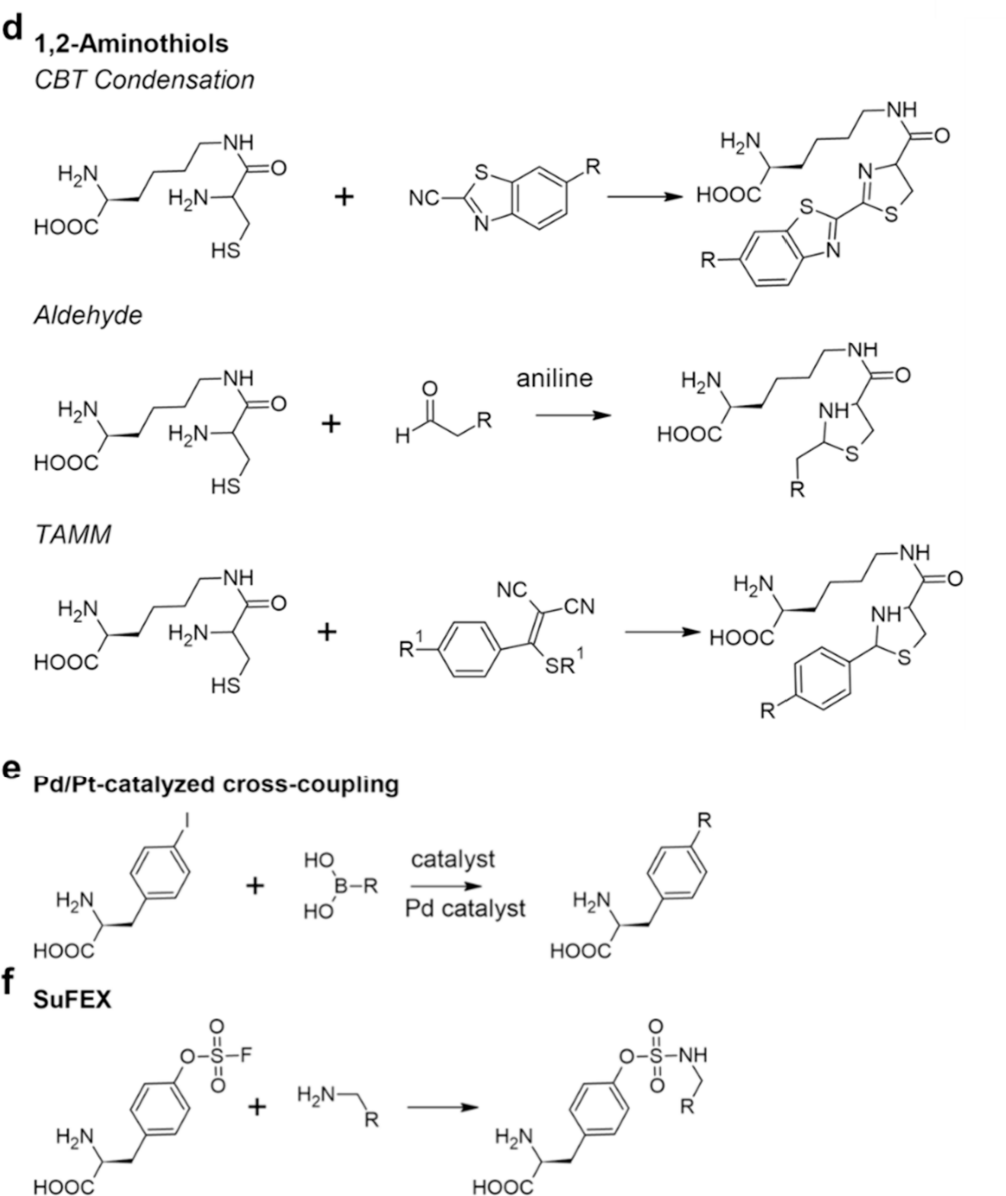
Bioorthogonal reactions. (a) Reaction of ketones/aldehydes with amines; (b) reactions of alkynes and azides; (c) reactions of alkenes; (d) reactions of 1,2-aminothiols; (e) Pd/Pt cross-coupling reaction; (f) SuFEX reaction with amines.

“Click”-ncAAs carry on the side chain a chemical anchor for bioorthogonal chemistry. The term *bioorthogonal reactions* was coined in the early 2000’s and refers to all chemical reactions that do not interfere with biological processes, can happen in biological conditions at fast rates and are selective toward their substrate without cross-reactivity with endogenous functional groups.^248^ Bioconjugation reactions have been first developed for targeting specific endogenous moieties such as the AAs Cys and Lys.^249^ As a downside, all residues of these types in the system of interest (normally more than one) can react, so specificity of conjugation cannot be always guaranteed. Since an ncAA can be incorporated site-specifically at almost any position in a protein of interest, GCE provides obvious advantages. Rapid biorthogonal reactions have been developed to facilitate the labeling of proteins harboring ncAAs for both *in vitro* and *in vivo* studies (see Figure 3). We summarize here ncAAs suitable for bioconjugation, and we refer the reader to dedicate reviews for further details.^6,37,250,251^

##### 2.2.2.1 Ketones

Keto and beta-diketo AAs (pAcF **118**, mAcF **119**, pBpa **42** and dkF **120**) can undergo reaction with hydrazines and hydroxylamines. The condensation of ketones or aldehydes with amines was among the first bioorthogonal reactions used to target site-directed incorporated ncAAs, as the ketone *p-*acetyl-Phe (*p*AcF **118**) was labeled *in vitro* with a hydrazide-dye and hydrazide-biotin.^10,252^ Later, the labeling of chemokine receptor C–C Chemokine Receptor 5 (CCR5) and visual rhodopsin was demonstrated *in vitro* by incorporating pBpa and pAcF in mammalian cells and clicking with fluorescein-hydrazide.^14^ The reaction has slow kinetics (requires overnight incubation) and runs at low pH, so that it is not suitable for live-cell labeling.^37^ Although the introduction of catalysts has later made it possible at physiological pH,^253^ other chemistries are preferred for live-cell applications. In principle, both ketone and aldehydes are suitable anchors for this reaction, but aldehydes are too reactive toward biomolecules, which limits the possibility of genetically encoding them.^251^

##### 2.2.2.2 Azides

The azide moiety is small, is stable under physiological conditions and can be employed with high orthogonality.^254^ Azido AAs (pAziF **32**, ACPK **109**, o-AzbK **110**, and AzK **108**) react either with terminal alkynes via copper catalyzed alkyne–azide cycloaddition (CuAAC), with strained alkynes via strained-promoted azide–alkyne cycloaddition (SPAAC), or with phosphines via the Staudinger ligation.

The **CuAAC** reaction of azides with terminal alkynes to yield triazoles, which normally would require high temperature and pressure, was found to take place with high yields at room temperature when in the presence of a Cu(I) catalyst.^255,256^ The reaction is moderately fast (rate constant of 10–200 M^–1^ s^–1^, approximately 10–30 min) and has been used to label many proteins *in vitro*, as well as on the cell surface. To reduce the toxicity of Cu(I) for live-cell applications, several ligands have been introduced in the reaction, which protect cells from reactive oxygen species (ROS), by allowing reduction of copper(II) salts to copper(I) *in situ*.^96,257−259^

Still, the presence of copper in the system is problematic for studies that require long-term exposure to this reagent. A way to eliminate the requirement of the copper catalyst is the introduction of a strain in the alkyne moiety. The obvious advantage of the **SPAAC** reaction over CuAAC is the absence of Cu(I), but the reaction is much slower in aqueous conditions (rate constant of <0.1 to 1 M^–1^ s^–1^, approximately 30 min to 2 h).^8^

The **Staudinger ligation** (and its traceless variation) does not require any catalyst. The reaction occurs between an azide and a phosphine and results in the formation of an amide link.^260^ Staudinger ligation on genetically incorporated azides has been shown for *in vitro* labeling of mammalian rhodopsin and for tagging receptors in ELISA assays.^261,262^ However, the reaction kinetics are quite slow (rate constant of ∼0.0025 M^–1^ s^–1^, approximately 30 min to 4 h) and are less compatible with live cell experiments. Moreover, the reaction suffers from phosphine oxidation, therefore requiring a high concentration of reagent, which is not always feasible due to nonspecific background reactivity.^37,248^

CuAAC and SPAAC reactions can be performed by inverting the partners and incorporating either an alkene or alkyne moiety into the protein to react it post-translationally with an azide reagent. This may be advantageous in certain cases, as azides are partially reduced in the cell.^248,263^ To enhance bioorthogonal labeling of membrane proteins, reaction rates can be proximity-enhanced using hydrophilic moieties that partition into membranes or detergent micelles, which increases the local reactant concentrations.^264^

##### 2.2.2.3 Alkenes and Alkynes

NcAAs carrying alkene or alkyne groups are important for post-translational modification of protein via conjugation reactions. Double or triple bonds can be placed at a terminal position or be embedded in strained rings in the functional moiety, which makes an enormous difference in their reactivity. Terminal alkenes are themselves poorly reactive under biological conditions but can be activated with UV light to generate radical species that react rapidly with either proximal thiols (photoinduced thiol–ene coupling, as in the case of photoswitchable click AAs - PSCaa)^182^ or tetrazoles (**photoclick cycloaddition**).^265−267^ Examples of such AAs are O-AllY **121**, AcrK **64**, and AlocK **122**, which have been incorporated and labeled in bacteria and mammalian cells.^80,203,265,268,269^ One advantageous aspect of the photoclick cycloaddition is that it can be fluorogenic (i.e., generate a fluorescent moiety after clicking), an interesting feature for imaging experiments. Unfortunately, it is difficult to rule out possible reactivity of the triazole with alkenes present in the cellular environment, such as in membrane lipids.^203^ Inversely, a tetrazole AA (pTpa **133**) has also been incorporated in the protein of interest and labeled with a dye-fumarate after only 5 min of UV irradiation.^198,270^ Application of this technique offers the exciting possibility of two-photon excitation microscopy for spatiotemporal control of labeling;^271^ however, application in live cell studies presents some concerns over possible cross-reactivity of endogenous alkene moieties.

Terminal alkynes (pPrgF **114**, AlkK **115**, AlkK2 **116**, EtcK **117**) react with azides via CuAAC, which represents the same reaction described above from the perspective of the azide-ncAAs. These AAs have been incorporated into proteins for site-specific labeling *in vitro*, first in yeast and in bacteria,^125,187^ and then in mammalian cells.^45^ In turn, strained alkynes (BCNK **146**, CoK1 **148**, CoK2 **149**) can react with azides via SPAAC.^210,272^

However, possibly the most popular reaction for protein labeling nowadays is the inverse-electron-demand Diels–Alder cycloaddition (**SPIEDAC**) between strained alkenes or alkynes and tetrazines. A strained-alkene or -alkyne has been shown to react with tetrazine quickly and at physiological conditions in as fast as 5 min,^273^ making it possible to label proteins both *in vitro* and *in vivo*.^204,208,274,275^ This reaction has greatly advanced the field of live-cell studies, becoming the first system of choice in localization studies involving ncAAs. This chemistry provides the advantage of fast kinetics, great compatibility and absence of toxicity in the cellular environment and does not need any catalyst or activation with light. Several ncAAs have been genetically encoded for SPIEDAC reactions such as TetF **134** and the strained alkenes (CpK **138**–**139**, NorK1 **140**, NorK2 **141**, TCOK **142**–**145**, and SphK **147**) and alkynes (BCNK **146**, CoK1 **148**, CoK2 **149**). All these ncAAs feature bulky side chains and their incorporation is made possible by the extraordinary flexibility of the PylRS system. The strained rings feature different stability and reactivity in cellular milieu.^276^ Details regarding these AAs have been thoroughly reviewed elsewhere.^37^

Particularly, tetrazine-dyes for clicking via SPIEDAC to TCOK have been selected as superior dyes given their low background reactivity and relatively small side-chain size.^277^ The SPIEDAC reaction has also become popular for the development of turn-on probes. The tetrazine, being electron-withdrawing, can quench or strongly diminish the brightness of the dye when directly coupled to a fluorophore. Only upon clicking of the tetrazine to the strained ncAA, is the electron-withdrawing group removed, and the dye’s fluorescence is restored.^204,265,278^ The reaction has provided a satisfactory method that can be used for super-resolution microscopy and intracellular labeling in intact cells, where an important concern is background noise from unreacted dyes “trapped” in the cytosolic environment that are difficult to wash away. Moreover, the reaction between SphK and a sterically shielded tetrazole dye was demonstrated in the labeling of the glucagon receptor (GCGR) in live mammalian cells and revealed a very long half-life of the construct, compared with the parent unshielded reaction, which is an important characteristic for spatiotemporal measurements.^269^

##### 2.2.2.4 Fluorosulfates

A bioorthogonal reaction involving aryl fluorosulfates (**58**–**61**) has also become popular given its apparent utility under biological conditions. The sulfur-fluoride exchange (SuFEx) has seen widespread use for proximity-enabled reactivity with Lys, Tyr and Ser. The reaction is particularly useful because an efficient reaction is only achieved when the fluorosulfate is in very close proximity with the targeted AA residues. SuFEX has been applied to cross-linking studies, where an AA bearing an aryl fluorosulfate was incorporated into proteins of interest and used to “capture” Lys, Tyr or Ser residues in close proximity.^151,279^ SuFEX chemistry with ncAAs has been recently reviewed elsewhere.^85,280^

##### 2.2.2.5 Others

1,2-Aminothiols have also been incorporated into proteins (ThiPK **128**, DCysK, LCysK **129**). The coupling of aldehydes to 1,2-aminothiols in the presence of anilines has been demonstrated *in vitro* under neutral conditions.^281^ Most recently, the reaction of 1,2-aminothiols with ((alkylthio)(aryl)methylene)malononitrile (TAMM) was shown to be compatible for the labeling of membrane proteins in live mammalian cells.^282,283^ 1,2-Aminothiols can react also with cyanobenzo-thiazoles (CBT), a reaction that can be orthogonal in proteins, providing that the protein of interest does not harbor Cys residues in the terminal tails.^194,284−286^ This labeling technique is quite fast, but suitable only *in vitro*, given the possibility of cross-reactivity of CBT with pyruvates or other cellular metabolites.

Other AAs have been incorporated and labeled in proteins, bearing halides (pIodoF **36**) and boron (pBorF **131**) that can undergo Pd-mediated cross-coupling or react with cyanobenzothiazole, respectively.^284,287^ Regarding the Pd coupling, the initial slow kinetics and high temperatures were overcome by introducing a Pd catalyst (Pd(OAc)_2_(ADHP)_2_) able to label bacterial proteins *in vivo*. Metal catalysts can also provide alternative reactions for clicking mechanisms on ncAAs. Pd-catalyzed cross-coupling via the Suzuki-Miyaura reaction accomplished the clicking of molecules with an alkyl or alkenyl group to *p*-IodoF **36**.^288^ Pd-assisted coupling is compatible under physiological conditions, but the reaction is quite slow (1 h at 37 °C). Pt has also been used to catalyze cross-coupling reactions.^289^

Additionally, anilines (pAF **132**) have also been incorporated into proteins and reacted with phenylenediamine groups.^290,291^ This reaction is incompatible with reductants in the environment since an oxidant is necessary for the reaction to proceed.

### 2.3 Classical Methods for Protein Labeling

Protein studies often require introducing modifications into the protein of interest. For instance, proteins can be equipped with epitope tags for immunodetection and purification, fluorescent tags for localization and interaction studies, mimetics of post-translational modifications for functional studies and so on. Chemical and molecular biological strategies are available to modify proteins.

Chemical modifications of proteins occur naturally at the most reactive AA side chains and include predominantly oxidations, reductions, and both electrophilic and nucleophilic substitutions. In studies of protein structure and function, including in the course of protein sequencing before the age of molecular cloning, a wide range of reagents have been developed to modify specifically AA side chains, in some cases to induce peptide-bond cleavage reactions. Typical protein-reagent induced reactions include oxidations or reductions, alkylations and aromatic ring substitutions. Reagents designed to modify AA side chains of a particular type, for example Cys or Lys residues, are therefore residue-specific. Except in unusual cases,^292^ classical protein-labeling reactions are rarely site-specific.

**Figure 4:**
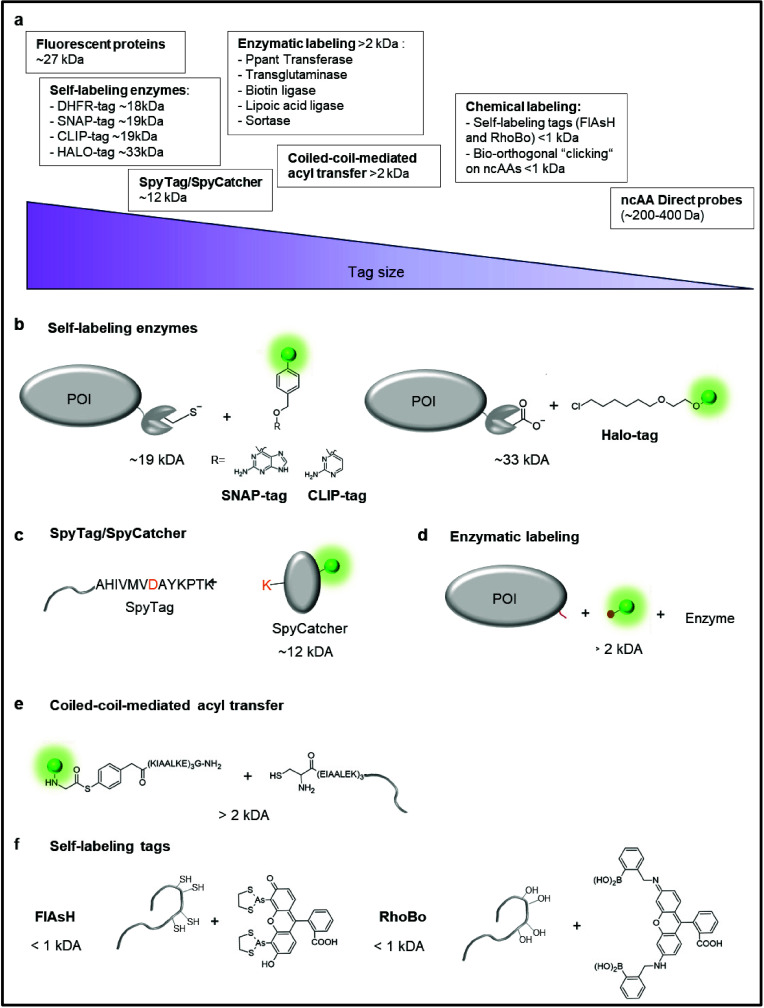
Protein labeling strategies. (a) Summary of common protein tags arranged by size. (b) Self-labeling protein tags SNAP and CLIP are derived from O6-methylguanine-DNA methyltransferase and can be fused to POIs for labeling with benzylguanine or benzylcytosine derivatives, respectively. HaloTag is derived from haloalkane dehalogenase and can be fused to POIs for labeling with chloroalkane linkers. (c) SpyTag/SpyCatcher labeling strategy. (d) Enzymatic labeling of a POI containing a short recognition peptide (orange string) with a fluorescent substrate (orange circle). (e) Template-directed labeling of POI with a fluorophore (green circle) using size-matched coiled-coil peptide secondary structure motifs. (f) Self-labeling tags FlAsH (fluorescein arsenical hairpin binder) and RhoBo (rhodamine bisboronic acid) bind to genetically encoded tetraCys and tetraSer motifs in the POI (gray ribbon), respectively.

Techniques of molecular biology have added an entirely new dimension to the study of proteins in general, and membrane proteins in particular. Fluorescent labels for imaging studies play a predominant role in this context. In the section below, we survey current molecular biology-based strategies for protein labeling, with a major focus on the introduction of fluorescent tags. Our logic follows the decreasing size of the tag, from fluorescent proteins and self-labeling enzymes to smaller tags that can be labeled enzymatically, up to single-residue labeling (Figure 4a). Together with the attempt of reducing the protein size to minimize the perturbation of structure and function of the target protein, parallel efforts aim at enhancing the photochemical properties of the tag, such as brightness and photostability. In general, the size of the tags or labels introduced by classical molecular biology methods is often quite large and can result in substantial perturbation of the system. GCE offers the unique possibility of introducing small tags or modifications in a minimally invasive and site-specific fashion.

#### 2.3.1 Fluorescent Proteins

Fluorescent proteins (FPs), in particular green fluorescent protein (GFP) and its engineered and enhanced versions, have gained widespread use since their discovery in jellyfish in the 1960s and their first application as protein tags in the 1990s. GFP has been fused to expressed proteins of interest in both bacterial and mammalian cells and used for localization studies and other applications.^293−296^ A huge variety of FPs have been characterized and applied to biological studies, with a database inventory available at FPbase.org boasting more than 900 entries. In general, an FP with the desired photochemical characteristics is fused to the protein of interest at either the N-terminal tail or C-terminal tail. However, circularly permuted (cp) GFP constructs can be engineered into internal parts of the protein if protein function is not impaired.^297−299^ While expression of FPs generally does not cause cellular toxicity, FPs are relatively large (∼27 kDa) and may impair intracellular signaling or protein function, especially given their mild tendency to oligomerize. Furthermore, most FPs are susceptible to photobleaching and are not particularly photostable, especially in the red or far-red region.

#### 2.3.2 Self-Labeling Tags and Enzymes

Several small-molecule organic dyes feature superior photostability and photobleaching resistance compared with fluorescent proteins and are better tools for high-end microscopy applications, such as for instance super-resolution microscopy.^207,300^ There are several strategies to introduce such dyes into a protein of interest.

Self-labeling enzymes (Figure 4b) are suicide enzymes that transfer tags, usually fluorophores, from a suitable donor molecule to themselves. Two common self-labeling protein tags are SNAP and CLIP. They were both derived from DNA repair enzymes that accept *O*^*6*^-benzylguanine derivatives or *O*^2^-benzylcytosine derivative substrates. SNAP and CLIP tags are ∼180-AA residues in length (19.4 kDa) and can be fused to either the N- or C-terminal tails of expressed proteins (Figure 4c). Fluorophores coupled to the benzylguanine or benzylcytosine with an intervening linker can be added to cells or to samples *in vitro* to facilitate highly specific enzymatic covalent-coupling reactions.^301,302^ Similarly, the TMP-tag is a modified dihydrofolate reductase enzyme (∼18 kDa) that reacts with folate analogues.^303^ Another popular self-labeling protein tag is the HaloTag, an engineered haloalkane dehalogenase that reacts with alkyl-halide substrates.^304^ The HaloTag is particularly useful for labeling proteins with fluorophores, although its large size at 297-AA residues in length (∼33 kDa) can be a limitation. Altogether, self-labeling protein tags have been successfully applied to labeling proteins with small-molecule fluorophores for localization and trafficking studies. Although their size is still large with respect to possible aberrant effects on the function of the target protein of interest, the photochemical properties of the organic fluorophores that can be attached to proteins in this way are superior to those of genetically encoded fluorescent proteins.

Another recent method to label proteins with high specificity and efficiency involves the use of the SpyTag/SpyCatcher system (Figure 4c). This technique is based on the fibronectin-binding protein (FbaB) of *Streptococcus pyogenes*, which is split into two parts.^305^ The first short segment (SpyTag, 13 AA-long) is fused to the POI and reacts with high specificity with the second fragment (SpyCatcher, 133 AA, ∼12 kDa), which can be fused to a fluorescent tag, through the formation of a covalent isopeptide bond.^306^ While the efficiency of the reaction is very high, the total molecular weight added to the POI is quite substantial. Furthermore, the method is applicable only to the protein termini.

A strategy that couples the small size of the peptide hairpins with the specificity of enzymatic labeling is the use of short peptide tags that are substrates of enzymes, whose activity is engineered so as to react with desired substrates (Figure 4d). For instance, biotin-ligase (BirA)^307^ has been employed to equip proteins with biotin tags and modified variants of phosphopantetheinyl-transferase (PPTase) from bacteria^308^ to equip proteins with fluorophores. These enzymes do not react in mammalian cells with the corresponding substrates. Lipoic acid ligase (LplA)^309^ and sortase A (SrtA)^310^ have also been employed in a similar manner to label proteins with small fluorophores. As with other enzyme-mediated labeling techniques, the peptide to be installed in the protein of interest can vary in length from between 5- to 15-AA residues, and, in some cases - as for SrtA - can only be appended to specific regions of the protein (Figure 4d).

Additionally, a labeling method based on template-recognition was achieved by employing coiled-coils motifs. According to this approach, a peptide sequence is incorporated into the chosen protein. A complementary peptide carrying a fluorophore that binds to the sequence is externally added to the system (Figure 4e). The dye transfers to the protein via an acyl-transfer reaction mediated by a Cys residue at the N-terminus. The reaction is quite specific, as it is promoted by the proximity between the two reactive groups induced by the coiled-coil interaction.^311−313^ The peptide sequences required for optimal labeling efficiency consist of approximately ∼12–22 AA residues. While this tag is much smaller than a FP, its applicability is still limited, since the sequence can only be incorporated at the N-terminus.

So-called self-labeling tags (Figure 4f) were designed to couple a fluorophore to a protein of interest in a designated position. The smallest among these tags is the tetracysteine tag CysCys-X-X-CysCys, which forms a hairpin motive and allows for binding fluorescent dyes coupled to a biarsenical moiety, for a total size of ∼1–2 kDa. Two of those tags have been used: the fluorescein arsenical hairpin binder-ethanedithiol (FlAsH) and red-fluorescent arsenical hairpin binder-ethanedithiol (ReAsH).^254,314,315^ An optimized peptide motif allows labeling of intracellular proteins in live mammalian cells with moderate specificity^316^ and has been applied to protein localization studies and for intracellular binding experiments using FRET.^317,318^ The rhodamine-based bisboronic acid (RhoBo) tag follows the same principle as FlAsH and ReAsH, but instead of targeting Cys residues, it binds to Ser residues and does not require toxic reagents, although it might give rise to nonspecific labeling of proteins with tetra-Ser sequences.^319^ Similarly, a Cys-oligo-Asp sequence was inserted in a protein of interest and labeled with a Zn(II)-fluorophore.^320^

#### 2.3.3 Minimal-Sized Labels

The smallest possible tags that can be used to label a protein are attached on side chains of single residues. One special strategy to label single residues of a protein, which can be applied for instance to receptors, is ligand-directed labeling. For example, a fluorophore bound to an electrophilic group can be linked to a high-affinity ligand of choice. Upon binding of the ligand to the receptor, the electrophilic group can react with nucleophiles in the surroundings of the binding pocket and label the protein with the fluorophore.^321,322^

Another strategy involves the direct targeting of endogenous AAs that harbor reactive groups (such as Cys and Lys). While the post-translational labeling of Cys residues with succinimidyl-esters has been extensively used and is still used especially in NMR and EPR studies, incorporation of an extra Cys can affect the protein structure, as it can interfere with protein folding. Additionally, labeling of Cys requires reducing agents, which is not always possible in live cells or for certain proteins. In general, the labeling of endogenous AA residues is not position specific (unless only one AA of that type is present in the protein) and gives rise to heterogeneously labeled populations. This calls for the need to substitute all the residues of a certain type that should not be labeled with alternative AAs, a procedure that introduces a major modification into the native protein. This approach is obviously not feasible in live cells, as it lacks specificity.

The use of GCE to incorporate an ncAA into the protein of interest provides an attractive alternative to other techniques of protein labeling. The target protein needs to be modified at a single residue, which will be substituted with the ncAAs. In this way, there is less concern over correct folding or activity of the protein than in regular mutagenesis experiments. Although some small fluorophores can be incorporated synthetically to produce ncAAs with fluorescent side chains, and then used for GCE, their photochemical properties are not good enough for many high-end microscopy experiments. Therefore, the preferred method is to incorporate click-ncAAs that can be later labeled using bioorthogonal chemistry with a fluorescent dye or a probe of choice. The labeling happens post-translationally, yet in an exquisitely chemo-specific fashion, so that the method can well be applied directly in living cells. The size of the small-molecule dye is much smaller not only than that of a fluorescent protein (∼1 kDa compared with ∼27 kDa for GFP) but also than the size of self-labeling tags and thus less likely to impair protein function. Importantly, the availability of satisfactory GCE methodologies and bioorthogonal chemistries has also led to the development of scores of fluorescent dyes modified with click-compatible functional groups, which are largely commercially available, so that the method is easily accessible for general usage. Reviews on the application of ncAAs for cellular imaging are available in the literature.^323^

## 3 GCE to Address Questions about Membrane Proteins

Membrane proteins are crucial components for the functioning of cells. A general feature of membrane proteins is the presence of hydrophobic domains that span through the phospholipid bilayer. To keep membrane proteins native and functional, they must be embedded either in the natural membrane or in artificial lipidic systems (e.g., lipid vesicles, detergent-lipid bicelles, detergent micelles or membrane nanoparticles), which in general complicates the handling of these proteins compared to soluble cytosolic proteins.

Despite significant technical advances in crystallography and especially cryogenic electron microscopy (cryo-EM) as well as the allied molecular and biochemical methods such as heterologous overexpression, protein engineering and purification, the high-resolution structure determination of some classes of membrane proteins remains challenging. For example, given their interest as drug targets, structures of G protein-coupled receptors (GPCRs) have been a focus of intense efforts over the past two decades. Still, only discrete complex structures for about 100 GPCRs among the 350 or so ligand-activated nonolfactory GPCRs encoded in the human genome are now available. The allied use of high-performance computational molecular dynamics (MD) modeling has led to a new era of so-called “rational drug design”. In any case, as high-resolution structures are derived from reconstituted proteins and protein complexes in a highly artificial environment, they can greatly benefit from complementary information derived from biochemical methods in cells. For example, large scale cross-linking data sets generated via GCE-incorporated cross-linkers^85,242^ have helped provide structural information about flexible regions of GPCR-arrestin complexes that are not resolved in 3D structures and provide insights about structural hallmarks directly in the live cell environment.

GPCRs, and membrane proteins in general, are highly dynamic and are known to shift between different metastable conformations in the transition from inactive state to active state. The structural dynamics of protein function cannot be addressed by structural snapshots alone. Although MD in combination with high-resolution active-state and inactive-state 3D structures provides evidence for dynamic changes concomitant with receptor activation, experimental methods such as NMR, EPR and biochemical spectroscopy are the methods of choice to study protein dynamics, including GPCRs and membrane proteins, in real time and even in the live cell.

A variety of single molecule (sm) techniques have been employed to study membrane protein dynamics. Information obtained from sm studies can be complementary with the static conformational information obtained from X-ray and cryo-EM structures.^6^ sm studies, compared to ensemble spectroscopic techniques, offer the advantage of discriminating among different endogenous populations and singling out a specific molecule, with the possibility of following a conformational read-out through time. The fusion of single molecule spectroscopy and microscopic imaging has provided a very sensitive technique that allows for the observation of single fluorophores with nanometer precision. An important requirement for sm microscopy is a small, bright, stable and easily linkable fluorophore – a requirement often best addressed using GCE and bioorthogonal labeling.^324^ Following a specific fluorescence signature through time can provide also insight into diffusion patterns and kinetics of the binding reactions and has also been used for stoichiometric analysis of complexes,^325^ as well as to monitor changes in local chemical environments (through changes in fluorescence lifetime) induced either by conformational rearrangements or interaction partners. This application has produced particularly interesting results in smFRET studies.

Membrane proteins are in general difficult targets for GCE. They are synthesized by ribosomes bound to the rough endoplasmic reticulum and are cotranslationally translocated to the ER lumen. Probably because of this difference in the mechanism of biosynthesis, membrane proteins give in our experience lower ncAA incorporation yields compared to soluble proteins, which are assembled in the cytosol. It is possible that missed amber suppression at the translocon leads to ribosome stalling and degradation of the nascent protein to a higher extent than happens in the cytosol. In this context, aspects related to membrane protein folding and misfolding can also play a role. Notably, dedicated GCE systems were developed to introduce ncAAs in GPCRs expressed in mammalian cells in culture. The first systems for GCE applied to GPCRs focused on increasing the tRNA expression level by using a dedicated plasmid carrying multiple tRNA expression cassettes^14^ or by embedding multiple tRNA-expression cassettes in one-plasmid vectors.^326^ Further efforts have been dedicated to establishing the most efficient Pol III promoter for tRNA expression^51^ and to enhancing the efficiency of the tRNA itself.^17,51^ In general, to achieve good ncAA incorporation into membrane proteins, it is essential to employ highly efficient GCE systems, as even moderate improvements in GCE efficiency can have a great impact on the expression membrane proteins.^327^

In the sections below, we summarize examples of how ncAAs have facilitated membrane protein studies in the context of GCE. We describe applications of ncAAs to study structural aspects and structural dynamics of membrane proteins, their localization and oligomerization, and the interactions with their ligands and with intracellular proteins. Membrane proteins to which GCE has been applied include ion channels (for instance voltage gated channels), GPCRs (rhodopsin, hormone GPCRs, metabotropic glutamate receptors), as well as other receptors and membrane-embedded enzymes (e.g., EGFR, IL-4R, ATPases). We will also include proof-of-principle demonstrations of GCE methods that have not yet been applied to membrane proteins but that could be fruitfully applied in the future.

### 3.1 Structure and Structural Dynamics

#### 3.1.1 X-ray Scattering

**Figure 5:**
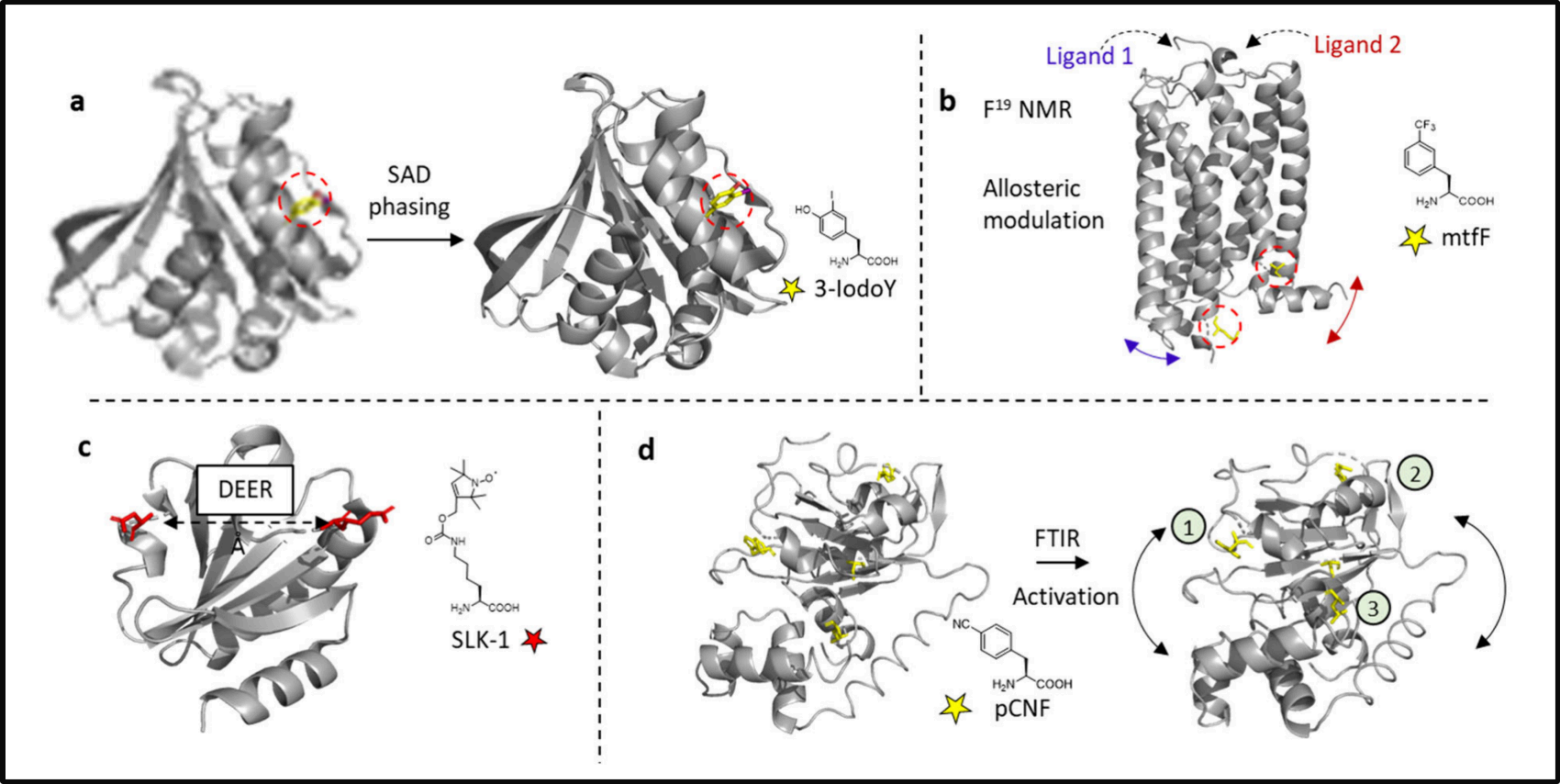
Applications of GCE for structural and structural dynamics studies. (a) Resolution of the N-acetyltransferase structure (PDBID: 2Z11) by SAD phasing with an iodine AA (3-iodoY **35**). (b) Fluorinated ncAA (mtfF **16**) introduced into cannabinoid receptor CB1 (PDBID: 5TGZ) enables the measurement of allosteric ligand-induced conformational changes using NMR. (c) Measurement of distance between two positions in thioredoxin (PDBID: 2TRX) using DEER spectroscopy with radical amino acid (SLK-1 **31**). (d) Incorporated ncAA (pCNF **34**) is used to record sequential conformational changes in light-oxygen-voltage domain (PDBID: 3P7N) using FT-IR spectroscopy after protein activation. For all figures, the ncAA structure is not in scale with the shown proteins. Figures showing a cell culture dish refer to studies conducted in live mammalian cells.

NcAAs carrying heavy atoms can facilitate X-ray crystallography. For example, *p-*iodo-l-phenylalanine (pIodoF **36)** has been incorporated in bacteriophage T4 lysozyme and human superoxidismutase (hSOD) into *E. coli* and *S. cerevisiae*, respectively, and the iodine atom was used to facilitate the phasing of data from X-ray diffraction^10,46,128^ (Figure 5a). Just by the introduction of one iodine atom in the structure, it was possible to carry out single-wavelength anomalous dispersion (SAD) phasing and solve the structure of T4 lysozyme.^128^ Similarly, the crystal structure of the ribosomal protein N-acetyltransferase from *Thermus thermophilus* incorporating **36** was determined with good resolution.^328^ 2-Amino-3-(8-hydroxyquinolin-3-yl)propanoic acid (HQ-Ala **37**) has a metal-ion chelating group that has been exploited for SAD phasing for structure determination by adding Zn^2+^ to a mutant of O-acetylserine sulfhydrylase protein from *Thermotoga maritima* expressed in *E. coli*.^130^ To our knowledge, no incorporation of ncAAs for scattering experiments has been done in membrane proteins.

#### 3.1.2 NMR and EPR

In NMR applications, the introduction of atoms with nuclear spin via ncAA-mutagenesis can improve the ability to interpret spectra, especially in transverse relaxation-optimized spectroscopy (TROSY) experiments. The most advantageous feature of GCE is the possibility of labeling one specific residue of the protein. Although this has been achieved by cell-free expression systems with chemically aminoacylated suppressor tRNAs,^233^ it becomes difficult to produce large quantities of a protein for NMR measurements this way. In some cases, these limitations have been overcome with excellent results. A good example is the *in vivo* incorporation of ^15^N- labeled *p*-methoxyphenylalanine (^15^N-pOMeF **11**) into sperm whale myoglobin in *E. coli*.^107^ However, no application of GCE in combination with NMR, or EPR, has been reported so far for membrane proteins.

NMR probes can also be applied as environmental sensors and monitor conformational changes. The ncAA *p*-trifluoromethylphenylalanine (tfmF **19**), a probe for fluorine NMR, was used to measure solvent exposure as an indicator of membrane topology in diacylglycerol kinase (DAGK) in *E. coli*.^329,330^ The chemical shifts of the environmentally sensitive fluorides were associated with buried and exposed positions in the topological analysis. The same TfmF **19** was incorporated into nitroreductase (NTR) and histidinol dehydrogenase (HDH) in *E. coli* and the chemical shift of ^19^F was used to monitor the structural changes of proteins homodimers upon binding of inhibitors to the proteins.^117^ Likewise, ^13^C-*p*-methoxyphenylalanine **12** was also used in NMR studies on the *Sulfolobus solfataricus* cytochrome P450 CYP119, helping in the identification of two conformational states of the protein upon ligand binding.^108^ Additionally, mtfF **16** was used to identify preactive states in the binding of allosteric modulators to the cannabinoid receptor^113^ (Figure 5b).

Radical probes for EPR can also be incorporated via GCE. Nitroxide-Lys (SLK-1 **31**) was first incorporated in GFP in *E. coli*(94) and then applied to thioredoxin (TRX) to report intramolecular distances associated with conformational changes in the enzyme^93^ in live cells, although the stability of this ncAA is not optimal^331^ (Figure 5c). Recently, the tetrazine- AA Tet-v4.0 **137** was incorporated in multiple positions of the model maltose-binding protein (MBP) and clicked to a TCO-nitroxide moiety to measure the distance between the residues by double electron–electron resonance (DEER) spectroscopy. Furthermore, the labeling with TCO-nitroxides was efficiently demonstrated in live mammalian cells with membrane-permeable derivatives, and intracellular labeling of double GFP mutants was achieved, allowing the possibility of measuring EPR signals from intact cells,^202^ a result that will certainly advance the field of live-cell EPR studies of protein dynamics. pAcF **118** was incorporated in T4 lysozyme at a solvent exposed helix site, and the AA was labeled with hydroxylamine reagent, yielding a nitroxide moiety. The protein, incorporating two of these labels, was then analyzed with DEER and the distance measured as proof-of-principle of the technique in distance mapping.^332^

#### 3.1.3 FTIR

The azido and cyano groups feature IR vibrational fingerprints that are not found in endogenous cellular proteins and can be used as local conformational and environmental probes. The strategy of introducing such IR probes site-specifically using GCE is particularly useful especially when combined with experimental methods that taking advantage of IR-difference spectroscopy. For example, pAziF **32** and *p*CNF **34** were introduced in calmodulin (CaM) and used to measure solvent sensitivity to conformational rearrangements.^333,334^ The stretching vibrational frequencies of the groups in the engineered proteins changed after binding of the ligand and thus served as conformational sensors.^334^

FTIR-difference spectroscopy was done over the years in the probing of protein dynamics and conformational changes. An outstanding example of application of pAziF as an IR probe in membrane protein is a study on the activation mechanism of rhodopsin. First Fourier-transform infrared (FTIR) difference spectroscopy studies using Asp to Asn and Glu to Gln single- and double-replacement mutants of rhodopsin expressed in mammalian cells in culture showed the role of a proton transfer reaction in releasing structural constraints for the transition of the receptor into the active conformation.^335,336^ Upon establishing a GCE system to introduce ncAAs in GPCRs,^14^ pAziF was site-specifically introduced at various sites as local polarity probes specifically for FTIR studies,^235^ which determined the precise onset of crucial transmembrane (TM) helical movements that could be correlated with the metarhodopsin I state and its conversion to the active metarhodopsin II state.^236^

Two-dimensional IR can also reveal dynamics of interactions. pAziF was incorporated into myoglobin and used as a sensor in ultrafast 2D-IR to monitor protein motions for ligand-free and CO-bound myoglobin; in this case, pAziF allowed the study of dynamics in oxidized myoglobin, which had not been achieved before for 2D-IR.^337^ Ultrafast IR spectroscopy with pAziF was also able to give information about the dynamics of the BLUF domain flavoprotein (blue-light using flavin) structural changes after activation using light.^338^ Signal transduction of *Deinococcus radiodurans* bacteriophytochrome was tracked in a time-resolved manner by monitoring the vibrational stretching of pAziF by FTIR after activation with light. The kinetics of the process revealed a very fast responding region in the chromophore, called the tongue-region.^339^ The kinetics of activation of the light-oxygen-voltage were studied instead by incorporation of *p*-cyanophenylalanine (pCNF **34**) (Figure 5d) for UV–vis and IR spectroscopy^340^ and the same AA was also used in Bathy Phytochrome Agp2 to study its electric field changes during photoconversion, showing that the biggest changes of the nitrile group stretching vibration happen at the last step of photoconversion.^341^

#### 3.1.4 Fluorescence Microscopy

##### 3.1.4.1 Microscopy with Fluorescent ncAAs

**Figure 6:**
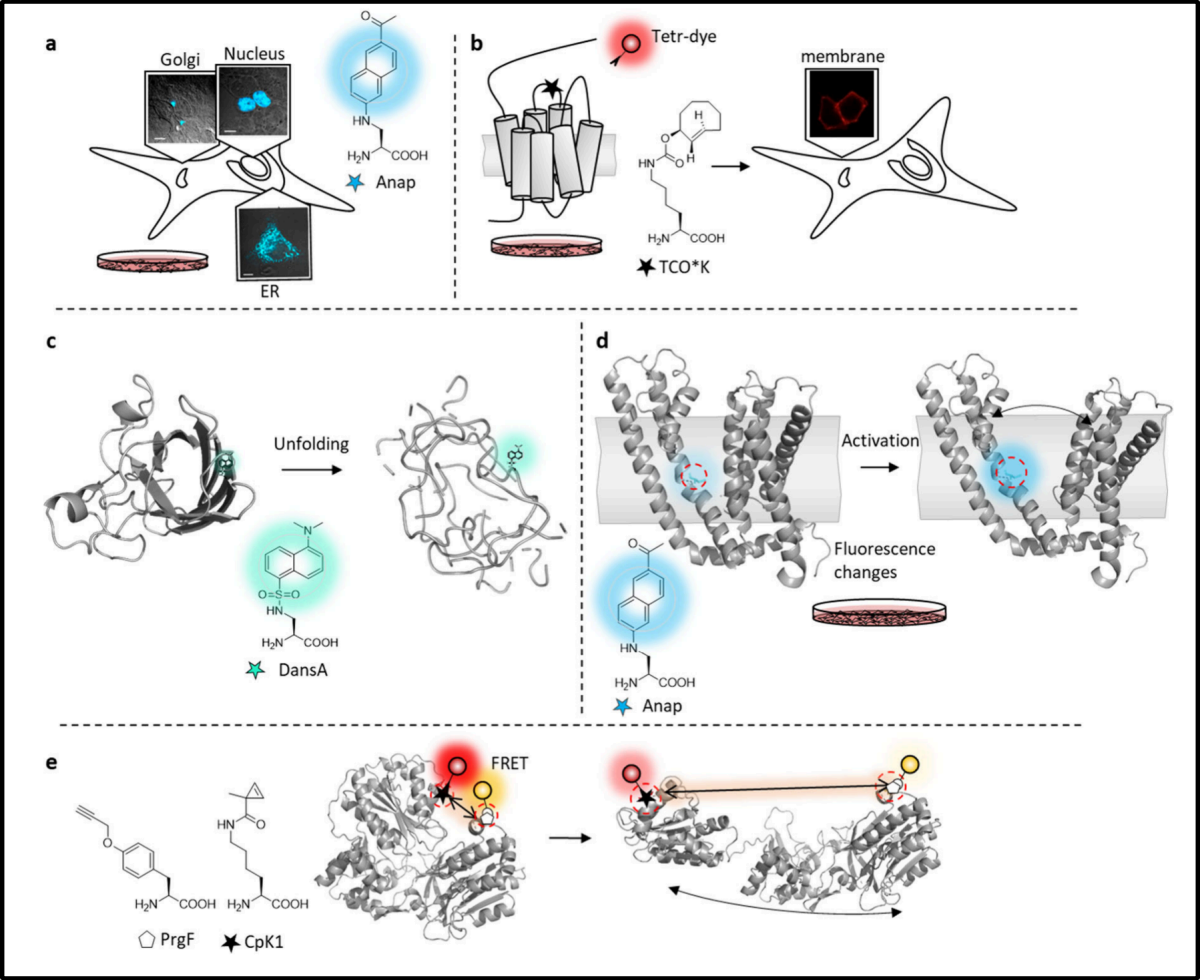
Employing fluorescence microscopy for structural and structural dynamics studies. (a) A fluorescent ncAA (Anap **5**) is genetically incorporated into a protein to track its cellular localization in live cells. The microscopy images are taken from J. Am. Chem. Soc.2013, 135, 12540–1254323924161 10.1021/ja4059553PMC3783214.^344^ (b) A bioorthogonal handle ncAA (TCO*K **143**) is incorporated into membrane protein and subsequently labeled using click chemistry with a fluorescent dye (Tetr-dye) enabling visualization of its localization on the plasma membrane in live cells. The microscopy image is taken from ACS Chem. Biol.2019, 14, 1141–114931074969 10.1021/acschembio.8b01115.^346^ (c) Monitoring the unfolding of superoxide dismutase (PDBID: 1PU0) using an environmentally sensitive AA (DansA **1**). (d) An environmentally sensitive fluorescent ncAA (Anap **5**) is used to monitor the activation of channel proteins in live cells (PDBID: 3J5P). (e) Two bioorthogonal handle ncAAs (pPrgF **114** and CpK1 **138**) introduced in cytochrome P450 reductase (CPR) (PDBID: 5FA6 and 3FJO) are labeled with fluorophores using click chemistry. FRET is measured to evaluate conformational rearrangement of the protein. For all figures, the ncAA structure is not in scale with the shown proteins. Figures showing a cell culture dish refer to studies conducted in live mammalian cells.

Fluorescent ncAAs have found application both for the study of protein structure and in localization studies of membrane proteins (Figure 6a, b). Fluorescent AAs find their most obvious application to localization studies. The coumarin AA **2** was used to label bacterial tubulin homologue FtsZ^342^ and the molecular chaperone GroEL^343^ for *in vivo* subcellular localization studies in *E. coli.* Later, Anap **5** was used for live-cell imaging of the subcellular localization of different proteins in mammalian cells.^344^ Histone H3, Grp94 and GalT1 were correctly colocalized to the nucleus, ER and Golgi, respectively. The incorporation of Anap at different positions on the human cardiac voltage-gated sodium channel (Na_v_1.5) was also employed in intact mammalian cells to map the environmental hydrophobicity in both inactivated and activated channel conformations. Acridonylalanine (Acd **6)**, which carries a fluorophore with improved photostability, was used as a reporter of membrane and cytosolic protein localization in mammalian cells for the insulin receptor (IR), hyperpolarization-activated cyclic nucleotide–gated ion channel (spHCN) and the maltose binding protein (MBP). Additionally, due to its long lifetime, it was used for FLIM imaging to reduce the background signal of intracellular Acd.^103^ The sulfur-analogue of Acd, thioacridonylalanine (SAcd **7**), which is photoactive, was incorporated in proteins in *E. coli* and was used as fluorescent probe, expanding the category of “turn-on” probes for high-end imaging techniques.^104^ Acd **6** was also used to monitor conformational changes in CaM induced by peptide binding *in vitro*, showing the shift in the local environment of specific positions.^102,345^

Since fluorophores are environmentally sensitive, fluorescent ncAAs can be applied as environmental probes or as sensors to monitor local conformational changes in the protein structure, thus providing both structural and dynamic information. AlaDAN **4** was first incorporated into both soluble (GB1, IgG-binding domain) and membrane proteins (Kir2.1 and Shaker potassium channels) under an amber stop codon after injection of AlaDAN-charged suppressor tRNA in *Xenopus* oocytes.^100^ The shift of AlaDAN excitation peaks was registered for differentially buried positions in the protein, therefore confirming its dielectric-sensitivity and allowing the mapping of hydrophobicity and solvent exposure in the protein core. The fluorescence intensity changes of coumarin have also been used to monitor folding of cytosolic proteins. In the case of sperm whale myoglobin, denaturation of the protein with urea was accurately followed *in vitro*.^98^ Unfolding of hSOD was also monitored with DansylAla **1** in yeast, and the change of fluorescence identified a position-specific shift in local polarity^49^ (Figure 6c). Conformational changes were also monitored in the voltage-sensitive domain of *Ciona intestinalis* voltage-sensitive phosphatase (CiVSP) in neurons by incorporation of DansylAla **1**. The results showed opposite changes in fluorescence from distal positions on the S4 domain, suggesting a corresponding opposite change in environmental polarity due to polarization.^226^

Similarly, Anap **5** has been used to gain insight into ligand-induced folding and conformational rearrangements of cytosolic proteins, such as hSOD and QBP in bacteria and firefly luciferase in yeast,^101,347^ and membrane proteins, especially ion channels, in *Xenopus* oocytes and mammalian cells. Protein channels incorporating Anap in buried positions were studied by voltage clamp fluorimetry. Anap allowed the analysis of the dynamics of opening and closing of voltage-gate potassium channels (Kv) at key regions that are otherwise inaccessible with Cys labeling. Applications on the Shaker voltage-gated potassium channel in *Xenopus* oocytes have shown sequential movements of the protein.^348^ Similarly, Anap was used in the study of the capsaicin receptor TRPV1 in mammalian cells, where it reported on the shift between apo- and ligand-bound forms.^349^ The conformational rearrangements of the transient-receptor potential melastatin 8 (TRPM8) ion channel were also observed with Anap for the binding of menthol in live mammalian cells, revealing the opening of the S6 bundle crossing gate and changes near the selectivity filter.^350^ Patch-clamp fluorometry on the voltage-gated proton channels (H_V_ 1) incorporating Anap in crucial positions have also revealed the movement of the S4 segment in response to a change in pH. In this case, quenching of Anap by an aromatic residue in the S2 helix was seen (Figure 6d). Additionally, a slow transition in the deactivation pathway was observed when measuring the kinetics of the fluorescence change.^351^

Anap has additionally served as a donor to create fluorescence donor–acceptor pairs for FRET measurements in mammalian cells. First, it was paired to GFP to monitor Ca^2+^ dependent conformational changes of Calmodulin bound to peptide M13.^352^ Then, it was paired to copper in a novel technique, termed ACCuRET (Anap Cyclen-Cu^2+^ Resonance Energy Transfer), to monitor the protein rearrangements in native membranes and measure absolute distances between two residues. This was first shown as a proof-of-principle by introducing Anap on maltose-binding protein (MBP) and then by clicking the acceptor Cu^2+^, bound to (1-(2-pyridin-2yldisulfanyl)ethyl)-1,4,7,10-tetraazacyclododecane (TETAC), on a free Cys residue.^229^ One notable application of this technique in the study of membrane proteins involved the equipping of the TRPV1 ion channel with Anap, the anchoring of Co^2+^ ions to the cell membrane via a metal chelating lipid and the measuring of the distance between the specific ion channel domains and the cell membrane through tmFRET.^353^ Coumarin-ncAA **2** was also used for FRET in combination with a FlAsH-tag to generate a sensor for studying the dynamics of G protein coupling to a GPCR by fluorescence lifetime analysis in mammalian cells. In this way, the reciprocal movement of two segments of the G protein was monitored during activation.^354^ Additionally, Acd **6** was incorporated in the MBP in mammalian cells using the PylRS system for time-resolved tmFRET in combination with Cu^2+^ ions to probe the distribution of conformational states of apo and holo MBP.^355^

##### 3.1.4.2 Microscopy with Clicked Fluorophores

The genetic incorporation of ncAAs carrying anchors for bioorthogonal chemistry allows equipping proteins post-translationally with bright and photostable fluorophores, allowing for high-end microscopy techniques that are not practical on genetically encoded fluorophores. In an early study reporting ncAA-mediated post-translational protein labeling, *m-* and *p*-acetylphenylalanine (**118** and **119**) were incorporated in the membrane protein LamB^190^ in *E. coli*. Similarly, pPrgF **114** and pAziF **32** have been incorporated in proteins and labeled with fluorophores through CuAAC or Staudinger ligation^125,187^ both in yeast and bacteria. The monitoring of localization and trafficking of the IFITM3 protein, a small membrane-associated protein, was also achieved with GCE by incorporating TCOK **142** and labeling with membrane-impermeable tetrazine dyes in live mammalian cells.^356^ In a follow-up study, labeling of IFiTM 1,2 and 3 helped identify the important role of these proteins in the prevention of infections by viruses. The labeled proteins were observed after stimulation of the cells with interferon, and the IFITM3 was imaged during the process of viral particle transport to the lysosomes.^357^

Clickable dyes have been very important in sm tracking and localization microscopy to measure the spatial distribution and movement on the cell membrane. The epidermal growth factor receptor (EGFR) and the potassium channel Kv were both studied in tracking and localization experiments, by incorporation of BCNK **146** and labeling with tetrazine dyes, showing that these proteins are homogeneously distributed through the cell membrane of mammalian cells and are not located in subdomains.^358^ In a recent study, bioorthogonal labeling of low abundance receptors has been achieved on the polytopic glucagon receptor (GCGR) by labeling BCNK with a tetrazole-dye and irradiation with UV light. This labeling reaction was performed in live mammalian cells and has the fastest rate reported for BCN labeling, is very specific and could be applied for spatiotemporal studies of membrane protein populations.^359^ GCE has been used also in the labeling of intracellular positions in proteins and membrane receptors. Intracellular labeling of transcription factor JunB, β-actin and vimentin proteins, carrying BCNK, was performed with tetrazine dyes and visualized with wide-field or super-resolution microscopy, showing specific labeling^208^ and revealing nuclear actin filaments^360^ in mammalian cells. Intracellular labeling in the third intracellular loop (ICL3) of the GCGR was achieved in mammalian cells by clicking membrane-permeable dyes to strained alkene-containing NcAAs **143** and **147**. Unfortunately, the high intracellular background signal of the dye hampered the possibility of applying this technique for signaling studies and ICL3 movements.^361^

Recently, the difference in labeling efficiencies in several positions on proteins bearing ncAAs was closely examined and correlated to protein structure. The interleukin-4 receptor alpha (IL-4Rα), bearing BCNK **146** and labeled with tetrazine-tagged fluorescent dyes, was expressed in mammalian cells and analyzed directly in the live cells by confocal microscopy to estimate the labeling yield for each analyzed position. Computational analysis of solvent polarization for each residue helped rationalize the differences in labeling yields and correlate them with small deformations of the receptor structure in the native system.^362^ Moreover, the *N*-methyl-d-aspartate (NMDA) receptor was modified to incorporate TCOK at extracellular positions and was labeled with tetrazine dyes in mammalian cells for super-resolution microscopy experiments. The results showed a better labeling efficiency than immunostaining and full functionality of the receptor, demonstrating that the strategy can be minimally invasive for studies of subunit composition when applied to multiple ncAAs.^363^

An important application of clicking bioorthogonal dye residues is the potential for FRET studies. Proof-of-principle experiments consisted of incorporating pBpa **42** into the T4 lysozyme in bacteria. By clicking a hydroxylamine-dye on pBpa and a maleimide-dye to a free Cys in the protein, dual-color labeling was achieved. Furthermore, smFRET was applied and the signal was monitored during folding/unfolding of the protein^364^*in vitro*, demonstrating the clear advantages of click-chemistry. In a similar study, dual labeling of CaM via CuAAC and maleimide reactions was done for FRET studies of the protein’s conformational rearrangements upon ligand binding.^60^ This concept was applied later to the study of GPCRs after the optimization of GCE tools.

Incorporation of pAziF **32** in the ghrelin receptor and labeling via SPAAC was carried out to perform FRET studies with labeled ligands to monitor ligand-induced conformational rearrangements in the receptor^365^ in mammalian cells. Similarly, the ghrelin receptor GHS-R1a was also expressed and purified, bearing pAziF in the intracellular side of the receptor. Upon labeling with alkyne-dyes, the LRET between the receptor and a G protein, linked to the fluorophore Lumi4-Tb, was measured. The results showed that the ghrelin receptor is a dynamic protein that shifts between conformations in the basal state. Agonists stabilize the active conformation with the G protein in a specific state, while antagonists stabilize an additional inactive conformation, different from the basal state and lacking all receptor-G protein interaction.^366^ Similarly, labeling of class B GPCRs (GCGR and glucagon-like peptide-1 receptor) was conducted by clicking tetrazine dyes to SphK **147** in the protein of interest in mammalian cells; characterization of these receptors showed full functionality, making it a satisfactory starting point for future dynamic studies.^209^ The activation mechanism of the EGFR was studied by incorporation of a FRET sensor (named CONEGI) including an ncAA tagged with a fluorescent dye in the tyrosine-kinase domain of the EGFR coupled to a FPs in a nonvariable region in mammalian cells. This sensor was able to report conformational changes in the functional domain of the receptor, thereby elucidating the mechanism of activation.^367^ A similar strategy was used to apply dual fluorescent labels to the amyloid-β protein precursor, a membrane protein involved in Alzheimer’s disease pathogenesis, to allow analysis of protein processing and transport in live cells in culture. One label was applied using a SNAP tag, and the second was applied at ten different genetically encoded TCOK residues.^368^

The possibility of incorporating two ncAAs into proteins has allowed the direct labeling with FRET partners in both *E. coli* and mammalian cells. The human NADPH-cytochrome P450 reductase (CPR) was expressed in bacteria bearing pPrgF **114** and CpK1 **138** under amber and ochre stop codons and was later labeled via CuAAC and SPIEDAC with the known Cy3-Cy5 FRET pair. SmFRET measurements revealed the effect of NADP^+^ in the modulation of the CPR conformation^369^ (Figure 6e). In a similar study, pAziF and tetF **134** were incorporated in yeast Replication Protein A (RPA) and the FRET signal was measured between the subunits of the protein as a function of ssDNA concentration.^370^ Incorporation of two ncAAs (TCOK **142** and pPrgF) has also been demonstrated in live mammalian cells for the Notch receptor and corticotropin-releasing factor receptor type 1 receptor (CRF_1_R), where labeling was achieved with tetrazine and azide dyes, respectively.^72^

Application of GCE to the metabotropic glutamate receptor 2 (mGluR2) allowed for live FRET measurements in mammalian cells and found two new intermediate conformational states of the receptor upon activation. The receptor, which is naturally dimeric, was expressed incorporating pAziF and was labeled with a mixture of Cy3 and Cy5 alkyne dyes; the FRET signal arising from the combination of the two in the same dimer was registered.^371^ Furthermore, the α-amino-3-hydroxy-5-methyl-4-isoxazolepropionic acid receptor (AMPAR) was expressed in neurons with an ncAA (TCOK) and labeled with tetrazine dyes to install a FRET pair in combination with a SNAP-tag in one of the subunits. The FRET signal was measured upon activation of the receptor. Furthermore, the different distribution of these subunits was observed in neurons and the possibility of labeling masked epitopes was unlocked.^372^

The bioorthogonal labeling of adhesion GPCRs was also shown by clicking tetrazine dyes to TCOK into ADGRB3, ADGRE2, ADGRE5, ADGRG1, and ADGRL1 receptors in mammalian cells. The formation of a heterodimer at the cell surface between the N- and C-terminal fragments, generated after proteolysis, was demonstrated by pairing the dyes with SNAP-tag labeling inside the protein. Intra- and intermolecular FRET signals were observed, demonstrating the presence of both cleaved structures at the cell surface.^373^ Besides FRET, ncAAs and click-labeling have been applied for BRET studies. A ncAA was introduced in ECL3 of the frizzled receptor FZD-CRD, bearing a nanoluciferase (Nluc) at the N-terminus. By clicking the orthogonal dye on the receptor, the BRET signal was registered in mammalian cells upon binding of endogenous ligands and conformational rearrangements of the receptor were compared between the different ligands, showing surprising similarity despite the distinct activation pathways they promote.^374,375^

#### 3.1.5 Cross-linkers

Information about protein structure and conformational rearrangements can also be derived through the site-specific incorporation of cross-linking ncAAs, as has been demonstrated, for instance, in studies on ion channels. The ionotropic glutamate receptor AMPAR was studied by incorporation of pAziF and pBpa into transmembrane domains in the channel gating region. Results showed a difference in ion current depending on the cross-linked position, providing insight into the gating mechanism.^376^ In other studies, pAziF was incorporated into the light-sensitive ionotropic glutamate receptor (NMDAR). In these cases, photo-cross-linking hits identified negative and positive allosteric modulation of this receptor, shedding light on subtype-specific receptor interfaces.^377,378^ In a following study, photo-cross-linking allowed for the differentiation of the ligand binding sites and subtype-specific allosteric pathways in NMDAR activation.^379^ pBpa was also incorporated into the linker region of acid-sensing ion channel ASIC1, and the conformational rearrangement of this area was characterized upon activation of the channel.^380^ Additionally, an extensive application of three cross-linkers pAziF, pBpa and AbK to yield more than 300 ASIC1 receptor variants showed that the acidic pocket of the channel is the main determinant of current decay.^381^ In a recent study, pAziF and pBpa were incorporated into the calcium ion channel Orai1 at the plasma membrane, and UV light was used to trigger channel activation even in the absence of its endogenous activator. The downstream signaling of the receptor in this case mimicked the one observed for wt Orai1 in the presence of endogenous ligand. This was done to investigate the dynamics of TM rearrangements in the opening-closing mechanism of the channel and allowed for the monitoring of changes in channel activity.^382^ Similarly, the activation of the acid-sensing ion channel (ASIC) was investigated by incorporation of pAziF and pBpa into the acid pocket, showing the relevance of distinct positions in prevention of pocket collapse.^383^ pAziF and pBpa have also been applied to structure–function relationship studies on ligand-gated ion channels in *Xenopus laevis* oocytes.^384^

As another example of application, the architecture of the Tom40 subunit, a mitochondrial protein translocation complex, was investigated using pBpa **42** cross-linking and the active form of the protein was mapped to reveal several transport paths for preproteins.^385^ The protein-conducting channel for the secretion of misfolded proteins in the ER via the ER-associated protein degradation (ERAD) system was also identified with cross-linking studies employing pBpa on the Der1 protein.^386^ Cross-linking with Bpa **42** has been combined with mass spectrometry for the study of the carboxyl-terminal regulatory domain of proton-pumping ATPase AHA2 in yeast. The results showed a direct interaction of this domain with the N-terminal actuator domain, spurring the name “head-to-tail” organization of ATPases with each other.^387^ Another study on ATPase was conducted with pBpa to define the role of subunit ε in enzyme regulation of activity. Two states were found, the “inserted” and “non-inserted”, which are in equilibrium and contribute to keeping a background level of ATP synthesis for the switching between the two states.^388^

Information on structural hallmarks can also be indirectly derived from protein-protein interactions studies (described below). For instance, when studying the interaction of the CRF_1_R GPCR with its Urocortin ligand via systematic photo-cross-linking mapping of the receptor, photo-cross-linking hits at a regular distance of 3 or 4 amino acids revealed the helical structure of receptor segments.^51^ In other protein-protein interaction studies, photo-cross-linking hits at every second amino acid confirmed instead a β-strand conformation.^389,390^ On the other hand, both photo- and chemical cross-linking can report on intramolecular proximity between different parts of a protein, as has been shown, for instance, in cross-linking studies on arrestin.^390^

### 3.2 Studies of Protein-Ligand and Protein-Protein Interactions

#### 3.2.1 NMR and EPR

**Figure 7:**
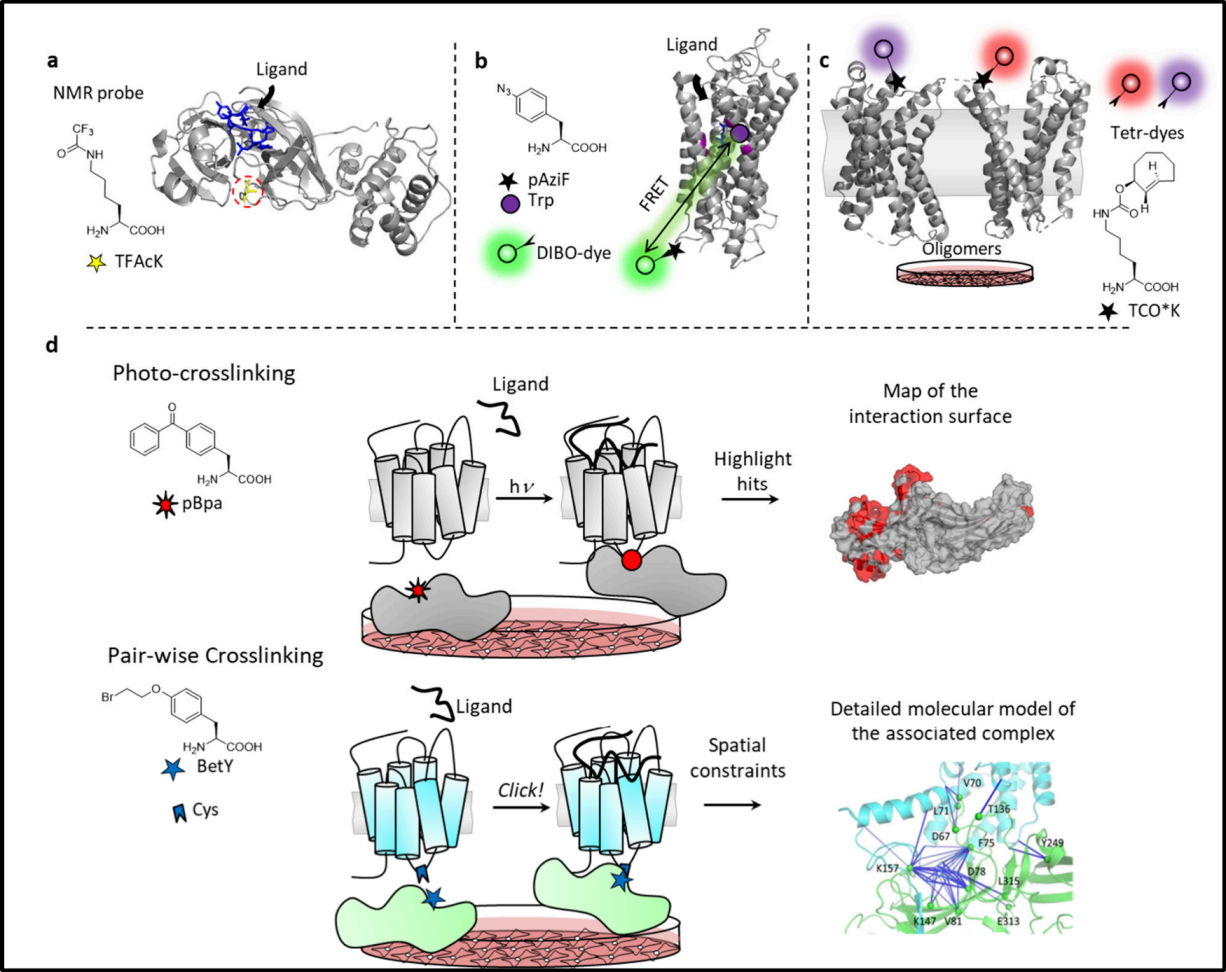
GCE applied to elucidating protein–ligand and protein–protein interaction studies. (a) Fluorinated ncAA (TFAck**26**) incorporated into SARS CoV-2 main protease M^pro^enables NMR to monitor the ligand-binding site (PDBID: 7RNW). (b) Biorthogonal handle ncAA (pAziF**32**) is incorporated into membrane receptor (PDBID: 1U19) and labeled with a fluorescent dye (DIBO-dye) using click chemistry. Canonical AA (Trp) acts as a donor resulting in changing Förster resonance energy transfer (FRET) upon ligand binding. (c) Visualization of oligomers of membrane receptors (CRF_1_R) (PDBID: 4K5Y) using bioorthogonal labeling of AA (TCO*K**143**) by fluorescent molecules. (d) Photo- and chemical cross-linking ncAAs (pBpa**42***, BetY***68**) are introduced into membrane proteins in live cells and used to map binding interface with arrestin (top) (PDBID: 4JQI) and the spatial constraints of the interaction (bottom). The map of the interaction surface and the molecular model are taken from Nat. Commun.2023, 14 ( (1), ), 115136859440 10.1038/s41467-023-36797-2PMC9977954.^389^ Figures showing a cell culture dish refer to studies conducted in live mammalian cells.

NcAA probes for NMR and EPR have been applied to study protein-ligand interaction and to probe the binding pockets of proteins (Figure 7a). The ncAAs pO-tfmF **19**, ^15^N-pOMeF **11** and ^15^N-oNBY **84** were first incorporated in different positions of human fatty acid synthase (FAS-TE) for NMR analysis. The changes in their chemical shifts upon ligand binding helped identify the binding site and ligand-induced conformational changes.^118^ Later, applications were extended to O-*tert*-butyltyrosine (Tby **16**), which was reported to give a satisfactory signal when used in NMR studies and has been useful when monitoring proteins with high molecular weights (in this case the *Bacillus stearothermophilus* DnaB hexamer),^115^ as well as for measuring the dissociation constant of the complex between glutamate and the aspartate/glutamate binding protein in *E. coli*. Compared with fluoro-ncAAs, which exhibit large chemical shift anisotropy due to the fluoride spin, the signal peaks of Tby were much sharper and located in a more favorable chemical shift region than previously reported ncAAs.

Furthermore, protein–proteins interactions have been explored with GCE and NMR in the case of cytosolic proteins and GPCR-arrestin complexes. The interaction in bacterial mitogen-activated protein kinases MEKK3 and MEK5 was studied by incorporation of tfmF **19** and the point of interaction between the two was found, confirming the view from the crystal structure of the complex.^391^ The selectivity of β-Arr1 for phosphorylated positions in vasopressin 2 receptor (V_2_R) has been studied instead with F_2_Y-ncAAs **21**, revealing different conformational states.^392^

An improvement for NMR analysis was the addition of a Si or S atom to the side chain of ncAAs. TMSf **24**(114) shows chemical shifts in a region with very few signals and has therefore been used to monitor protein changes upon ligand binding. By using TMSK **27** and derivatives, the broader application of these ncAAS was demonstrated. TMSK was incorporated in *Bacillus stearothermophilus* (*Bst*) DnaB hexamer, allowing for site-specific changes in the conformation to be detected upon ligand binding.^122^ Ligand binding was also probed with TMSK derivatives on large proteins. Recently, the homodimer of SARS CoV-2 main protease M^pro^ was studied by incorporation of TMSK and other new ncAAs (TFAcK **26** and TMSNK **28**) at different solvent-exposed binding sites (Figure 7a). The chemical shifts of these AAs were registered upon ligand binding of three molecules. In this case, the binding of cyclic peptide inhibitor 1 is known to elicit allosteric effects that are not possible to map via regular NMR techniques. At the same time, the ligand binding site was identified, and the problem of resonance assignment was addressed.^123^ Ligand binding was also explored for the Zika Virus Protease (ZiPro)-inhibitor complex by following the signal of the minimally perturbing Trp analogue 7-FY **23**.^120^ Finally, SF_5_F **22** was incorporated into the *E. coli* peptidyl-prolyl *cis*–*trans* isomerase PpiB as a structural probe to confirm intramolecular short distance contacts in NOESY spectra.^110^

EPR spectroscopy was also used to elucidate enzyme–substrate interactions. For example, the resonance of *p*-amino-phenylalanine pAF **132** and 2,3,5-trifluorotyrosine (F_3_Y 21) was used to monitor production of tyrosyl radicals and their reactivity in subunits α2 and β2 in class Ia ribonucleotide reductase (RNR) in *E. coli*.^119,133^

#### 3.2.2 Fluorescence Microscopy

The environmental sensitivity of fluorescent ncAAs has been exploited for studying protein interactions with ligands or transducers. For example, fluorescence changes of Anap **5** have been monitored in the human acid-sensing ion channel-1a (hASIC1a) in live mammalian cells upon binding of toxin mambalgin-1.^393^ In another study, voltage-clamp fluorimetry on the glycine receptor chloride channel captured the patterns in current and fluorescence responses induced by ligands of different efficacies^394^ and correlated them with unique movements of receptor loops. Anap has also been chosen as a donor for FRET studies in combination with GFP or YFP acceptors. Incorporation of Anap in Bax and YFP in Hsp70 has aided the study of their dissociation during apoptosis in live mammalian cells.^395,396^ In a similar fashion, the energy transfer between the Anap-ATP sensitive K^+^ channel (K_ATP_) and fluorescently labeled TNP-ATP was used to elucidate the role of a nucleotide-binding site of the accessory SUR1 subunit to the potassium channel^397^ and later of the Kir6.2 subunits.^398^

The binding of an antibody to a protein has also been studied with fluorescent AAs by incorporating a Coumarin into protein CD40 ligand and monitoring the fluorescence change upon binding of alfa-CD40L 5c6.^399^ A FRET sensor was also installed between a Trp on antibody Fab30 and Coumarine **3** on β-Arr1 to observe the binding of the two proteins to elucidate the roles of different phospho-sites.^400^ Antibody binding sites for anti-GPCR antibodies have been mapped.^401^ Incorporation of pAziF **32** into an ECL of C-X-C chemokine receptor 4 (CXCR4) enabled the mapping of interaction residues with the mAb 12G5.^402^ Epitopes of the mAbs PRO 140 and 2D7, inhibitors of HIV-1 cellular entry, were also mapped in the same manner. Peptides also benefit from this approach. For example, pBpa **42** and pAziF incorporated into CXCR4 was used to identify AA contacts for the HIV-1 coreceptor blocker, a fluorescent analogue of T140 peptide.^403^

The binding of peptides to proteins was studied by incorporation of Acd **6** into CaM in *E. coli* and FRET/LRET pairing to Mcm, Trp and Eu^3+^, allowing for measurement of apparent *K*_d_ values.^102^ Protein–protein interaction between RecA, a DNA damage sensor, and repressor LexA was monitored with Acd in *E. coli*, and a kinetic model of the complex formation was deducted, showing which residues of LexA are necessary for the recognition interface.^404^

Labeling of membrane receptors has been carried out in experiments to monitor binding of several ligands. For example, pAziF **32** was incorporated into rhodopsin and clicked to an alkyne-dye. This allowed measurement of the kinetics of ligand binding and the converse dissociation reaction in *in vitro* experiments by quenching of the fluorophore.^405^ Incorporation of pAziF in rhodopsin and clicking of a dye was also useful for FRET measurements to measure the kinetics of ligand binding of the 11-*cis*-retinylidene chromophore by engineering a donor–acceptor FRET pair between the dye and an endogenous Trp residue used as donor (Figure 7b). The binding of the ligand was observed to happen upon entrance of the ligand between a specific intramembranous pathway between TM helices five and six of rhodopsin, which had not been demonstrated earlier.^406^

GCE and click-labeling have also been applied in studies of the oligomerization of GPCRs in membrane bilayers. TCOK **142** was incorporated in live mammalian cells in the CRF_1_R, and dual-color competitive labeling was performed. CRF_1_R was shown to form oligomers using a variety of fluorescence and FRET techniques (Figure 7c).^346,407^

#### 3.2.3 Cross-linking

NcAAs for photo- and chemical cross-linking have seen widespread applications for probing protein–protein, enzyme–substrate, or receptor–ligand interactions, given their unique capability for “capturing” molecules in proximity with the protein of interest (Figure 7d). As mentioned above, cross-linking ncAAs and their application have been recently reviewed elsewhere.^85,242,243^ Therefore, we will summarize here only the applications on membrane proteins.

For instance, photo-cross-linking experiments in *E. coli* with pBpa helped visualize the interaction sites between SecA ATPase and the SecYEG translocon and showed that the C6 tail of SecY binds to the active form of SecA.^408^ In another example, the mechanism through which lipopolysaccharide (LPS) is translocated to the outer membrane by lipopolysaccharide transport (Lpt) proteins was investigated. Incorporation of pBpa in several positions of the Lpt proteins led to finding which residues are involved in the binding of LPS.^409,410^ By incorporation of pAziF into the potassium channel Kir6.2, it was possible to understand the interaction between this protein and the other subunits that form the octameric ATP-sensitive potassium channel K_ATP_.^411^

Photo-cross-linking ncAAs have also been successfully applied to the study of interactions occurring at membrane receptors, both with their ligands and with other proteins. pBpa was incorporated in positions proximal to the ligand-binding pocket of the Src homology 2 (SH2) domain of adaptor protein Grb2 and cross-linked with transiently expressed epidermal growth factor (EGF) receptor in the presence of EGF stimulus^412^ in mammalian cells. The results helped highlight which residues are essential for the binding of the two proteins. Later, *p*-trifluoromethyl-diazirinyl-l-phenylalanine (TfmdF **43**) was also installed in the same system and confirmed the findings.^137^

The binding of different natural neuropeptide ligands to CRF_1_R was studied by incorporation of pAziF and cross-linking experiments in mammalian cells. The residues involved in ligand binding were found and a common binding mode was observed in the N-terminal domain for different ligands, validating hypotheses from structure–activity relationship (SAR) studies.^326^ In the case of the human glucagon-like peptide-1 receptor (GLP-1R) in mammalian cells, the binding site of exendin-4 on the N-terminus was mapped with pAziF and the cross-linking results were compared with the crystal structure to generate a molecular model of the peptide binding.^413^

Another member of class B GPCRs, the calcitonin receptor-like receptor (CLR), was studied with photo-cross-linking by incorporation of pAziF in the ECLs. By stimulating the receptor with its known peptide ligand, the calcitonin gene-related peptide (CGRP), the cross-linking of the two partners highlighted the residues responsible for the binding of the peptide to the extracellular domains of the receptor, specifically in ECL2, and a binding model with the peptide at the top of the TM bundle was proposed.^414^

Another binding site of a GPCR was observed in a similar way by incorporation of pAziF and pBpa into CC chemokine receptor type 5 (CCR5) and cross-linking experiments upon binding of the ligand maraviroc in mammalian cells. The results helped highlight the positions in the transmembrane helix bundle involved in the binding of the drug.^415^ pBpa was also incorporated in mammalian cells into neurokinin-1 (NK1) receptor to map which residues in the N-terminal domain and extracellular loop 2 (ECL2) are involved in ligand binding with substance P.^416^ The metabotropic glutamate 2 receptor (mGluR2) was also studied by incorporating pAziF at different positions in the TM domains, showing hit residues in TM4 that contribute to the formation of the heterocomplex with serotonin 5-HT2A receptor (5-HT2AR).^417^ Moreover, the hormone-binding surface on the insulin receptor (IR) was studied by incorporation of pAziF in the amino-terminal tail and carboxyl-terminal tail. The comparison of cross-linking data with the crystal structure showed a previously unknown recognition role of the carboxyl-terminal tail in receptor activation.^418^ The binding site of the human serotonin transporter (hSERT) was also explored with pAziF to elucidate how several drugs bind to the protein and at which sites they bind. Results showed that both inhibitor drugs (paroxetine and escitalopram) bind exclusively to the high-affinity binding site.^419^

Transient protein–protein interactions were also studied by incorporation of photo-cross-linkers in *E. coli* and mammalian cells. *N*-Methylpyrroletetrazole-lysine (mPyTK **57**) was incorporated in adapter protein Grb2 and gave important cross-linking results with EGFR in a stimulus-dependent manner.^150^ The interaction of GPCRs with β-arrestins at the intracellular side of the plasma membrane^420^ has also been extensively investigated with cross-linking ncAAs. For instance, pAziF was introduced in the intracellular loops (ICLs) and carboxyl-terminal tail of angiotensin II type 1 receptor (AT_1_R), the receptor was stimulated, and photo-cross-linking was performed. Results showed a difference in binding patterns among ICLs, as well as when applying biased ligands.^421^ In a complementary manner, photo- and chemical cross-linkers were incorporated into β-Arr1 and β-Arr2 and the receptor footprints of vasopressin receptor 2 (V_2_R), CRF_1_R and PTH_1_R with arrestin were collected. Results showed unique binding hits for each receptor-arrestin complex, delineating once again the extreme flexibility of arrestin in live cells.^390,422^ In another study, pAziF was incorporated into the L-type membrane amino acid transporter 3 (LAT3) and used to identify weak and transient interactions between this transporter and other proteins in its vicinity, resulting in the discovery of 30 new interactions in mammalian cells.^423^

The combination of systematic photo-cross-linking scanning with chemical cross-linking has emerged as a powerful tool to derive structural information about protein–ligand and protein–protein complexes from the live cell context. In a first step, the surface of the protein of interest is mapped with a photo-cross-linking ncAA, which reveals the positions of the protein that lie close to the interaction partner. In the second step, photo-cross-linking hits are substituted with a chemical cross-linker and combined with set systematic sets of Cys mutants of the partner protein (pairwise cross-linking). In this way, it is possible to identify intermolecular pairs of proximal amino acids. This information provides spatial constraints for building accurate molecular models of the associated complexes, which are very useful, especially when no 3D structures are available. The strategy has been very successfully applied to GPCR studies. Systematic photo-cross-linking mapping of the whole juxtamembrane domain of the CRF_1_receptor with pAzF and further incorporation of the chemical cross-linker *p*-2′-fluoroacetylphenylalanine (Ffact **65**)^156^ provided a data set of ligand–receptor spatial constraints for building a 3D model of the Ucn1-CRF_1_R complex.^51^ By applying the same approach to complexes with other ligands, a different binding mode for agonist and antagonist was unveiled, which implies the stabilization of distinct transmembrane (TM) domain conformations.^424^ At the intracellular site, the interaction between the PTH_1_R and β-arrestin1 was investigated using the same strategy. Hundreds of spatial constraints between receptor and arrestin were derived, which were applied to build a molecular model for the associated complex. Importantly, the model covered regions of the complex that are not resolved in any published structure of arrestin-bound GPCRs and allow a glance into the dynamics of the complex.^389^ We envision that this strategy could become an important complement to cover blind spots in crystallography and cryo-EM structures.

Another perspective application of chemical cross-linkers for membrane protein studies is the stabilization of low affinity and transient interactions, for instance, for structural studies. Proof of principle of the approach has been demonstrated on the small GTPase Rab1b. BrC_n_K **77** was incorporated in Rab1b to stabilize the complexes of GDP-bound Rab1b:DrrA_340–533_ with a covalent bond. In this way it became possible to solve the crystal structure of the complex, which was previously not accessible.^162,425^

### 3.3 Drug Discovery

GCE can be used for elucidating the fundamentals of protein structure and function, as well as for drug discovery. One of the most recognizable applications is for the identification or validation of protein–ligand interactions (PLIs) or drug-binding sites on membrane proteins using photo-cross-linking ncAAs. For example, UV-cross-linking using ncAAs was used to identify key interacting residues in the HIV-1 coreceptor, C–C chemokine receptor 5 (CCR5), and its small molecule allosteric drug maraviroc.^415^ Photo-cross-linking has even been used to discern the different binding modes of two distinct antidepressant drugs, mipramine and vortioxetine, to the human serotonin transporter (hSERT).^426^ One of the key features of this type of drug-binding-site mapping is the minimal perturbation caused by the introduction of the small photo-cross-linking ncAAs, thereby allowing for the probing of binding interactions in native cellular environments as described above.

GCE can be used to generate BRET or FRET pairs, enabling live-cell assays to monitor PLIs or PPIs. One noteworthy example was when a bioorthogonal ncAA was used to fluorescently label extracellular loops of the frizzled (FZD) GPCRs that were fused to Nluc on their N-termini.^427^ GCE and bioorthogonal fluorescent labeling can also be combined with alternative tagging strategies including enzymatic, tagging resulting in FRET sensors to monitor ligand- or drug-induced conformational changes in receptors. Specifically, GLP_1_R fused to a SNAP tag was expressed in cells and subsequently labeled using GCE and bioorthogonal coupling of a fluorophore.^428^ These sensitive assays can be used along the drug discovery pipeline to identify lead compounds that bind to membrane proteins or modulate PPIs in their native environment of the cell.

**Figure 8:**
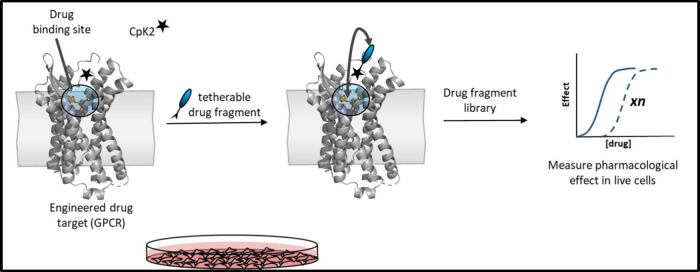
Applications of GCE for drug discovery. Bioorthogonal handle AA (CpK2, **149**) is introduced into a receptor expressed in live cells at an AA residue proximal to a drug binding site. Low-affinity molecules or drug fragments (blue oval) attached to reactive functional groups undergo site-specific covalent coupling with the receptor (PDBID: 4MBS), enhancing the local concentration of molecule and protein occupancy sufficiently, resulting in a measurable pharmacological effect in live cell assays.

Beyond measuring dynamic changes in live cells, GCE has the potential to be used directly to enable cell-based pharmacological screening of low affinity compounds such as drug fragments. Recently, GCE was used to bioorthogonally tether analogues of the allosteric drug maraviroc to CCR5 engineered to contain the ncAA CpK2 **149** in live cells^429^ (Figure 8). A measurable pharmacological effect of low affinity maraviroc analogues conjugated to a bioorthogonal handle was not detected in cells expressing nonengineered CCR5 in a calcium flux assay at concentrations up to 10 μM. However, when the same analogues were added to cells expressing CCR5 harboring a CpK, the analogues covalently reacted with the receptor and were able to inhibit agonist-induced calcium mobilization. While this approach has not been applied to drug screens that we know of, site-specific covalent tethering of fragments with high ligand-efficiency could be employed to probe the druggability of targeted regions on receptors in live cell assays and to couple compound binding with a pharmacological readout. This has the potential to shift the paradigm of drug discovery approaches from binding-based biophysical assays for hit finding to function-based screens in the “native-like” cellular context.

Nevertheless, while GCE is a powerful technology, its applications in drug discovery remain heavily focused on site-specific engineering of mAbs, a topic beyond the scope of this review. In particular, introduction of bioorthogonal handles into defined regions of Abs enables generation of next generation chemically defined Ab-conjugates to improve the efficacy, potency, and stability of these compounds for applications as therapeutics.^430^ Continued improvements in GCE technology toward more efficient amber codon suppression systems in live mammalian cells will undoubtably increase the adoption of this powerful methodology in driving drug discovery.

### 3.4 Photocontrol of Function

**Figure 9:**
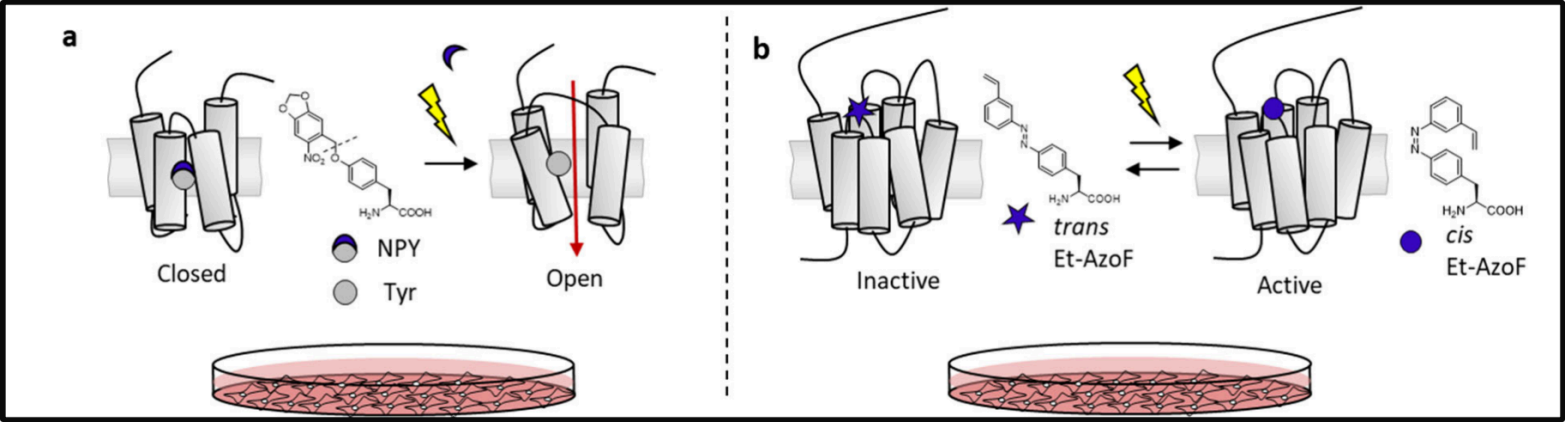
GCE enables photocontrol of receptor function. (a) A photocaged-Tyr (NPY **96**) introduced in a channel receptor is unmasked using light, leading to channel activation. (b) Photoswitchable ncAA (Et-AzoF **103**) introduced into glutamate receptor isomerizes upon irradiation to study the activation and inactivation of the receptor. Figures showing a cell culture dish refer to studies conducted in live mammalian cells.

Light is a powerful tool that can be employed for studying biological processes. In past decades with the advent of optogenetics, photosensitive moieties have been incorporated in many proteins, peptides and ligands to perform a variety of studies, mostly *in vivo*, where traditional methods are not always applicable. While channel rhodopsins, optoXRs and other light-sensitive domains have provided invaluable insight of the neuronal network, these techniques are presenting several disadvantages, mainly their bulkiness, lack of residue-specificity and difficulty of handling.^431^ Incorporating photocages or photoswitches in a protein offers an answer to this problem. It can be applied on both cytosolic and membrane proteins and is, once again, minimally invasive while still offering temporal and spatial control of protein activation (Figure 9).

#### 3.4.1 Photocaging

Photocaged AAs have been incorporated in positions necessary for protein function with respect to binding of substrates. Upon light irradiation, the ncAA decages and turns into a native AA, therefore resetting the original binding activity of the protein. Applications of photocaged AAs have focused mainly on enzymes and cytosolic proteins. Of particular interest, they have been used as a tool to study cell signaling pathways. In this case, light activation can be an advantage over knock-down or knockout techniques, given their easy handling and decaging process. Furthermore, the incorporation of a photocage is minimally invasive with respect to cellular health compared to a complete knockout of a signaling protein.^432^ Photocaged ncAAs have not been incorporated into many membrane proteins yet, but they have great potential for future applications.

Proof-of-principle was shown in yeast for human proapoptotic protein caspase 3 (Cas3), where *o*-nitrobenzyl-**Cys** (o-NBC **83**) was introduced in the active site in place of a regular Cys. The introduction of the ncAA prevented the formation of the active protein from the inactive zymogen, which was recovered after irradiation, resulting in the conversion of ca. 40% of o-NBC in Cys.^48^ o-NBC, caged-Cys **94** and caged-hCys **100** were also incorporated into TEV protease and firefly luciferase, and photodeprotection was carried out in single mammalian cells.^178,180^ NcAAs with photocaged groups were also used for the study of potassium channels: 4,5-dimethoxy-2-nitrobenzyl-cysteine (DMNB-Cys, *i.e*., Cmn **90**) was incorporated in the pore of inwardly rectifying potassium channel Kir2.1 in mammalian neurons. The activation of the channel by irradiation allowed neuronal firing, showing spatial and temporal control over activation.^174^ An analogue of caged-Cys was also developed, caged-seleno-Cys (DMNB-SeC **93**). Seleno-Cys is important for the study of seleno-proteins but is unfortunately highly reactive in the cellular environment. By caging the side chain, the stability of the residue is ensured until photodecaging occurs.^177^

A **Tyr** analogue, *o*-nitrobenzyl-O-tyrosine (o-NBY **84**) was incorporated in *E. coli* in β-galactosidase in place of an essential Tyr. The activity of the enzyme in live cells was shown to drop completely in the presence of the ncAA in live cells but was easily restored to almost 70% of wt after 30 min of irradiation.^167^ In another study, *o*-NBY was incorporated in a position of the *Taq* polymerase in a position involved in the binding of dNTPs. An application of this photocaged *Taq* was shown in hot-start PCR, where the polymerase is blocked until the reaction reaches a temperature where nonspecific annealing of primers is not possible. Comparison with regular *Taq* showed one clearer band in gel electrophoresis as opposed to a smeared collection of several bands.^433^ Similarly, the T7 RNA polymerase was photocaged in *E. coli* and mammalian cells by introducing oNBY in the nucleotide binding site. Only after irradiation was the activity of the enzyme restored both *in vitro* and *in vivo*, which allowed spatiotemporal control of gene function.^434^ A light-activatable Zinc-finger nuclease (ZFN) was similarly produced in mammalian cells by incorporation of oNBY close to the active center of the nuclease, in mammalian cells.^435^ oNBY was incorporated into a signal transducer and activator of transcription (STAT1) to exert photocontrol on Tyr phosphorylation.^436^ Temporal and special control over the TEV protease was also performed by incorporation of oNBY and derivatives NPY **96**, MNPY **97** and oNPPY **87**.^170^ In a similar fashion, oNBY was incorporated into the Cre recombinase in *E. coli* and recombination activity was observed only after 5 min of irradiation. In mammalian cells, it was possible to exert a spatial control in decaging, and therefore activity, by irradiation of a small area of sample.^437,438^ Of particular notice, oNBY was applied to the study of membrane-bound tyrosine kinase A (TrkA) receptor in mammalian cells to discover its Tyr phosphorylation-site-dependent pathways of activation and signaling.^439^

A photocaged **Ser**, 4,5-dimethoxy-2-nitrobenzylserine (DMNB-Ser **92**) was introduced in transcription factor Pho4 in yeast to analyze its function. When naturally phosphorylated Ser residues were changed to the ncAA, nuclear export of Pho4 was inhibited and was reactivated *in vivo* upon irradiation. The kinetics of the process were monitored for each position, revealing distinct patterns and a preference in phosphorylation sites.^176^

A photocaged **Lys** (PCK **98**) was incorporated in the nuclear localization sequences (NLSs) of nucleoplasmin in mammalian cells. Installing PCK in a position involved in the binding of importin-α, the protein was relocalized in the cytosol. Upon irradiation and decaging, nuclear import of the protein was observed, and the kinetics of the process were measured. Similarly, control of localization of the nuclear import of the tumor suppressor p53 revealed a protein localization in the cytosol with the caged Lys, which was then shifted to the nucleus after 5 s of irradiation and decaging.^179^ Photocaged Lys was also incorporated in the NLS of transcription factor SATB1, and nuclear import was only observed after UV illumination to cleave the protecting group. This was applied to the observation of both the transcription activity of FOX3 and cleavage of proteins by the TEV protease.^440^ To study the kinetics of signal transduction within a pathway, PCK was incorporated in the MEK1 kinase at a site in the binding pocket for ATP. Decaging of the AA allowed for fine-tuning of the cellular concentration of active kinase by changing the illumination time, in both mammalian cells and zebrafish. The activation of MEK was demonstrated to lead to activation of ERK1/2.^441,442^ PCK was also site-specifically incorporated into Cas9 to exercise spatial and temporal control over gene editing,^443^ as well as into T7 RNA polymerase in mammalian cells to control gene activation^444^ and in Cre recombinase for spatial control of gene function.^438^ The Tyr kinase LCK was photoactivated by incorporation of PCK in its active site and the kinetics of phosphorylation were then quantified in mammalian cells.^445^ Furthermore, isocitrate dehydrogenase (IDH2) was mutated at a position known in cancer by incorporating PCK, and the rapid production of oncometabolites was measured after irradiation, elucidating the link between cancer development and protein mutations.^446^ The activity of phosphatase 6 (DUSP6 or MKP3) was investigated via decaging activation experiments with HCK **3** and caged Cys Cmn **90**, revealing a regulation of ERK nuclear translocation in response to an increase in MKP3 activity.^447^

In a different kind of application, to answer the need for turn-on fluorescent probes,^448^ a photocaged **Glu** (NPE **95**) was incorporated into GFP at a critical residue for chromophore formation, therefore suppressing fluorescence of GFP. Only after decaging was the fluorescence reinstated. This approach had been previously used at Tyr66,^449^ but this residue is not as highly conserved as Glu222 in all fluorescent proteins.^171^

The fluorescent AA CoumLys **3** also exhibits photocaging traits. In the case of firefly luciferase, a loss of catalytic activity was observed by incorporation of **3** into the protein, which was promptly restored after UV irradiation and decaging of the coumarin moiety. The decaging was monitored by following the loss of fluorescence intensity, while protein activation was measured by the increased luminescence signal.^99^

Overall, it is important to stress that decaging of photocaged AAs is an irreversible process and does not afford the possibility of turning a process on and off again. Additionally, once the protecting group is removed from the AA, it can sometimes react with other molecules in the cellular environment and could potentially create reactive species that can bind to other residues and reduce or modify protein activity. Furthermore, caged AAs only produce a local perturbation that might not be relevant for the total protein conformation, necessary for protein function.^432^

#### 3.4.2 Photoswitches

Photoisomerizable AAs, on the contrary, retain all their parts, but only change their conformation upon irradiation in a reversible manner. These ncAAs feature double bonds that are preferably in the *trans* conformation, as it is the least spatially hindered and thus the most energetically favored. Upon light-irradiation, the system has enough energy to switch to the more compact and less stable *cis* isomer.^450^ Most photoswitchable AAs are based on an azobenzene moiety, due to its small size, stability, fast isomerization kinetics and high quantum yield.^451^ By irradiating light at different wavelengths, it is possible to induce the switching of the isomer back and forth between *trans* and *cis*.^452^ A proof-of-principle study initially showed the incorporation of phenylalanine-4-azobenzene (AzoF **101**) in *E. coli*. AzoF can be reversibly shifted between its *trans* and *cis* forms and, when placed close to an active site, can modulate the affinity of the protein for a ligand or other substrate. AzoF was incorporated in the catabolite activator protein (CAP), which, upon binding with cAMP, enhances transcription by binding to its promoter. cAMP binding was tested in the presence of *trans* and *cis* isomers of the AA, and a difference was calculated in binding constants for the two, with the *cis* form exhibiting the least affinity for cAMP.^181^ Photoisomerizable analogues of AzoF were synthesized, carrying an additional reactive group for click reactions (termed Photo-Switchable Click AAs, PSCAAs) on the benzene ring **102**–**107**. When clicked intramolecularly to a Cys, they can serve as bridges between different domains of a protein, thus allowing photocontrol of its conformation. Incorporation of these ncAAs into proteins was achieved in *E. coli* and mammalian cells.^453^

For instance, incorporation of Cl-ncAAs **104**, into CaM resulted in an additional bond with a Cys in the protein, which isomerized upon irradiation, therefore changing the conformation of the protein.^182^ A pentafluoro-derivative of AzoF **105** was also incorporated into CaM and the binding of CaM to neuronal nitric oxide synthase (NOS-I) was modulated by switching the AA between the Z and E conformation after irradiation. Furthermore, AzoF derivatives such as F_2_AzoF and F_4_AzoF were incorporated in mammalian cells into firefly luciferase and the FLuc activity was modulated by UV irradiation with up to five cycles of on/off switching.^454^ The incorporation of photoswitches has also been relevant in the studies of allostery. AzoF was incorporated in several positions in imidazole glycerol phosphate synthase, an enzyme that exhibits large conformational rearrangements with allosteric binding. By light activation, it was possible to control allosteric activation of the enzyme.^455^ The use of photoswitches for the study of membrane receptors has also been particularly important for ligand-gated ion channels.^456^ In recent years, the alkene-derivative of AzoF **103** was incorporated into the glutamate receptor and the receptor activity was switched reversibly on and off by illumination with UV light. The study allowed visualization of real-time molecular rearrangements.^457^ However, only very few studies have focused on GCE and incorporation of these photoswitches in membrane receptors, since the system for incorporation of these ncAAs is recent. Instead, most receptor studies have clicked photoswitch moieties to Cys residues.^458^

Photoswitches and photocages both present the drawback of not complete conversion after irradiation. This means that often a significant population of protein will stay in its inactivated state. Additionally, photoswitches are subjected to relaxation of the system and natural isomerization to *trans*, which make them unreliable for long-time measurements. Photoswitches are also quite bulky and, when positioned next to a binding site, can decrease the binding affinity of a ligand or transducer with the protein of interest.

### 3.5 Other Applications

GCE and ncAAs have been important tools in the study of post-translational modifications (PTMs) of proteins. PTMs consist of the addition of chemical moieties such as glycosylation, acylation, sulfation, phosphorylation, and others to the protein backbone after translation or cotranslational. Most post-translational modifications involve AAs that carry a functional group, that become later the substrate for enzymatic activity. For instance, glycosylation, phosphorylation and lipidation are all PTMs. Since PTMs are not directly “written” in the genetic sequence of the protein and require the cellular machinery to happen, cell-free or synthetic approaches cannot always be applied to produce proteins with PTMs. This is disadvantageous for studies that require high amounts of purified protein, as well as for studies investigating the importance of certain PTMs over others.

GCE has provided a robust alternative to tedious and unreliable *in vitro* enzymatic reactions, by directly expanding the chemistry of the protein during translation.^459,460^ The applications of ncAAs for PTMs studies were first demonstrated by incorporation of sulfotyrosine (SY **150**) in the protein hirudin, a natural inhibitor of thrombin, in *E. coli*. This method yielded good amounts of the PTM-protein, which was previously difficult to obtain with recombinant methods and is used as an anticoagulant.^211^ More recently, the AA was also incorporated into chemokine receptor CXCR4 to understand the role of this PTM in the receptor activation.^461^ Other Tyr PTMs have also been incorporated by GCE. For example, the effect of nitration of the AA by reactive-nitrogen species, generated in cellular diseases, was also studied by incorporating mNO_2_Y **89** in Hsp90 and monitoring the toxic effects of this modified protein.^462^ Phosphorylation has also been investigated with ncAAs by incorporating phospho-Tyr, -Ser and -Thr residues in different proteins to probe the importance of phosphorylation sites in intracellular signaling.^217,219^ In this fashion, phosphorylation sites involved in the signaling cascade were highlighted in different proteins in both *E. coli* and mammalian cells. Several Lys residues have also been used for investigating PTMs in histones, since Lys PTMs are often associated with chromatin formation. To this end, N^ε^-acetyl-Lys (Kac **151**), N^ε^-crotonyl-Lys (KCr **155**), N^ε^-propionyl-Lys (Kpr **153**), N^ε^-butyryl-Lys (Kbu **154**), and N^ε^-2-hydroxyisobutyryl-Lys (HibK **158**) were all successfully incorporated into histones H2B, H2A and H3.^59,188,213,216^

GCE also has a crucial role in the evolution of proteins with special characteristics. Incorporation of AAs such as cross-linkers can enhance, for example, the stability of proteins and increase their melting temperatures. This was done in bacteria by incorporating pBpa (**42**) into homoserine O-succinyltransferase (metA), which resulted in an increase of melting temperature.^463^ In a following study, the same protein was expressed incorporating *p*-isothiocyanate-Phe (pNCSF **76**), and the melting temperature was increased further.^464^ Other ncAAs applications involve their use for functionalization of surfaces and other biorelevant materials. Dithiolane containing AA (dtF **182**) was incorporated in sfGFP as a proof-of-principle application and covalently bound to gold nanoparticles, providing itself as a superior alternative to Cys-analogues with respect to chemical durability.^222^ Another interesting application of noncanonical AAs has been in the modulation of fluorescence in fluorescent proteins. Histidine-derivatives were incorporated into BFP,^65^ while TerphenylPhe (TerphF **10**) was incorporated at key residues of GFP in *E. coli*.^106^ In both cases, the fluorescence of the protein was successfully modulated by introduction of these ncAAs. Other applications of ncAAs have seen the use of the biorthogonal handle on the protein for single-step purification, as in the case of *p*-boronophenyl-Ala (pBorF **127**).^196^

## 4 Conclusions and Outlook

In this review, we have presented some of the advantages that GCE-enabled ncAA mutagenesis offers for the study of proteins both *in vitro* and in live cells. The technique is versatile and is compatible with most tools employed for the study of protein structure, function and interactions. The introduction of fluorescent probes has been particularly valuable. The development of robust techniques and technologies (*i.e*., single-molecule and super-resolution imaging, ultrafast devices for kinetic studies) requires introduction of finely tuned and specific modifications in proteins, a need that has been matched by development of ncAAs and complementary bioorthogonal labeling chemistries. Additionally, it is important to emphasize the impressive steps taken not only to expand the genetic code in single cells but also to develop expanded codes in model organisms able to synthesize the “extra” AA and to incorporate it into proteins. While concrete applications for these systems are still not generally available, it is not impossible to imagine a future where GCE with inputs from synthetic biology will have produced organisms with “non-canonical” properties that can be exploited for multiple applications, from development and synthesis of drugs and polymers to the expression of stable proteins resistant to degenerative reactions in the cellular environment.

Advances in the elucidation of membrane protein structure and function over the past three decades have required multidisciplinary approaches, including application of molecular cloning and expression, development of high-throughput miniaturized cell-based second messenger assays, specific enhancements of structural methods such as X-ray crystallography and single-particle cryo-EM, and refinement of advanced computational methods. The contribution of GCE to membrane protein research has been increasing over the years. Efficient GCE in membrane proteins remains challenging though, especially for those targets such as GPCRs, which are expressed at low levels in mammalian cells and often also in compatible heterologous expression platforms such as insect cells. While we have witnessed dramatic enhancements of the efficiency of GCE in mammalian cells in culture, with achievable expression yields of ncAA-labeled GPCRs of up to about 20% of wild type levels, application of GCE in insect cells is still in its infancy.

The amount of obtainable protein is a key factor determining which method application can be facilitated using GCE. For instance, the FTIR study on azide-tagged rhodopsin that we have described above (section 3.1.3) was only possible both because this receptor expresses extremely well in mammalian cells and because the orthogonal system that incorporates pAziF in these cells is superbly efficient. On the other hand, it is difficult to obtain large amounts of other GPCRs that would be sufficient, for instance, for ncAA-aided crystallography or NMR studies. In this case, new baculovirus expression vectors for high-yield expression in insect cells are needed. In the meantime, complementary GCE-based cross-linking strategies have already shown an enormous potential to acquire structural information on membrane proteins that are not accessible to classical biophysical methods.

For this application, which relies on immunoblot read out or ELISA-like assays, only very small amounts of probe are necessary. Even in this case, not all membrane proteins give sufficient ncAA incorporation yields for direct analysis of whole-cell lysates. For instance, while we have had very good experience with secretin-like GPCRs and chemokine receptors, some rhodopsin-like GPCRs such as the Y_5_ and even the β_2_-adrenergic receptor have turned out to be more challenging targets for GCE. We envision that a systematic combination of classical biophysical methods and GCE-based cross-linking, which provides both complementary information about flexible protein regions that are not resolved in the structures and a validation of structural hallmarks from the physiological environment of the live cell, could become a standard for characterization of protein structure in the future.

GCE-based labeling of membrane proteins with fluorophores and other biophysical probes also has great potential for addressing dynamic aspects of membrane protein function *in vitro*, but especially in live cells. We already have at hand robust click-chemistry methods to label extracellular domains of membrane proteins, while we still await technical improvements enabling efficient labeling also of intracellular domains, which is still very challenging. We envision that GCE-based post-translational labeling of membrane proteins will facilitate the application of sm detection strategies and the development of cell-based biosensors for the study of membrane protein dynamics at a spatial resolution that is not achievable with any other labeling method. Ultimately, the synergy of all these strategies will lead to new interdisciplinary drug discovery paradigms.

## List of Abbreviations

5-HT2AR: serotonin 5-HT2A receptor
AA: amino acid
aaRS: aminoacyl-tRNA-synthetase
ACCuRET: anap Cyclen- Cu^2+^ resonance energy transfer
ADGRB3: adhesion G Protein-Coupled Receptor B3
ADGRE2/5: adhesion G Protein-Coupled Receptor E2/5
ADGRG1: adhesion G Protein-Coupled Receptor G1
ADGRL1: adhesion G Protein-Coupled Receptor L1
Afb: affibody
Agp2: general amino acid permease-2
AHA2: autoinhibited H^+^-ATPase-2
AMPAR: α-amino-3-hydroxy-5-methyl-4-isoxazolepropionic acid receptor
ASIC1: acid-sensing ion channel 1
AT_1_R: angiotensin II type 1 receptor
ATP: adenosine triphosphate
β-Arr: β-arrestin
Bax: bcl-2-like *protein* 4
BFP: blue-fluorescent protein
BirA: biotin-ligase
BLUF: blue-light using FAD
BRET: bioluminescence resonance energy transfer
Bst: *Bacillus stearothermophilus*
CaM: calmodulin
cAMP: cyclic adenosine monophosphate
CAP: catabolite activator protein
Cas3: proapoptotic protein caspase 3
CBT: cyanobenzo-thiazoles
CCR5: C–C chemokine receptor type 5
CD40: cluster of differentiation 40
Cdk5: cyclin-dependent kinase 5
*C. elegans*: *Caenorhabditis elegans*
*Ci*: *Ciona intestinalis*
*GCRP*: calcitonin gene-related peptide
CLR: the calcitonin receptor-like receptor
cpGFP: circularly permuted GFP
CPR: cytochrome P450 reductase
CRAC: Ca^2+^ release-activated Ca^2+^ channel
CRF_1_R: corticotropin-releasing factor receptor type 1 receptor
CRISPR/CAS9: clustered regularly interspaced short palindromic repeats/CRISPR associated protein 9
Cryo-EM: cryo-electron microscopy
CuAAC: Cu-catalyzed alkyne–azide cycloaddition
CXCR4: C-X-C chemokine receptor type 4
Cy3/5: cyanine3/5
DAGK: diacylglycerol kinase
DEER: double electron–electron resonance
Der1: degradation in endoplasmic reticulum protein 1
DNA: DNA
DnaB: dnaB helicase
dNTP: deoxynucleotide triphosphate
DUSP6: dual specificity phosphatase 6
*Ec/E. coli*: *Escherichia coli*
*ECL*: extracellular loop
EGF: epidermal growth factor
EGFR: epidermal growth factor receptor
ELISA: enzyme-linked immunosorbent assay
EPR: electron paramagnetic resonance
ER: endoplasmic reticulum
ERAD: ER-associated protein degradation
ErbB2: receptor tyrosine-protein kinase erbB-2
eRF: ETS domain-containing transcription factor
ERK1/2: extracellular signal-regulated kinase 1/2
ERp29: endoplasmic reticulum protein 29
Fab: fragment antigen-binding region
FACT: facilitates chromatin transcription
FAS-TE: fatty acid synthase
FlAAs: fluorescent amino acids
FlAsH: fluorescein arsenical hairpin binder-ethanedithiol
FLIM: Fluorescence-lifetime imaging microscopy
FLuc: firefly luciferase
FP: fluorescent protein
FOX3: Forkhead box O-3
FRET: Förster resonance energy transfer
FT-IR: Fourier-transform IR
FtsZ: bacterial tubulin homologue
FZD: Frizzled
FZD-CRD: Frizzled receptor-cysteine rich domain
G-protein: guanine-nucleotide-binding regulatory protein
GalT1: galactose-1-phosphate uridyl transferase
GB1: B1 domain of streptococcal protein G
GCE: genetic code expansion
GCGR: glucagon receptor
GDP: guanosine diphosphate
GECX: genetically encoded chemical cross-linkers
GFP: green-fluorescent protein
GHS-R1a: growth hormone (GH) secretagogue receptor
GLP_1_R: glucagon-like peptide-1 receptor
GPCR: G protein-coupled receptor
Grb2: growth factor receptor-bound protein 2
GroEL: GroEL chaperone protein
Grp94: glucose-regulated protein 94
GST: glutathione S-transferase
H3: histone H3
hASIC1a: human acid-sensing ion channel-1a
HdeA/B: HNS-dependent expression A/B
HDH: histidinol dehydrogenase
Hsp70/90: heat shock protein 70/90
hSERT: human serotonin transporter
hSOD: human superoxide dismutase
H_V_ 1: voltage-gated proton channels
ICL3: intracellular loop 3
IDH2: isocitrate dehydrogenase-2
IEDDA: inverse electron-demand Diels–Alder cycloaddition
IFITM: Interferon-induced transmembrane *protein*
IL-4Rα: interleukin-4 receptor alpha
IR: Infrared or insulin receptor
JunB: transcription factor jun-B
K_ATP_: ATP-sensitive potassium channel
*K*_d_: dissociation constant
Kir: inward-rectifier potassium channel
Kv: voltage-gated potassium ion channel
LamB: maltoporin
LCK: lymphocyte-specific protein tyrosine kinase
LexA: locus for X-ray sensitivity A
LplA: lipoic acid ligase
LPS: lipopolysaccharide
Lpt: lipopolysaccharide transport
LRET: Luminescence Resonance Energy Transfer
LamB: maltoporin
LCK: lymphocyte-specific protein tyrosine kinase
LexA: locus for X-ray sensitivity A
LplA: lipoic acid ligase
LPS: lipopolysaccharide
Lpt: lipopolysaccharide transport
LRET: Luminescence Resonance Energy Transfer
*Ma*: *Methanomethylophilus alvus*
*Mb*: *Methanosarcina barkeri*
Mcm: methoxycoumarin
MD: molecular dynamics
MEKK1/3/5 (MEK): mitogen-activated protein kinase kinase kinase 1/3/5
metA: homoserine O-succinyltransferase
mGluR2: metabotropic glutamate 2 receptor
*Mj*: *Methanocaldococcus jannaschii*
MKP3: MAP Kinase Phosphatase 3
*Mm*: *Methanosarcina mazei*
M^pro^: main *protease*
mRNA: messenger ribonucleic acid
MS: mass spectrometry
MBP: maltose-binding protein
NADPH: nicotinamide adenine dinucleotide phosphate
Na_v_1.5: sodium channel protein type 5 subunit alpha
ncAA: noncanonical amino acid
NK1: neurokinin-1
NLS: nuclear localization sequence
Nluc: nanoluciferase
NMDA: *N*-methyl-d-aspartate
NMR: nuclear magnetic resonance
NOESY: nuclear Overhauser effect spectroscopy
NTD: N-terminal domain
NTR: nitroreductase
optoXRs: genetically encoded optical tools
PAL1: phenylalanine ammonia lyase-1
PCR: polymerase chain reaction
PLIs: protein–ligand interactions
PPIs: protein–protein interactions
PpiB: peptidyl-prolyl *cis*–*trans* isomerase B
PPTase: phosphopantetheinyl-transferase
Pyl: pyrrolysine
PTH1R: parathyroid hormone 1 receptor
PTM: post-translational modification
PSCaa: PhotoSwitchable Click Amino Acids
QBP: glutamine-binding protein
Rab1b: ras-related protein Rab-1B
RAMP1: receptor activity-modifying protein 1
ReAsH: red-fluorescent arsenical hairpin binder-ethanedithiol
Rho: rhodopsin
RhoBo: rhodamine-based bisboronic acid
SAD: single-wavelength anomalous dispersion
SAR: structure–activity relationship
SARS-CoV-2: severe acute respiratory syndrome coronavirus 2
SATB1: special AT-rich sequence-binding protein-1
*Sc*: *Saccharomyces cerevisiae*
SeAcs: acetyl-CoA synthetase
sfGFP: superfolded GFP
SH2: Src homology 2
*Sj*: *Schistosoma japonica*
SlPatA: acetoacetyl-CoA synthetase
sm: single-molecule
smFRET: single-molecule FRET
SPAAC: strained-promoted azide–alkyne cycloaddition
SPANC: strain-promoted alkyne-nitrone cycloaddition
spHCN: hyperpolarization-activated cyclic nucleotide–gated ion channel
SPIEDAC: strain-promoted inverse-electron-demand Diels–Alder cycloaddition
SrtA: sortase A
STAT1: signal transducer and activator of transcription
SuFEX: sulfur-fluoride exchange
SUR1: sulfonylurea receptors 1
TAMM: ((alkylthio)(aryl)methylene)malononitrile
*Taq*: *Thermus aquaticus*
TEM-1: Class A beta-lactamase type 1
TETAC: (1-(2-pyridin-2yldisulfanyl)ethyl)-1,4,7,10-tetraazacyclododecane
TEV: tobacco Etch Virus
TM: transmembrane
TMD: transmembrane domain
tmFRET: transition metal ion FRET
TNP: 2,4,6-trinitrophenol
Tom40: translocase of outer mitochondrial membrane 40 homologue
tRNA: transfer ribonucleic acid
TROSY: transverse relaxation-optimized spectroscopy
TRPM8: transient receptor potential melastatin 8
TRPV1: transient receptor potential vanilloid 1
TRX: thioredoxin
Ub: ubiquitin
Ucn1: urocortin 1
UV: ultraviolet
V_2_R: vasopressin receptor 2
VSP: voltage-sensitive phosphatase
WNTs: Wingless/Int-1 lipoglycoproteins
YFP: yellow-fluorescent protein
ZFN: Zinc-finger nuclease
ZiPro: Zika Virus Protease
Z_SPA_: Z domain of staphylococcal protein A
